# Semiconductive 2D arrays of pancake-bonded oligomers of partially charged TCNQ radicals

**DOI:** 10.1107/S2052252522004717

**Published:** 2022-05-28

**Authors:** Krešimir Molčanov, Valentina Milašinović, Biserka Kojić-Prodić, Nadica Maltar-Strmečki, Jiangyang You, Ana Šantić, Lidija Kanižaj, Vladimir Stilinović, Luka Fotović

**Affiliations:** a Department of Physical Chemistry, Rudjer Bošković Institute, Bijenička 54, Zagreb 10000, Croatia; b Department of Materials Chemistry, Rudjer Bošković Institute, Bijenička 54, Zagreb 10000, Croatia; c Department of Materials Physics, Rudjer Bošković Institute, Bijenička 54, Zagreb 10000, Croatia; d Department of Chemistry, Faculty of Science, University of Zagreb, Horvatovac 102a, Zagreb HR-10000, Croatia

**Keywords:** multicentre two-electron bonding, TCNQ radical anions, pancake bonding, crystal structures, crystal engineering, magnetic properties, electrical properties

## Abstract

Multicentre two-electron (mc/2e or ‘pancake bonding’) bonding between 7,7,8,8-tetra­cyano­quinodi­methane (TCNQ) radical anions was studied on its 14 novel salts with planar organic cations. The formal charges of the TCNQ^δ−^ moieties are −1/2 and −2/3, and they form mc/2e bonded dimers, trimers and tetramers which are further stacked into extended arrays. Multicentre bonding within these oligomers is characterized by short interplanar separations of 2.9–3.2 Å; distances between the oligomers are larger, typically >3.3 Å. The stacks are laterally connected by C—H⋯N hydrogen bonding, forming 2D arrays. The nature of mc/2e bonding is characterized by structural, magnetic and electrical data. The compounds are found to be semiconductors, and high conductivity [10^−2^ (Ω cm)^−1^] correlates with short interplanar distances between pancake-bonded oligomers.

## Abstract 2

Extended arrays of pancake-bonded TCNQ radical anions lead to long-range spin interactions and conductivity in 2D.

## 1. Introduction

π-Stacking of planar organic radicals is known to involve spin pairing of contiguous radicals (Preuss, 2014 ▸; Kertesz, 2019 ▸; Molčanov & Kojić-Prodić, 2019 ▸; Molčanov *et al.*, 2019*c* ▸), as shown by the diamagnetic or antiferromagnetic properties of bulk samples (Molčanov & Kojić-Prodić, 2019 ▸; Molčanov *et al.*, 2019*c* ▸). This implies the formation of a weak covalent interaction (Huang & Kertesz, 2007 ▸; Huang *et al.*, 2008 ▸; Novoa *et al.*, 2009 ▸; Tian & Kertesz, 2011 ▸; Preuss, 2014 ▸; Cui *et al.*, 2014*a* ▸,*b* ▸,*c* ▸; Mou & Kertesz, 2017 ▸; Kertesz, 2019 ▸; Molčanov & Kojić-Prodić, 2019 ▸; Molčanov *et al.*, 2019*c* ▸) with a non-localized electron pair distributed between two rings (Kertesz, 2019 ▸; Molčanov & Kojić-Prodić, 2019 ▸). Commonly used terms for this type of interaction are two-electron multicentre (mc/2e) bonding and pancake bonding (Preuss, 2014 ▸; Kertesz, 2019 ▸). According to quantum chemical models, an HOMO spanning both rings is formed (Kertesz, 2019 ▸) and the covalent contribution to the total interaction may exceed −15 kcal mol^−1^, which is comparable to strong hydrogen bonding (Steiner, 2002 ▸; Kojić-Prodić & Molčanov, 2008 ▸). Therefore, pancake bonding may be regarded as both a strong intermolecular interaction and a weak covalent bond. It is interesting from both fundamental (the nature of chemical bonding) and practical (design of organic magnets and conductive materials) aspects (Podzorov, 2010 ▸; Hicks, 2011 ▸; Sanvito, 2011*a* ▸,*b* ▸; Veciana, 2011 ▸; Ratera & Veciana, 2011 ▸; Morita *et al.*, 2013 ▸; Murata *et al.*, 2013 ▸).

The most important factors for designing functional materials are stability and a low HOMO–LUMO energy barrier, which limits conductivity. It is known that electrons can easily jump between two rings in a pancake-bonded dimer, whereas non-bonding stacking contacts (between the dimers) prevent electron jumping due to high energy barriers (Molčanov *et al.*, 2019*a* ▸). Thus, conductivity can be enhanced if pancake bonding is extended throughout a crystal. We have shown that, in infinite stacks of equidistant radicals, pancake bonding extends not only between a pair of radicals, but throughout a stack (Molčanov *et al.*, 2019*a* ▸,*c* ▸; Molčanov & Kojić-Prodić, 2019 ▸), and such crystals are semiconductive (Molčanov *et al.*, 2016 ▸; 2018*a* ▸). For ionic radical systems, conductivity rarely exceeds 10^−6^ S cm^−1^ (Itkis *et al.*, 2002 ▸; Lekin *et al.*, 2010 ▸; Podzorov, 2010 ▸; Mercuri *et al.*, 2010 ▸; Sanvito, 2011*b* ▸; Yu *et al.*, 2011 ▸, 2012 ▸; Morita *et al.*, 2013 ▸; Murata *et al.*, 2013 ▸; Nakano, 2014 ▸; Chen *et al.*, 2016 ▸; Molčanov *et al.*, 2016 ▸; 2018*a* ▸), but neutral radicals tend to be better conductors, with conductivity reaching 10^−1^ S cm^−1^ (Itkis *et al.*, 2002 ▸; Podzorov, 2010 ▸; Mercuri *et al.*, 2010 ▸; Lekin *et al.*, 2010 ▸; Sanvito, 2011 ▸ *b*; Yu *et al.*, 2011 ▸, 2012 ▸; Morita *et al.*, 2013 ▸; Murata *et al.*, 2013 ▸; Nakano, 2014 ▸; Alemany *et al.*, 2015 ▸; Chen *et al.*, 2016 ▸).

The stability of a solid-state radical system depends mostly on the stability of the radical. Therefore, planar radicals with extensive delocalization of π-electrons are most widely used in crystal engineering, for example, derivatives of tetra­thia- and tetraselena­fulvalene (Ganesan *et al.*, 2003 ▸; Bendikov *et al.*, 2004 ▸; Rosokha & Kochi, 2007 ▸; Mercuri *et al.*, 2010 ▸; Morita *et al.*, 2013 ▸; Murata *et al.*, 2013 ▸), and semi­quinones (Rosokha & Kochi, 2007 ▸; Rosokha *et al.*, 2009 ▸; Molčanov *et al.*, 2016 ▸; 2018*a* ▸, 2019*b* ▸).

**Figure 1:**
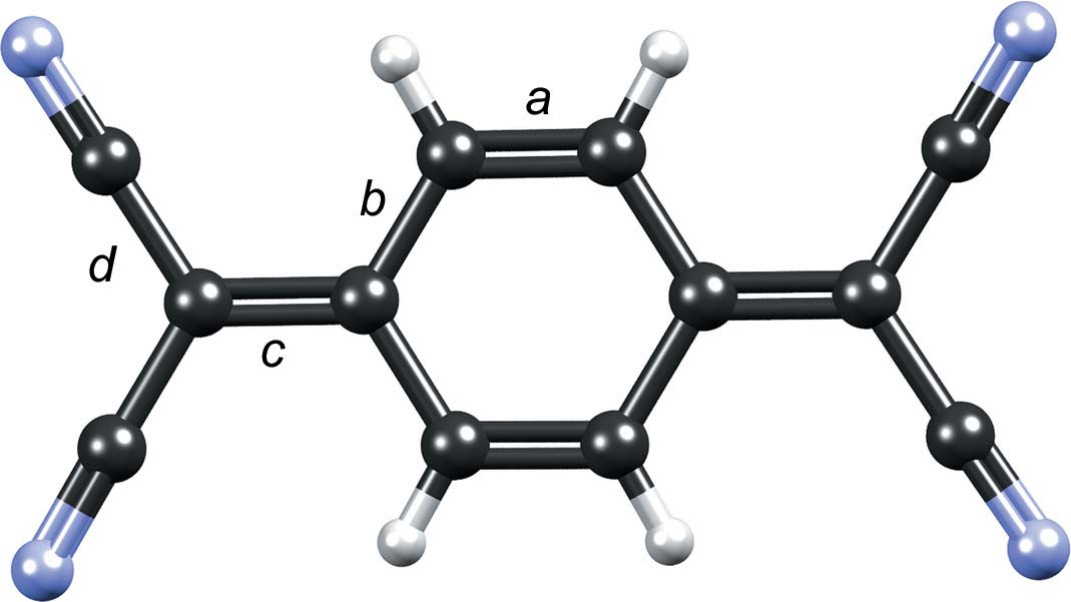
Lettering denotes the different types of C—C bonds in the TCNQ^δ−^ radical used for geometric correlation between the bond lengths and charge of the ring. With increasing negative charge and radical character, bonds *a* and *c* are elongated, whereas bonds *b* and *d* are shortened.

One of the most stable and extensively studied organic radicals is 7,7,8,8-tetra­cyano­quinodi­methane (TCNQ, neutral and radical forms are shown below), a strong electron [Chem scheme1] 
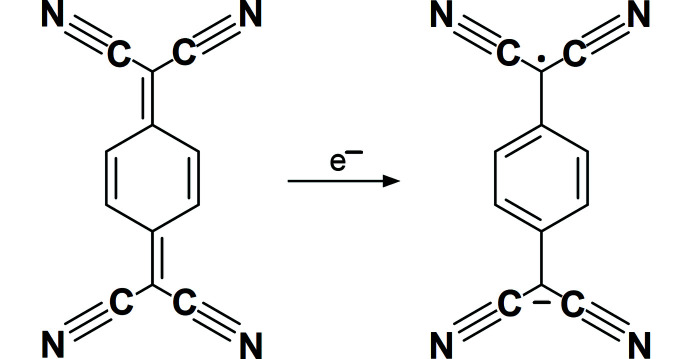
 acceptor suitable for the formation of salts. It comprises a six-membered ring similar to the quinones (Hünig, 1990 ▸) and is stabilized by four electron-withdrawing cyano groups. It also readily forms pancake bonds. However, unlike the semi­quinones, which usually form negatively charged radicals with a total charge of −1 (Molčanov *et al.*, 2012 ▸, 2016 ▸, 2018*a* ▸, 2019*a* ▸,*c* ▸; Molčanov & Kojić-Prodić, 2019 ▸), TCNQ is, in its salts, often only partially charged (its formal charge being −1/2 or −2/3), implying a partial radical character of the TCNQ^δ−^ moiety. Increasing negative charge enhances the delocalization of the π electrons; formally double bonds (*a* and *c* in Fig. 1 ▸) are elongated, whereas formally single bonds (*b* and *d* in Fig. 1 ▸) are shortened compared with the neutral compound. To estimate the charge of the TCNQ^δ−^ moiety, correlations of these bond lengths have been proposed, for example *c*/(*b*+*d*) (Kistenmacher *et al.*, 1982 ▸). In a review of a larger number of compounds containing TCNQ, Herbstein & Kapon (2008 ▸) used differences in bond lengths *b*–*a* and *c*–*d*. Their respective values for the neutral compound are 0.096 and −0.052 Å and for a negatively charged (−1) anion radical are 0.068 and −0.012 Å. However, owing to a variety of reasons, these are of limited reliability (see the Results and discussion[Sec sec2]).

To date, over 1000 crystal structures containing TCNQ in various oxidation states, ranging from 0 to −1, have been published (Groom *et al.*, 2016 ▸). A detailed review was published by Harms *et al.*(1982 ▸). Thus, a systematic analysis of crystal packing and molecular interactions is beyond the scope of this paper; here we discuss only structures with π-interactions between the TCNQ moieties, but not mixed stacks involving electron donors and acceptors, represented by a well known molecular complex of TCNQ with tetra­thia­fulvalene (TTF) (Schultz *et al.*, 1976 ▸).

Unlike semi­quinones, which usually form stacks of pancake-bonded dimers (or, more rarely, stacks of equidistant radicals) (Molčanov *et al.*, 2018*a* ▸; 2019*a* ▸,*b* ▸; Molčanov & Kojić-Prodić, 2019 ▸), TCNQ^δ−^ radicals form different types of pancake-bonded oligomers and a wide variety of long-range ordering. In many structures, isolated pancake-bonded dimers (which form no stacking interaction with neighbouring molecules) are present (Keller *et al.*, 1981 ▸; Harms *et al.*, 1982 ▸; Abashev *et al.*, 1987 ▸; Miller *et al.*, 1987 ▸; O’Hare *et al.*, 1990 ▸; Stein *et al.*, 1991 ▸; Grossel & Weston, 1992 ▸; Chen *et al.*, 2008 ▸; Sutton *et al.*, 2016 ▸; Park *et al.*, 2018 ▸). Sometimes the dimers form 1D stacks similar to those of semi­quinones (Hoekstra *et al.*, 1972 ▸; Ashwell *et al.*, 1975*a* ▸, 1977*a* ▸; Bosch & Bodegom, 1977 ▸; Endres *et al.*, 1978 ▸,*b* ▸; Diezt *et al.*, 1981 ▸; Konno & Saito, 1981 ▸; Waclawek *et al.*, 1983 ▸; Visser *et al.*, 1990*a* ▸,*b* ▸,*c* ▸,*d* ▸,*e* ▸; Magonov *et al.*, 1991 ▸; Ballester *et al.*, 1997 ▸; Moore *et al.*, 2001 ▸; Mukai *et al.*, 2001 ▸; Nishimura *et al.*, 2002 ▸; Chen *et al.*, 2008 ▸; Qu *et al.*, 2011 ▸; Martin *et al.*, 2012 ▸; Lu *et al.*, 2017 ▸; Üngör *et al.*, 2021*a* ▸), but also planar 2D arrays have been described (Ashwell *et al.*, 1982 ▸, 1983 ▸; Bryce *et al.*, 1988 ▸; Brook & Koch, 1997 ▸; Radváková *et al.*, 2010 ▸; Qu *et al.*, 2011 ▸); in some of these structures the formal charge (*i.e.* charge derived from stoichiometry) of TCNQ is −1/2 (Ashwell *et al.*, 1977*a* ▸, 1982 ▸). Pancake-bonded trimers, which often form stacks, are quite common, with a formal charge on the TCNQ^δ−^ moiety of −2/3 (Ashwell *et al.*, 1977*b* ▸; Endres *et al.*, 1978*a* ▸,*b* ▸; Ashwell & Wallwork, 1979 ▸; Sandman *et al.*, 1980 ▸; Lau *et al.*, 1982 ▸; Bell *et al.*, 1991 ▸; Usov *et al.*, 1991 ▸; Akutagawa *et al.*, 1996 ▸, 2004 ▸; Bigoli *et al.*, 1996 ▸; Ballester *et al.*, 2000 ▸; Nishijo *et al.*, 2004 ▸; Chen *et al.*, 2007 ▸; Liu *et al.*, 2008 ▸; Mochida *et al.*, 2008 ▸; Martin *et al.*, 2012 ▸; Kubota *et al.*, 2014 ▸; Phan *et al.*, 2015 ▸). However, different bond lengths in the central and lateral rings of a trimer indicate different total charge of the rings, which can be estimated using geometric correlations (see above). For example, in the compound (MT)_2_(TCNQ)_3_·2H_2_O (MT = *S*-methilthio­uronium) the charges on the central ring and the lateral rings were estimated to be −0.9 and −0.4, respectively; the total charge of a trimer is thus −1.7 (Usov *et al.*, 1991 ▸). In a 2:3 salt of *N,N*-di­methyl-d-proline-methyl­ester and TCNQ^δ−^ the rings form a trimer stack in the sequence ...*ABB*... with the charge of *A* being −0.30 and of *B* being −0.94 [thus, the charge of the trimer is −2.18 (Martin *et al.*, 2012 ▸)]. The trimers are usually centrosymmetric, and the central ring is usually more negatively charged than the lateral rings. In our previous studies we noted a similar distribution of charge in trimers of semi­quinone radicals (Molčanov *et al.*, 2018*b* ▸; 2019*b* ▸).

There are also tetramers, usually composed of TCNQ^δ−^ moieties with a formal charge of −1/2 [therefore, a tetramer has a charge of −2, implying paired spins (Ashwell & Nowell, 1984 ▸; Ashwell & Wallwork, 1985 ▸; Rindorf *et al.*, 1988 ▸; Takagi *et al.*, 1994 ▸; Malatesta *et al.*, 1995 ▸)]. These tetramers can stack, forming 1D (Ashwell *et al.*, 1977*c* ▸,*d* ▸; Filhol *et al.*, 1980 ▸; Filhol & Thomas, 1984 ▸; Üngör *et al.*, 2021*b* ▸) or 2D arrays (Kubota *et al.*, 2014 ▸). Stacks of equidistant radicals, which involve long-range antiferromagnetic ordering and semiconductive properties have also been described for a number of compounds (Shirotani & Kobayashi, 1973 ▸; Kistenmacher *et al.*, 1974 ▸; van Bodegom *et al.*, 1977 ▸; Endres, 1982 ▸; Bryce *et al.*, 1990 ▸; Visser *et al.*, 1990*f* ▸; Murata *et al.*, 2007 ▸; Chen *et al.*, 2008 ▸; Liu *et al.*, 2011 ▸). Less common are stacked pentamers (Ashwell *et al.*, 1977*c* ▸,*d* ▸, 1978 ▸; Lu *et al.*, 2011 ▸) and structures comprising two different types of stacks (Ashwell *et al.*, 1978 ▸; Murata *et al.*, 2006 ▸).

Studying the nature of multicentre covalent bonding between TCNQ^δ−^ radicals requires correlation of the crystal structures with magnetic and electric properties. Analogous to the detailed studies of stacked semi­quinones, it can be established with certainty that structures of pancake-bonded dimers and trimers with a total charge of −2 are diamagnetic and insulators, and that those of equidistant radicals are semiconductors with long-range magnetic order [most likely antiferromagnetic (Molčanov *et al.*, 2012 ▸, 2016 ▸, 2018*a* ▸,*b* ▸, 2019*c* ▸; Molčanov & Kojić-Prodić, 2019 ▸]. However, little can be said of the magnetic and electric properties of 2D arrays of dimers (TCNQ)_2_^−^ with a total charge of −1 (these contain a single unpaired electron) and tetramers of radicals, which do not have a well studied quinoid analogue. To determine properties of multicentre bonding in such systems, we prepared and characterized a series of fourteen novel salts of the TCNQ^δ−^ radical anion with planar organic cations.

## 2. Results and discussion

A series of salts of the partially charged TCNQ^δ−^ radical anion with the following organic cations was prepared: 2-bromo-*N*-methyl­pyridinium (**1**), 2-methyl-*N*-methyl­pyridinium (**2**), 3-iodo-*N*-methyl­pyridinium (**3**), 4-benzoyl-*N*-methyl­pyridinium (**4**), 4-di­methyl­amino*-N*-methyl­pyridinium (**5**), 4-iodo-*N*-methyl­pyridinium (**6**), 4-methyl-*N*-methyl­pyridinium (**7**), 3,5-di­bromo-*N*-ethyl­pyridinium (**8**), *N*,*N*′-di­ethyl-4,4′-bipyrid­inium (**9**), *N*,*N*′-di­methyl-4,4′-bipyridinium (**10**), 3-bromo-*N*-methyl­pyridinium (**11**), 3,5-di­bromo-*N*-methyl­pyridinium (**12**), *N*-methyl­pyridinium (**13**) and *N*-methyl-1,10-phenanthrolinium (**14**) (see below).[Chem scheme2] 
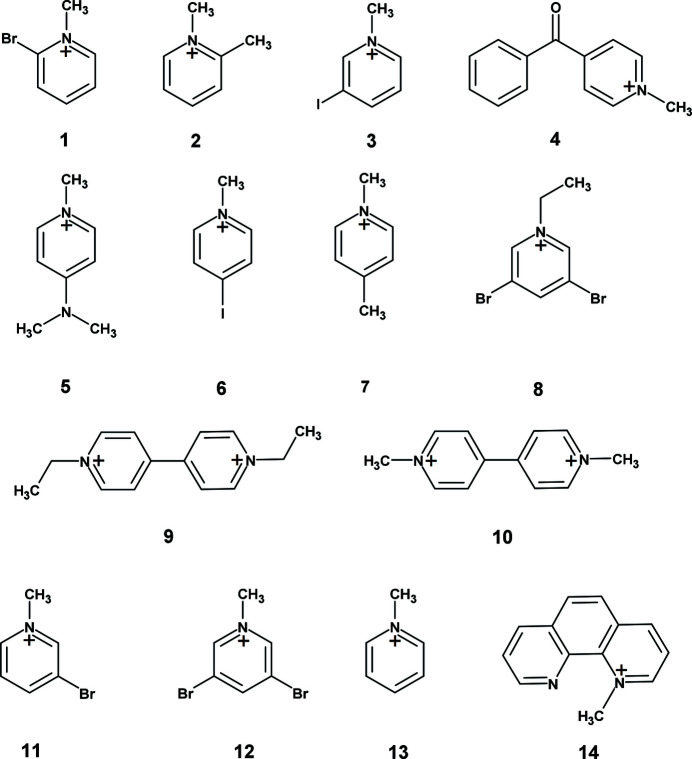


In the majority of the compounds there are two TCNQ^δ−^ moieties per one cation (or four in the case of the divalent *N*,*N*′-di­ethyl-4,4′-bipyridinium cation), so the stoichiometries are **2**·(TCNQ)_2_, **3**·(TCNQ)_2_, **4**·(TCNQ)_2_, **5**·(TCNQ)_2_, **6**·(TCNQ)_2_, **7**·(TCNQ)_2_, **8**·(TCNQ)_2_, **9**·(TCNQ)_4_, **12**·(TCNQ)_2_, **13**·(TCNQ)_2_ and **14**·(TCNQ)_2_. Two compounds include halide anions: **1**_2_·Br·(TCNQ)_2_ and **11**_2_·I·(TCNQ)_2_, and one crystallized with a stoichiometry of 1:3, **10**·(TCNQ)_3_.

### 2.1. Structure and charge of the TCNQ^δ−^ radical

Stoichiometries of the studied compounds indicate that the charge of TCNQ^δ−^ is −1/2, except in **10**·(TCNQ)_3_ where it is −2/3. Molecular geometry (Table S1 of the supporting information) and IR spectra (Table 1 ▸) are in agreement with the partial charge and partial radical character of the TCNQ^δ−^ moieties. It is known that with increasing negative charge and radical character, the molecular structure of TCNQ^δ−^ and related quinones change from quinoid towards semiquinoid: formally double bonds (*a* and *c* in Fig. 1 ▸) are elongated whereas formally single bonds (*b* and *d* in Fig. 1 ▸) are shortened. The geometric correlation by Kistenmacher *et al.* (1982 ▸) is 0.476 for neutral TCNQ, whereas for a negative radical with a total charge of −1 it is 0.500. For the salts studied in this work with a formal charge on TCNQ of −1/2 it is 0.487 (4), corresponding to a charge of 0.44 (17) (Table 2 ▸). Although the average value is close to the expected one, large variance makes this geometric correlation quite unreliable. Similar results are obtained if the correlation is expanded to (*a* + *c*)/(*b* + *d*). Average values of differences in bond lengths *b*–*a* and *c*–*d* for compounds with a formal charge on TCNQ of −1/2 studied in this work are 0.080 (10) and −0.034 (11) Å, respectively (Table 2 ▸). Again, the variances are too large to be reliable. Therefore, all these geometric correlations should be taken *cum grano salis*; as noted by Herbstein & Kapon (2008 ▸), ‘it would be haza­rdous to base a distinction (for a single determination) on bond lengths *alone*’. Our conclusions for related semi­quinone radicals were similar (Molčanov *et al.*, 2019*b* ▸). The reason for this variance of molecular geometry can be attributed to the effect of crystal field and especially the strong, partially covalent pancake bonding between the radicals.

In the case of **10**·(TCNQ)_3_, which comprises a pancake-bonded trimer with a formal charge of −2/3 [*i.e.* (TCNQ)_3_^2−^], the geometry indicates that the total charge of the central ring *B* is higher, −0.82, whereas the lateral rings *A* have a lower total charge of −0.45 (Table 2 ▸). Thus, the total charge of a trimer is −1.72, fairly close to the formal value of −2. Similar values have been found for similar trimers (Usov *et al.*, 1991 ▸). A more reliable assessment of total charge based on X-ray charge density was recently used in a study of a trimer of tetra­chloro­semi­quinone radicals with a formal charge of −2 (Molčanov *et al.*, 2018*b* ▸), and it revealed similarly uneven distribution of the negative charge: the central ring has a higher total charge of −0.76, whereas the two lateral rings have a charge of −0.59. The total charge of that trimer was −1.94 (Molčanov *et al.*, 2018*b* ▸). Accordingly, we may conclude that charge distribution in **10**·(TCNQ)_3_ is similar.

Assignment of IR spectra in salts of TCNQ^δ−^ radicals is not straightforward owing to the presence of cation vibrations, which often overlap with bands of the TCNQ^δ−^ radical. However, stretching of the cyano group does not overlap with other bands, so it can be used as a rough estimate for the ionization state of TCNQ^δ−^ (Herbstein & Kapon, 2008 ▸): in the neutral molecule it is 2228 cm^−1^, whereas in its mono anion there are two bands at 2194 and 2177 cm^−1^. In the studied salts, there are two bands at about 2200 and 2160−2170 cm^−1^ (Table 1 ▸), of which the first is blue-shifted and the second is red-shifted compared with the fully negatively charged radical anion, indicating a partial radical nature. The ethyl C=C stretching band is red-shifted compared with the neutral TCNQ^δ−^ by 20−25 cm^−1^, indicating weaker C=C bonds due to enhanced delocalization of π-electrons (Table 1 ▸).

### 2.2. Crystal packing and multicentre bonding

In all the studied crystal structures the dominant motif is the stacking of TCNQ^δ−^ radical anions, with a secondary motif of weak C—H⋯N hydrogen bonding between dimers (or oligomers) of the radicals. These two motifs create 2D-layered arrays of TCNQ^δ−^ radicals, which we discuss below. In all cases there are separate layers of radicals and cations, connected by C—H⋯N hydrogen bonds. Geometric parameters of π-stacking contacts are given in Table 3 ▸; note that interplanar distances corresponding to pancake bonding (*i.e.* those within the oligomers) are rather short (typically <3.25 Å), whereas those between the oligomers are longer (mostly exceeding 3.3 Å). However, these differences are much less pronounced than in semi­quinones, where inter-dimer distances usually exceed 3.4 Å (Molčanov *et al.*, 2016 ▸; 2018*a* ▸; 2019*b* ▸,*c* ▸).

A pancake-bonded dimer of partially charged TCNQ^δ−^ moieties, (TCNQ)_2_^−^, as a main unit is present in **1**_2_·Br·(TCNQ)_2_, **2**·(TCNQ)_2_, **3**·(TCNQ)_2_, **5**·(TCNQ)_2_, **6**·(TCNQ)_2_, **7**·(TCNQ)_2_, **8**·(TCNQ)_2,_ **11**_2_·I·(TCNQ)_2_, **12**·(TCNQ)_2__,_ **13**·(TCNQ)_2_ and **14**·(TCNQ)_2_. In **10**·(TCNQ)_3_ it is the trimer (TCNQ)_3_^2−^, and in **4**·(TCNQ)_2_ and **9**·(TCNQ)_4_ it is the tetramer (TCNQ)_4_^2−^. Dimers are common in almost all planar radicals, semi­quinones (Molčanov *et al.*, 2018*a* ▸; 2019*b* ▸,*c* ▸; Molčanov & Kojić-Prodić, 2019 ▸), various diazo­lyls (Lekin *et al.*, 2010 ▸; Yu *et al.*, 2011 ▸, 2012 ▸; Melen *et al.*, 2016 ▸; Nikolayenko *et al.*, 2017 ▸; Beldjoudi *et al.*, 2019 ▸) *etc*. They typically involve a pair of radicals, each having a single unpaired electron; thus the intermolecular HOMO is filled by a pair of electrons and the multicentre bond order is 1 (Mou & Kertesz, 2017 ▸; Molčanov *et al.*, 2018*b* ▸, 2019*a* ▸). Such structures are therefore diamagnetic. However, in the present case of TCNQ^δ−^, stoichiometry (and molecular geometry, see above) indicates that their charge is −1/2, so a dimer contains a single unpaired electron, (TCNQ)_2_^−^. Thus, the muticentre bond order is 1/2 (Mou & Kertesz, 2017 ▸; Molčanov *et al.*, 2018*b* ▸) and the single electron remains unpaired.

Trimers in **10**·(TCNQ)_3_ are similar to those observed in semi­quinones (Molčanov *et al.*, 2018*b* ▸, 2019*c* ▸; Molčanov & Kojić-Prodić, 2019 ▸), sharing two electrons per three radicals, implying a multicentre bond order of <0.71 (Molčanov *et al.*, 2018*b* ▸). The two electrons occupying the HOMO of the (TCNQ)_3_^2−^ group are paired and the ground state is antiferromagnetic.

Tetramers (TCNQ)_4_^2−^ in **4**·(TCNQ)_2_ and **9**·(TCNQ)_4_ involve four TCNQ^δ−^ radicals with a formal charge of −1/2, meaning two electrons occupy the HOMO. Therefore, the multicentre bond order is about 1/2 (Mou & Kertesz, 2017 ▸; Molčanov *et al.*, 2018*b* ▸), since a pair of electrons is shared by four TCNQ rings.

**Figure 2:**
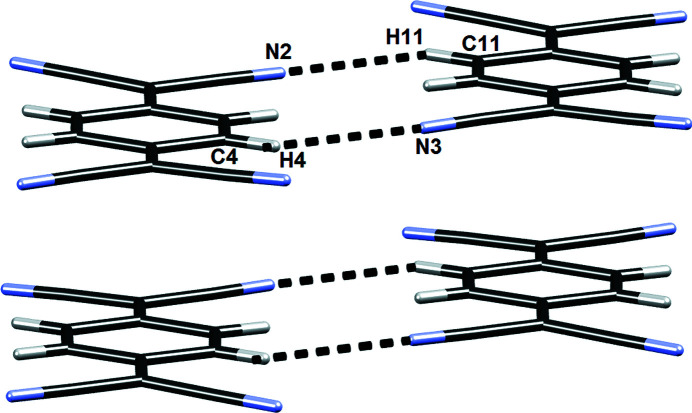
Typical lateral connection between pancake-bonded pairs in **1**_2_·Br·(TCNQ)_2_, involving two pairs of C—H⋯N hydrogen bonds.

**Figure 3:**
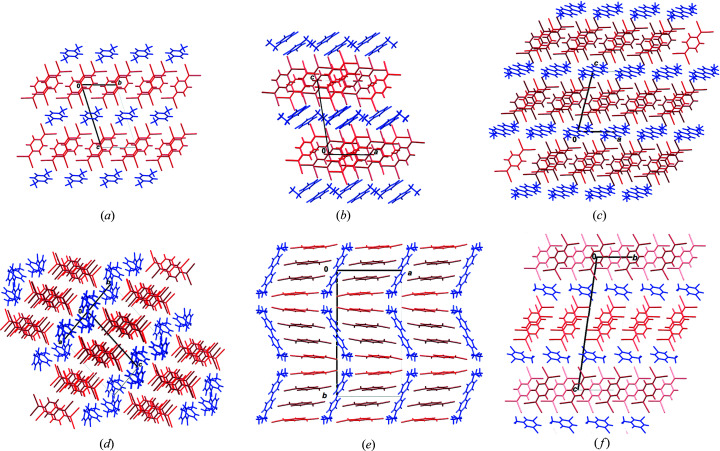
Crystal packings showing layers of TCNQ^δ−^ moieties (red) and cations (blue) of (*a*) **7**·(TCNQ)_2_, (*b*) **8**·(TCNQ)_2_, (*c*) **10**·(TCNQ)_3_, (*d*) **4**·(TCNQ)_2_, (*e*) **9**·(TCNQ)_4_ and (*f*) **12**·(TCNQ)_2_. Symmetry-independent moieties are shown in lighter and darker shades.

**Figure 4:**
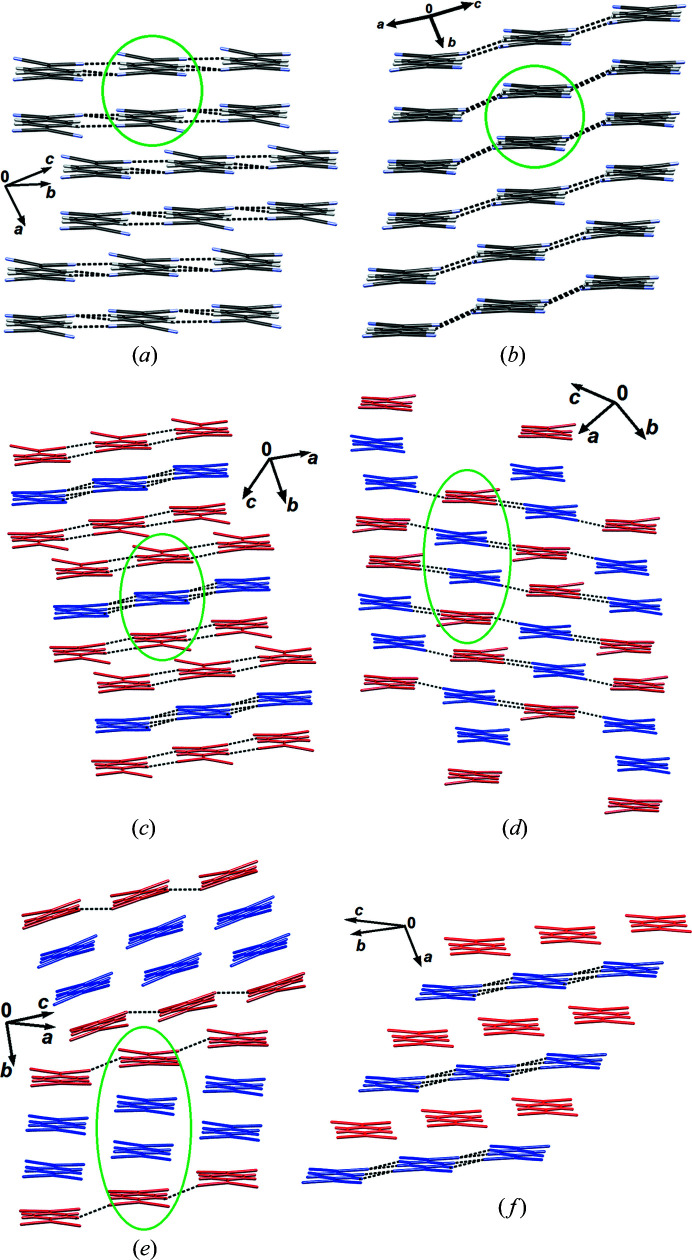
Different types of layers of TCNQ^δ−^ radicals observed in the compounds studied: (*a*) ‘brick wall’ array of pancake-bonded dimers in **7**·(TCNQ)_2_, (*b*) stacking of parallel hydrogen-bonded chains in **8**·(TCNQ)_2_, (*c*) brick-wall array of pancake-bonded trimers in **10**·(TCNQ)_3_ (*A* are red and *B* are blue), (*d*) brick wall array of pancake-bonded tetramers in **4**·(TCNQ)_2_ (*A* are red and *B* are blue), (*e*) herringbone-like array of pancake-bonded tetramers in **9**·(TCNQ)_4_ (*A* are red and *B* are blue) and (*f*) 2D array formed by stacks of equidistant radicals in **12**·(TCNQ)_2_ (*B* are red and *C* are blue). Individual dimers, trimers and tetramers are highlighted in green; hydrogen bonds are shown as black dotted lines.

The dimers, trimers and tetramers are stacked by somewhat weaker π-interactions (Molčanov & Kojić-Prodić, 2019 ▸; Molčanov *et al.*, 2019*c* ▸) and laterally connected by C—H⋯N hydrogen bonds (Table 4 ▸, Fig. 2 ▸) into 2D arrays. The cyano group of the TCNQ^δ−^ radical is a strong acceptor, and the proton-donating capability of the C−H group is barely affected by a partial negative charge. In addition, a lateral contact between two radicals involves two hydrogen bonds, so the interaction is fairly strong. Therefore, the motif shown in Fig. 2 ▸ is observed in all structures. All the studied structures involve alternating layers of anions and cations (Fig. 3 ▸), and these layers are connected by C−H⋯N hydrogen bonds with the cation acting as a weak donor and the cyano groups of TCNQ^δ−^ acting as acceptors (Fig. 4 ▸). Typically, there are only dispersion interactions between the cations. Stacking between aromatic cations was observed in the compounds **1**_2_·Br·(TCNQ)_2_, **4**·(TCNQ)_2_ and **11**_2_·I·(TCNQ)_2_, but it is a much weaker interaction with interplanar distances exceeding 3.6 Å. Such geometry is common for aromatic π-interactions (Molčanov & Kojić-Prodić, 2019 ▸; Molčanov *et al.*, 2019*c* ▸).

The most common type of 2D arrangement is a ‘brick wall’ pattern of pancake-bonded dimers, which is found in **1**_2_·Br·(TCNQ)_2_, **2**·(TCNQ)_2_, **3**·(TCNQ)_2_, **6**·(TCNQ)_2_, **7**·(TCNQ)_2_ 11_2_·I·(TCNQ)_2_, **12**·(TCNQ)_2_, **13**·(TCNQ)_2_ and **14**·(TCNQ)_2_ [Figs. 4 ▸(*a*) and S4–S17]. A similar pattern, although with fewer hydrogen bonds, is also present in **5**·(TCNQ)_2_. In **8**·(TCNQ)_2_ the pancake-bonded dimers form hydrogen-bonded chains parallel to [001], which are stacked on top of one another [Fig. 4 ▸(*b*)]. Brick-wall patterns are also formed by trimers in **10**·(TCNQ)_3_ [Fig. 4 ▸(*c*)] and tetramers in **4**·(TCNQ)_2_ [Fig. 4 ▸(*d*)]. Tetramers in **9**·(TCNQ)_4_ are laterally connected into chains parallel to [001], and are inclined by *ca* 20° towards the *b* axis. The contiguous chains are inclined in opposite directions, resulting in a herringbone-like pattern [Fig. 4 ▸(*e*)]. Interactions between the chains are very weak.

The unique crystal packing in the series studied is the structure of **12**·(TCNQ)_2_ which comprises three symmetry-independent TCNQ^δ−^ radicals (labelled *A*, *B* and *C*), each with a formal charge of −1/2. Molecule *A*, which is located in a general position, forms a 2D brick-wall pattern of pancake bonded dimers, identical to those described above [Fig. 4 ▸(*a*)]. Centrosymmetric anions *B* and *C* form equidistant stacks with alternating rings (...*BCBC*...) extending in the direction [100]. Hydrogen bonds (C−H⋯N) between the *C* rings connect the stacks into layers parallel to (001) [Fig. 4 ▸(*f*)]. The overall crystal packing exhibits alternating layers: anions *A*...cations...anions *B*/*C*...cations...anions *A*... (Fig. S15 of the supporting information).

**Figure 5:**
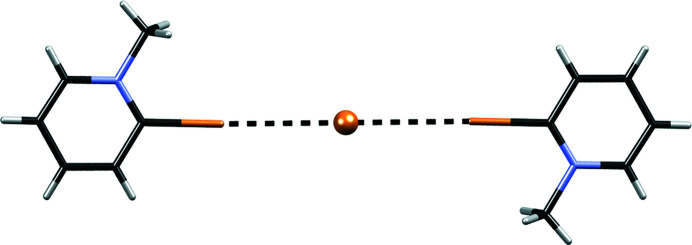
Bromide anion held by a pair of symmetry-related halogen bonds Br⋯Br in **1**_2_·Br·(TCNQ)_2_.

In some structures intermolecular halogen bonding is present. The crystal packing of **1**_2_·Br·(TCNQ)_2_ involves a bromide anion located at an inversion centre (p.p. 0.5) which is connected to two cations of **1** by a pair of symmetry-related Br⋯Br halogen bonds of 3.356 Å (Fig. 5 ▸). Similarly, in **11**_2_·I·(TCNQ)_2_, an iodide anion forms a pair of Br⋯I halogen bonds of 3.556 and 3.662 Å. In both structures the halogen bonds are (approximately) collinear [180° in **1**_2_·Br·(TCNQ)_2_ and at *ca* 161° in **11**_2_·I·(TCNQ)_2_]. In addition, the halide also acts as an acceptor of several C—H⋯Br (C—H⋯I) hydrogen bonding contacts, as is usually observed in halogeno­pyridine halogenides (Grebe *et al.*, 1999 ▸; Logothetis *et al.*, 2004 ▸; Caballero *et al.*, 2012 ▸; Fotović & Stilinović, 2020 ▸). Halogen bonding is also present in **3**·(TCNQ)_2_, **6**·(TCNQ)_2_, **8**·(TCNQ)_2_ and **12**·(TCNQ)_2_ where cyano groups of TCNQ^δ−^ anions act as halogen bond acceptors. However, there is a significant difference between the halogen bonds formed by the iodo­pyridinium cations [in **3**·(TCNQ)_2_ and **6**·(TCNQ)_2_] and the di­bromo­pyridinium cations [in **8**·(TCNQ)_2_ and **12**·(TCNQ)_2_]. The iodo­pyridinium cations form I⋯N halogen bonds [of 3.322 Å in **3**·(TCNQ)_2_ and 3.172 Å in **6**·(TCNQ)_2_] with the cyano nitro­gen lone pair, whereas the di­bromo­pyridinium cations form halogen bonds orthogonal to the cyano group (*i.e.* with the π-electrons of the cyano group). Note that both bond geometries have been observed in halogen-bonded structures of coordination complexes of cyanide ligands (Mínguez Espallargas *et al.*, 2009 ▸; Ormond-Prout *et al.*, 2012 ▸; Jakupec *et al.*, 2020 ▸) with a formal negative charge.

**Figure 6:**
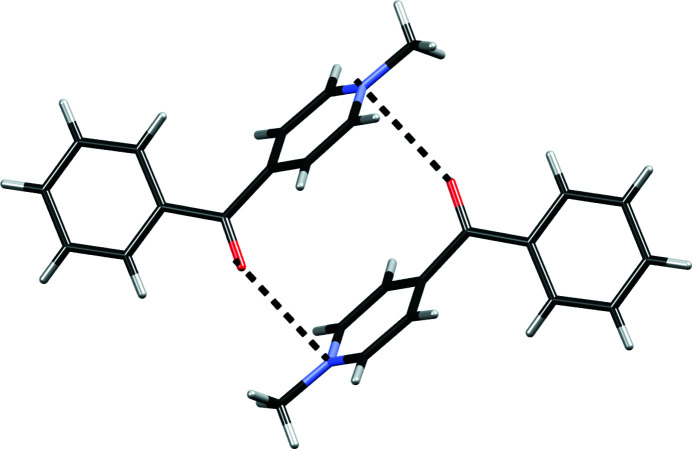
Dimer of cations of **4** in **4**·(TCNQ)_2_ formed by π–hole interactions (shown as dashed lines) and π-stacking of pyridyl rings enhanced by antiparallel local dipoles.

In **4**·(TCNQ)_2_ the cations form a dimer through a pair of π–hole interactions involving a carbonyl group and pyridinium ring; in addition the pyridinium rings stack, and this interaction is enhanced by antiparallel local dipoles (Fig. 6 ▸). π–Hole interactions are also present in **8**·(TCNQ)_2_, between a cyano group from the TCNQ^δ−^ molecule *B* and the pyridinium ring of the cation, and also in **12**·(TCNQ)_2_ between the iodide anion and pyridinium ring of the cation.

### 2.3. Correlation of electrical conductivity and crystal packing

**Figure 7:**
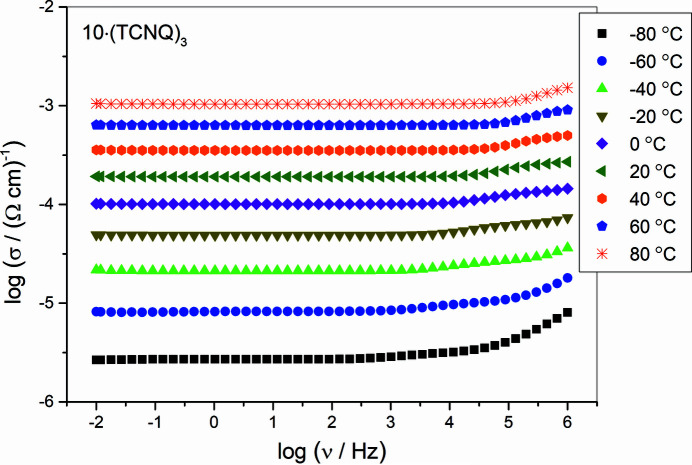
Electrical conductivity spectra of **10**·(TCNQ)_3_ at different temperatures.

Impedance spectroscopy measurements of selected compounds: **1**_2_·Br·(TCNQ)_2_, **4**·(TCNQ)_2_, **6**·(TCNQ)_2_, **8**·(TCNQ)_2_, **9**·(TCNQ)_4_, **10**·(TCNQ)_3_ and **12**·(TCNQ)_2_ showed that conductivity isotherms of all samples are frequency-independent (corresponding to DC conductivity) over a wide range of frequencies. Fig. 7 ▸ shows the conductivity spectra of **10**·(TCNQ)_3_ as typical spectra for all measured compounds. The observed behaviour is typical for electronically conducting materials and indicates fast electron transport (Austin, 1970 ▸; Morimoto *et al.*, 2019 ▸; Renka *et al.*, 2020 ▸).

**Figure 8:**
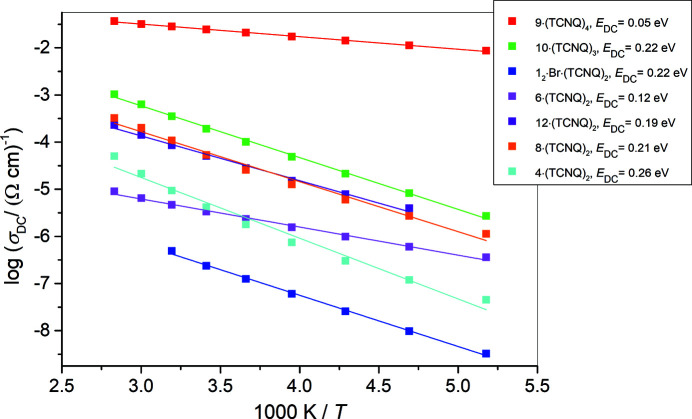
Electrical conductivity as a function of reciprocal temperature for all measured compounds. Solid lines represent the least-squares linear fit to the experimental data (full symbols).

For all measured compounds, the DC conductivity exhibits Arrhenius temperature dependence and hence has a characteristic activation energy, see Fig. 8 ▸. The activation energy for DC conductivity (*E*_DC_) for each compound was determined from the slope log(σ_DC_) versus 1000/*T* using the equation σ_DC_ *T* = σ_0_exp(−*E*_DC_/*k*_B_ *T*), where σ_DC_ is the conductivity, σ_0_ is the pre-exponential factor, *E*_DC_ is the activation energy, *k*_B_ is the Boltzmann constant and *T* is the temperature (K). The DC conductivities at 293 K and the determined activation energies (*E*_DC_) for all compounds are listed in Table 5 ▸.

The electrical conductivities are higher than those observed for similar salts of semi­quinones (Molčanov *et al.*, 2016 ▸; 2018*a* ▸), whose conductivities are below 10^−6^ (Ω cm^−1^) and are among the highest for ionic radicals. The phenomenon can be attributed to the shorter interplanar distance between oligomers (Table 3 ▸) and probably with a partial radical character of TCNQ^δ−^. The lowest-conducting compound **1**_2_·Br·(TCNQ)_2_ contains bromide anions as well as TCNQ^δ−^. The radical dimers are arranged in a 2D brick wall-like array (Fig. S4), but the interplanar distances between the dimers are only 0.05 and 0.10 Å longer than the distances within the dimer. These small differences imply that the electrons can jump between the dimers.

The next-lowest conducting compound is **4**·(TCNQ)_2_, which comprises a ‘brick wall’ pattern of tetramers; a relatively large inter-tetramer separation of 3.3732 (5) Å indicates a relatively high energy barrier for electrons jumping between the tetramers. The following four salts **6**·(TCNQ)_2_, **8**·(TCNQ)_2_, **9**·(TCNQ)_4_ and **10**·(TCNQ)_3_, and **12**·(TCNQ)_2_ possess different 2D packing motifs of dimers, trimers and tetramers (see above). In all of them the interplanar distances between the oligomers are shorter than 3.3 Å, which can explain conductivities of 10^−5^–10^−6^ (Ω cm)^−1^, as the energy barrier is lower than in the two previous compounds. In **12**·(TCNQ)_2_ there are two motifs, one with stacks of equidistant radicals which are known to be good conductors (Molčanov & Kojić-Prodić, 2019 ▸; Molčanov *et al.*, 2019*c* ▸). The other is a 2D ‘brick wall’ and is likely to be a very poor conductor since the interplanar distances between the dimers are >3.35 Å.

The highest conducting compound **9**·(TCNQ)_4_ reveals a herringbone-like array of pancake-bonded tetramers, where contiguous tetramers are not parallel, but inclined by 16.7° [Table 3 ▸, Fig. 4 ▸(*e*)]. Therefore, interplanar distances between tetramers cannot be defined; however, close contacts between them are indicated by perpendicular distances of only 2.81 Å. Such short contacts enable electron transfer throughout a herringbone-like layer, resulting in very high conductivity of 10^−2^ (Ω cm)^−1^, which can be compared to neutral radicals such as phenalenyls and di­thia­zolyls (Lekin *et al.*, 2010 ▸; Yu *et al.*, 2011 ▸, 2012 ▸; Morita *et al.*, 2013 ▸), and hybrid salts of TCNQ^δ−^ with Fe(II) complexes (Üngör *et al.*, 2021*b* ▸).

### 2.4. Electron paramagnetic resonance study

In the first step, the total spin concentrations were determined from the powder electron paramagnetic resonance (EPR) spectra of the samples at room temperature. The results are shown in Table 6 ▸. All powder samples have spin concentrations comparable to *N*_mol_ except for **8**·(TCNQ)_2_, whose spin concentration is 1% of *N*_mol_. From Table 6 ▸ it can be observed that the number of spins regarding the number of molecules differs from sample to sample. **6**·(TCNQ)_2_ and **12**·(TCNQ)_2_ have one electron spin per molecule, whereas **9**·(TCNQ)_4_ has two spins per molecule. These results agree very well with the charge calculations. **1**_2_·Br·(TCNQ)_2_ and **4**·(TCNQ)_2_ have about half an electron spin per single molecule. For the rest of the sample one can say the EPR signal is not a property of every molecule, specifically for **8**·(TCNQ)_2_. Regarding **4**·(TCNQ)_2_ and **10**·(TCNQ)_3_, we observed a magnetic transition that is not constant at room temperature and therefore the results show deviation from charge calculations. Further study at higher temperatures is needed to validate the agreement of *N*_spin_/*N*_mol_ with the calculated charges. On the other hand, the recorded single-crystal spectra of **8**·(TCNQ)_2_ show reasonable intensity, so the low powder spin concentration cannot be assigned to the defects of the structure. The temperature dependence of the EPR spectra studied in the temperature range 100–350 K for the powder samples of **1**_2_·Br·(TCNQ)_2_, **6**·(TCNQ)_2_, **9**·(TCNQ)_4_ and **12**·(TCNQ)_2_ shows that, by lowering the temperature, the EPR intensity increases (Figs. S19, S20 and S23), and the samples are paramagnetic in the range studied. In these cases of non-cooperative phenomena, electron spins associated with the TCNQ^δ−^ anions do not interact with each other (non-coupling spins), and the line intensities follow the Curie law which can be calculated using Equation (1),

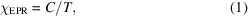

where *T* is the absolute temperature (in K), *C* is the material-specific Curie constant and χ_EPR_ is the magnetic susceptibility obtained by double integration of the EPR spectra.

**4**·(TCNQ)_2_ and **10**·(TCNQ)_3_ are paramagnetic at room temperature, but they do not follow the Curie law. Instead, EPR intensities show continuous decrease by lowering the temperature, indicating an antiferromagnetic transition.

For a deeper insight into the structure, orientation-dependent measurements of the single crystals at room temperature were performed and the changes of the *g*-value, intensity, linewidth and asymmetry were monitored. The *g*-factor is a second-rank tensor whose anisotropy arises from coupling of the spin angular momentum with the orbital angular momentum. The single crystal is rotated in the EPR cavity around a crystallographic axis and the measured *g*-value is a function of the orientation of the crystal with respect to the field. Presuming a molecular axis, *u*, *v* and *w* are the eigendirections of the *g*-tensor, the second rank tensor is given by (Wertz & Bolton, 1986 ▸)

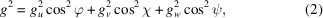

where φ, χ and ψ are the angles between the static magnetic field *B* and the *u*, *v* and *w* axes, respectively.

The EPR lines of the investigated samples **1**_2_·Br·(TCNQ)_2_, **4**·(TCNQ)_2_, **6**·(TCNQ)_2_, **9**·(TCNQ)_2_, **10**·(TCNQ)_3_ and **12**·(TCNQ)_2_ have a symmetric Lorentzian shape as a result of averaging out both the fine structure and the hyperfine splittings by spin carrier dynamics in the crystal lattices. Nevertheless, for some orientations of the crystals with respect to the static magnetic field, and for highly conducting large samples, an asymmetric line shape known as a Dysonian line was detected (Dyson, 1955 ▸). The physical origin of the Dysonian lineshape can be attributed to the fact that the incident microwave field only penetrates a conductive sample to a certain depth, the skin depth (δ), given by the relation

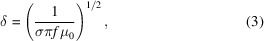

where σ is the electrical conductivity of the material, μ_0_ is the permeability of a vacuum (4π × 10^−7^ H m^−1^) and *f* is the microwave frequency.

Despite the averaging effects produced by dynamical spin delocalization and exchange and dipolar coupling, the *g*-factor and EPR linewidth of the **10**·(TCNQ)_3_ EPR spectra exhibited overly complicated spectra in the single-crystal study so no analysis was carried out. Looking at the complicated spectral shape of **10**·(TCNQ)_3_ (see Fig. S22), it seems justifiable that there are spectral components regarding a spin crossover, as expected for a triplet excited signal, but they are poorly resolved. It may be interpreted as a triplet with rather small zero-field splitting. Further investigations at high field or lower temperature are necessary.

Angular-dependent peak-to-peak linewidth (Γ) follows the general formula for dipolar broadening (Cheung & Soos, 1978 ▸),

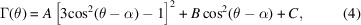

where *A*, *B* and *C* are temperature-dependent, experimentally determined parameters. The angle θ at θ = 0° marks the position with the largest linewidth, which occurs when the magnetic field is perpendicular to the plane.

Results obtained for **1**_2_·Br·(TCNQ)_2_, **4**·(TCNQ)_2_, **6**·(TCNQ)_2_, **9**·(TCNQ)_4_, **10**·(TCNQ)_3_ and **12**·(TCNQ)_2_ are shown in Fig. S24–S29 for all three crystal orientations (red, green, blue). Typically, variation of linewidth showing two peaks (one larger and one smaller, separated by approximately 90°) in two rotations about two axes suggest a 2D magnetic ordering and conductivity.

For **1**_2_·Br·(TCNQ)_2_, which has a 2D brick wall pattern of pancake-bonded dimers (Fig. S4) parallel to the plane (001), rotations around axes *a* and *b* show two maxima, 90° apart, with considerable variation of linewidth. This is consistent with strong interactions between spins and conductivity in two dimensions, parallel to the plane (001).

**4**·(TCNQ)_2_ exhibits the largest linewidth variations (0.05–0.3 mT) among all samples measured. The results support a 2D brick wall arrangement of pancake-bonded tetramers in the plane (001) and the strongest interaction in the direction [110] (*i.e.* direction of stacking). For **6**·(TCNQ)_2_ a single linewidth peak was observed in each rotation.

Crystals of **8**·(TCNQ)_2_ were mostly splinters, so it was difficult to establish habitus of the crystals and determine the main crystallographic directions. However, in one of the rotations (Fig. S27) two maxima of linewidth (one smaller, one larger) can be seen. This direction is likely to correspond to the strongest interactions (*i.e.* crystallographic *b* axis).

Since the crystals of **9**·(TCNQ)_4_ are monoclinic with β ≃ 93°, three orthogonal directions of rotation can be approximated with crystallographic axes *a*, *b* and *c*. Rotation about the *a* axis showed two maxima of linewidth, whereas the other two rotations had only one maximum.

**12**·(TCNQ)_2_ displayed rather narrow lines (<0.1 mT) at all orientations. However, variation of linewidths is in accordance with a 2D spin system: 2D arrangement (brick wall dimers and equidistant stacks) in the plane (001); stacking in two directions, one in the direction of the *a* axis, the other in the [110] direction (*i.e.* along the bis­ector of the *a* and *b* axes). The repeatable line asymmetries in rotations 1 and 3 of **12**·(TCNQ)_2_ have been observed which are unique among all samples studied. Taking into account that the conductivity is low, the asymmetry of other samples is more likely to be induced by imperfections. EPR parameters obtained by simulation are presented in Table 6 ▸.

In general, as can be seen from Table 7 ▸, the extracted *g*-values for the single-crystal measurements are in good agreement with those extracted from the powder samples. A degree of difference in the average *g*-value occurs due to the conductivity and difference in sample size regarding single-crystal and powder samples. The most unreliable parameter for all samples is the asymmetry as it depends on many different factors, see Equation (3). The parameters extracted from Equation (4) are shown in Table S3.

## 3. Conclusions

We prepared and structurally characterized 14 novel salts of the TCNQ^δ−^ radical anion with planar organic cations. All of them involve pancake-bonded oligomers (dimers, trimers or tetramers) arranged in extended 2D stacked arrays. Alternating anion and cation layers are held together by C—H⋯N hydrogen bonds. The most typical array is the brick wall pattern. Owing to the close contacts between the oligomers, the crystals are semiconductors with magnetic interactions and conductivity extending in the plane of stacking.

For a selected seven samples representing each type of stacking pattern (Fig. 4 ▸) we have determined the magnetic (EPR) and electric (impedance spectroscopy) properties and correlated them with crystal packing. Single-crystal EPR measurements confirmed that 2D stacked arrays of TCNQ^δ−^ radicals lead to long-range spin ordering and conductivity in two dimensions, corresponding to the plane of stacking. The most important factor for electrical conductivity is the distance between oligomers within a 2D array, as it facilitates electron transfer from one oligomer to another. Thus the electrical conductivity of **9**·(TCNQ)_4_ [2.44 × 10^−2^ (Ω cm)^−1^] is one of the highest among ionic radicals (Morita *et al.*, 2013 ▸; Murata *et al.*, 2013 ▸). This can be attributed the close contact between the pancake-bonded tetramers which are as short as 2.81 Å.

Though pancake bonding has been considered undesirable for the design of novel conductive materials, it is also energetically favourable and therefore difficult to avoid. However, the present work suggests to reverse the problem: 2D arrays with short distances between radical oligomers are both stable and good conductors. Therefore, this work offers a possible novel strategy for the design of organic conductors.

## 4. Experimental

### 4.1. Preparation and IR spectroscopy

All chemicals and solvents used were purchased from commercial sources (Merck, Alfa Aesar, Kemika), were of p.a. or reagent grade and used without additional purification.

The compounds were prepared by a modification of our method for the preparation of salts of semi­quinone radicals with organic cations (Molčanov *et al.*, 2011 ▸, 2012 ▸, 2016 ▸, 2018*a* ▸, 2019 ▸ *b* ▸). Powdered TCNQ (20.0 mg) was dissolved in cold acetone (5 ml, at 5°C) until the solution was approximately saturated. Into the solution an excess of solid iodide salt of the appropriate organic cation was added [in the case of **1**_2_·Br·(TCNQ)_2_ it was bromide]. The beaker was sealed with paraffin and left overnight at room temperature; the next day the acetone solution was decanted and black crystals of the TCNQ^δ−^ salt were washed with acetone and dried.

Infrared spectra were recorded with KBr pellets using a Bruker Alpha-T spectrometer in the 4000–350 cm^−1^ range.

### 4.2. X-ray diffraction

Single-crystal measurements were performed on an Oxford Diffraction Xcalibur Nova R (microfocus Cu tube) equipped with an Oxford Instruments CryoJet liquid nitro­gen cooling device. The *CrysAlisPRO* package (Rigaku OD, 2018 ▸) was used for data reduction and numerical absorption correction.

The structures were solved using *SHELXS97* (Sheldrick, 2015*a* ▸) and refined with *SHELXL-2017* (Sheldrick, 2015*b* ▸). Models were refined using the full-matrix least squares refinement; all non-hydrogen atoms were refined anisotropically. Hydrogen atoms were located in a difference Fourier map and refined either as riding entities or a mixture of free restrained and riding entities.

In the crystals of **2**·(TCNQ)_2_, **5**·(TCNQ)_2_, **7**·(TCNQ)_2_, **13**·(TCNQ)_2_ and **14**·(TCNQ)_2_ the cation is disordered about an inversion centre. Thus, two positions of the cation with a p.p. of 0.5 are present, as shown in Fig. S3. To better resolve the structures, the crystals were measured at 100 K; however, in some cases low-temperature data showed no improvement over room temperature. Interestingly, crystals of **13**·(TCNQ)_2_ at 100 K showed no disorder and the *b* axis doubled compared with the room-temperature structure. It is probable that two phases exist in the same sample, one with ordered and one with disordered cations, since a temperature-driven phase transformation is unlikely here (it would require rotation of an entire *N*-methyl­pyridinium cation by 180°).

Note that two salts with the iodine-substituted cation **3**·(TCNQ)_2_ and **6**·(TCNQ)_2_ crystallize in the rare non-centrosymmetric space group *P*1. The crystal packing of **3**·(TCNQ)_2_ obviously lacks any symmetry (other than translation), whereas **6**·(TCNQ)_2_ is pseudocentric with a pseudo-inversion centre coinciding with the centroid of the aromatic ring of the 4-iodo-*N*-methyl­pyridinium cation. Therefore, the structure can be solved in the space group *P* 1 with orientational disorder of the cation, similar to those described above. However, refinement of such a structure did not converge, and the disagreement factor *R* remained above 0.20. Refinement in the space group *P*1 proceeded smoothly; however, a small degree of orientational disorder of the cation is nevertheless present. This could be inferred from the unrealistically elongated N5—C18 bond [which was restrained to 1.40 (1) Å] and unusually small displacement ellipsoid of C18 (Fig. S2). This disorder was too small to be modelled.

Molecular geometry calculations were performed using *PLATON* (Spek, 2003 ▸; 2020 ▸) and molecular graphics were prepared with *ORTEP-3* (Farrugia, 1997 ▸) and *Mercury* (Macrae *et al.*, 2020 ▸). Crystallographic and refinement data for the structures reported in this paper are provided in Table 8 ▸. CCDC entries 2105173–2105191 contain the supplementary crystallographic data for this paper.

### 4.3. Electron paramagnetic resonance spectroscopy

EPR spectroscopy was used to determine the magnetic properties of the studied compounds. The single-crystal and powder measurements were carried out on a standard Bruker Elexsys 580 X-band EPR spectrometer, equipped with an Oxford continuous-flow cryostat with a flow of cold N_2_ gas, in the range 80–300 K. EPR spectra in the range 300–360 K were recorded on a Varian E-109 X-band spectrometer using a Bruker ER 4111VT variable-temperature unit with a flow of cold N_2_ gas. For the powder samples, Suprasil Quartz EPR Sample Tubes were used. The single crystal was evaluated first under a microscope and then by X-ray diffraction. It was aligned with a crystal axis on the EPR holder to ensure the EPR measurements were carried out on single crystals and to avoid twinning or multiple crystals that would affect our EPR measurements. The single crystals were mounted on the quartz rod with a goniometer and EPR spectra were recorded in three mutually perpendicular planes by rotating the crystals around the *a*, *b* and *c* axes at 5° intervals from 0 to 180°. The manganese standard reference Mn^2+^ in MgO was used to calibrate the magnetic field of the EPR spectrometer and consequently the *g*-value. The radical concentration in each sample was determined by dividing the value of the double integral of the EPR signal by the mass of the sample. Varian strong pitch was used as a standard to calculate the number of radicals. Spectral simulations were carried out using the *Easyspin* (Stoll & Schweiger, 2006 ▸) software working on the *MATLAB* platform (The Mathworks, 2011 ▸), and the in-house-made *Mathematica* program was used for the single-crystal spectra (Wolfram Research, 2019 ▸).

### 4.4. Electrical measurements

The electrical conductivities of **1**_2_·Br·(TCNQ)_2_, **4**·(TCNQ)_2_, **6**·(TCNQ)_2_, **8**·(TCNQ)_2_, **9**·(TCNQ)_4_, **10**·(TCNQ)_3_ and **12**·(TCNQ)_2_ were measured by impedance spectroscopy (Novocontrol Alpha-AN dielectric analyser) from 0.01 to 1 MHz at temperatures from −80 to 80°C (in steps of 20°C). The measurements were performed on polycrystalline samples pressed into pellets approximately 1 mm-thick. For the electrical contacts, gold electrodes (3.8 mm in diameter) were sputtered on the opposite surfaces of the pellets, except for the **4**·(TCNQ)_2_ sample, which was measured with Ag electrodes due to difficulties with Au deposition.

## Supplementary Material

Crystal structure: contains datablock(s) global, tcnq_2br_1, tcnq_2pic_100k_3, tcnq_2pic_2, tcnq_3i_1, tcnq_35bret, tcnq_44bpy_et_rt, tcnq_me2-44bpy_100k_2, tcnq_3brme_100k, tcnq_35brme, tcnq_nmepy_100k_2, tcnq_nmepy_2, tcnq_phen_100k, tcnq_phen_1, tcnq_4pic_100k, tcnq_4pic_1, tcnq_4bz_1, tcnq_4damp_2, tcnq_pi_3. DOI: 10.1107/S2052252522004717/lq5043sup1.cif

Supporting information file. DOI: 10.1107/S2052252522004717/lq5043sup2.pdf

Zipped structure-factor files. DOI: 10.1107/S2052252522004717/lq5043sup3.zip

CCDC references: 2105173, 2105174, 2105175, 2105176, 2105178, 2105179, 2105180, 2105181, 2105182, 2105183, 2105184, 2105185, 2105186, 2105187, 2105188, 2105189, 2105190, 2105191, 2105186

## Figures and Tables

**Table 1:** Selected absorption bands (cm^–1^) of TCNQ^δ−^ in the infrared spectra of compounds **1**–**14** Assignment according to Lunelli & Pecile (1970 ▸). Overlapping bands, which could not be well resolved, are italic; w – weak; sh – shoulder.

Compound	C—H stretch	C—N stretch	C=C ethyl stretch	C—H bend	C—CN stretch	NC—C—CN bend
**1**_2_·Br·(TCNQ)_2_	3053	2202, *2157*	1555, 1520	*1297*, 852	*1113*	482
**2**·(TCNQ)_2_	3057	2202, *2177*	1563, 1516	*1336*, 859sh	1114	480
**3**·(TCNQ)_2_	3047	2202, 2158	1555, 1513	*1341*sh, 837	*1115*	480
**4**·(TCNQ)_2_	3047	2196, *2160*	1557, 1522	1323, 859	*1115*	480
**5**·(TCNQ)_2_	3053	2200, *2171*sh	1563, 1515	*1330*, 822	*1113*	470
**6**·(TCNQ)_2_	3057	2202, *2158*	1553, 1515	*1332*sh, 860	*1113*	483
**7**·(TCNQ)_2_	3055	2202, *2167*	1557, 1517	*1332*, 856sh	*1114*	487
**8**·(TCNQ)_2_	3059, 3035	2223w	1549	1338, 874	1121w	474
**9**·(TCNQ)_4_	3062	2198, 2176	1561, 1526	1344, 841	1115	478
**10**·(TCNQ)_3_	3051	2190, 2169	1564, 1516	1353, 862	*1113*sh	474
**11**_2_·I·(TCNQ)_2_	3053	2202, *2171*	1555, 1503	*1347*sh, 851	1104	480
**12**·(TCNQ)_2_	3051	2200, *2178*	1553, 1520	*1295*, 859sh	*1113*	480
**13**·(TCNQ)_2_	3057	2202, *2169*	1557, 1518	*1334*, 852w	*1111*	481
**14**·(TCNQ)_2_	3057	2204, *2169*	1561, 1525	1359, 843	*1117*sh	482

**Table 2:** Estimated charge by geometric correlations Chemical bonds (labelled *a*, *b*, *c* and *d*) correspond to those in Fig. 1 ▸. RT – room temperature.

Ring	*b*–*a*	*c*–*d*	*c*/(*b* + *d*)	(*a* + *c*)/(*b* + *d*)	Est. charge†
**1**_2_·Br·(TCNQ)_2_	0.091	−0.044	0.483	0.988	0.31
**2**·(TCNQ)_2_, 100 K	0.103	−0.055	0.478	0.993	0.10
**2**·(TCNQ)_2_, RT	0.080	−0.031	0.488	0.989	0.48
**3**·(TCNQ)_2_, *A*	0.083	−0.050	0.483	0.988	0.27
**3**·(TCNQ)_2_, *B*	0.070	−0.024	0.490	0.989	0.59
**4**·(TCNQ)_2_, *A*	0.088	−0.037	0.485	0.988	0.39
**4**·(TCNQ)_2_, *B*	0.073	−0.018	0.492	0.986	0.68
**5**·(TCNQ)_2_	0.088	−0.038	0.485	0.990	0.37
**6**·(TCNQ)_2_, *A*	0.073	−0.029	0.488	0.992	0.50
**6**·(TCNQ)_2_, *B*	0.087	−0.032	0.487	0.989	0.45
**7**·(TCNQ)_2_, 100 K	0.085	−0.040	0.484	0.991	0.35
**7**·(TCNQ)_2_, RT	0.082	−0.033	0.487	0.990	0.44
**8**·(TCNQ)_2_, *A*	0.082	−0.030	0.488	0.987	0.50
**8**·(TCNQ)_2_, *B*	0.080	−0.032	0.487	0.989	0.47
**9**·(TCNQ)_4_, *A*	0.083	−0.036	0.486	0.990	0.41
**9**·(TCNQ)_4_, *B*	0.075	−0.023	0.491	0.987	0.60
**11**_2_·I·(TCNQ)_2_, *A*	0.072	−0.024	0.490	0.989	0.58
**11**_2_·I·(TCNQ)_2_, *B*	0.066	−0.027	0.489	0.991	0.55
**12**·(TCNQ)_2_, *A*	0.070	−0.028	0.489	0.989	0.55
**12**·(TCNQ)_2,_ *B*	0.053	−0.010	0.497	0.984	0.86
**12**·(TCNQ)_2_, *C*	0.088	−0.050	0.481	0.992	0.22
**13**·(TCNQ)_2_, 100 K, *A*	0.091	−0.054	0.480	0.991	0.17
**13**·(TCNQ)_2_, 100 K, *B*	0.093	−0.051	0.481	0.991	0.20
**13**·(TCNQ)_2_, RT	0.077	−0.027	0.489	0.989	0.54
**14**·(TCNQ)_2_, 100 K	0.078	−0.034	0.487	0.987	0.48
**14**·(TCNQ)_2_, RT	0.077	−0.027	0.488	0.991	0.52
**10**·(TCNQ)_3_, *A* ‡	0.084	−0.032	0.487	0.989	0.45
**10**·(TCNQ)_3_, *B* ‡	0.065	−0.008	0.496	0.986	0.82
Average	0.08 (1)	−0.03 (1)	0.487 (4)	0.989 (2)	0.4 (2)
TCNQ neutral§	0.096	−0.052	0.480	0.990	0
TCNQ^−^ §	0.068	−0.012	0.495	0.983	−1

† From *c*/(*b*+*d*).

‡ Data for the trimer were not used to calculate the average.

§ Data obtained from the work by Herbstein & Kapon (2008 ▸).

**Table 3:** Geometric parameters of π interactions

π⋯π	Cg†⋯Cg (Å)	α‡	β§	Cg⋯plane(Cg2) (Å)	Offset (Å)	Symmetry operations on Cg2
**1**_2_·Br·(TCNQ)_2_						
C3→C11⋯C3→C11	5.166 (4)	0.0 (3)	52.1	3.176 (3)	4.075	2 − *x*, −2 − *y*, 2 − *z*
C3→C11⋯C3→C11	4.974 (4)	0.0 (3)	48.1	3.324 (3)	3.701	2 − *x*, −1 − *y*, 2 − *z*
C3→C11⋯C3→C11	3.720 (4)	0.0 (3)	32.7	3.129 (3)	2.011	3 − *x*, −2 − *y*, 2 − *z*
N5→C17⋯C5→C17	4.906 (6)	0.0 (5)	41.0	3.700 (4)	3.222	3 − *x*, −1 − *y*, 1 − *z*
**2**·(TCNQ)_2_, 100 K						
C3→C11⋯C3→C11	3.680 (2)	0.00 (18)	32.9	3.090 (1)	1.998	1 − *x*, 1 − *y*, 1 − *z*
C3→C11⋯C3→C11	5.014 (3)	6.63 (18)	48.0	3.013 (1)	3.728	*x*, 1/2 − *y*, −1/2 + *z*
C3→C11⋯C3→C11	5.014 (3)	6.63 (18)	53.1	3.353 (1)	4.008	*x*, 1/2 − *y*, 1/2 + *z*
**2**·(TCNQ)_2_Q, RT						
C3→C11⋯C3→C11	3.719 (1)	0.02 (9)	32.5	3.1379 (8)	1.997	1 − *x*, 1 − *y*, 1 − *z*
C3→C11⋯C3→C11	5.071 (1)	7.01 (9)	47.6	3.0665 (8)	3.744	*x*, 1/2 − *y*, −1/2 + *z*
C3→C11⋯C3→C11	5.071 (1)	7.01 (9)	52.8	3.4207 (8)	4.039	*x*, 1/2 − y, 1/2 + *z*
**3**·(TCNQ)_2_						
C3A→C11A⋯C3B→C11B	3.759 (4)	1.3 (3)	32.5	3.157 (3)	2.022	*x*, *y*, 1 + *z*
C3A→C11A⋯C3B→C11B	5.341 (4)	1.3 (3)	53.2	3.265 (3)	4.278	1 + *x*, −1 + *y*, 1 + *z*
C3A→C11A⋯C3B→C11B	4.770 (4)	1.3 (3)	44.6	3.343 (3)	3.349	1 + *x*, *y*, 1 + *z*
C3B→C11B⋯C3A→C11A	4.770 (4)	1.3 (3)	45.5	3.397 (2)	3.402	−1 + *x*, *y*, −1 + *z*
C3B→C11B⋯C3A→C11A	5.340 (4)	1.3 (3)	52.3	3.197 (2)	4.226	−1 + *x*, 1 + *y*, −1 + *z*
C3B→C11B⋯C3A→C11A	3.759 (4)	1.3 (3)	32.9	3.168 (2)	2.041	*x*, *y*, −1 + *z*
**4**·(TCNQ)_2_, 100 K						
C3A→C11A⋯C3A→C11A	3.8501 (7)	0.00 (5)	28.8	3.3732 (5)	1.856	−*x*, 1 − *y*, 1 − *z*
C3A→C11A⋯C3B→C11B	3.7472 (7)	2.57 (5)	33.2	3.1824 (5)	2.050	*x*, *y*, *z*
C3B→C11B⋯C3A→C11A	3.7471 (7)	2.57 (5)	31.9	3.1367 (4)	1.978	*x*, *y*, *z*
C3B→C11B⋯C3B→C11B	3.3476 (6)	0.03 (5)	20.8	3.1292 (4)	1.189	1 − *x*, 2 − *y*, 1 − *z*
N5→C17⋯N5→C17	4.4886 (7)	0.00 (5)	31.8	3.8140 (4)	2.367	1 − *x*, 3 − *y*, 2 − *z*
N5→C17⋯C20→C25	5.2401 (7)	61.04 (6)	47.0	3.8846 (4)	−	−*x*, 3 − *y*, 2 − *z*
C20→C25⋯N5→C17	5.2401 (7)	61.04 (6)	42.2	3.5715 (5)	−	−*x*, 3 − *y*, 2 − *z*
C20→C25⋯C20→C25	5.1280 (8)	0.00 (7)	50.8	3.240 (6)	3.975	−*x*, 2 − *y*, 2 − *z*
**4**·(TCNQ)_2_, RT						
C3A→C11A⋯C3A→C11A	3.9170 (8)	0.00 (7)	29.5	3.4101 (6)	1.927	−*x*, 1 − *y*, 1 − *z*
C3A→C11A⋯C3B→C11B	3.8016 (8)	2.04 (6)	32.8	3.2338 (6)	2.058	*x*, *y*, *z*
C3B→C11B⋯C3A→C11A	3.8017 (8)	2.04 (6)	31.7	3.1968(5)	1.999	*x*, *y*, *z*
C3B→C11B⋯C3B→C11B	3.4907 (7)	0.03 (6)	22.5	3.2250 (5)	1.336	1 − *x*, 2 − *y*, 1 − *z*
N5→C17⋯N5→C17	4.5411 (8)	0.00 (7)	30.9	3.8977 (6)	2.330	1 − *x*, 3 − *y*, 2 − *z*
N5→C17⋯C20→C25	5.255 (1)	61.99 (9)	46.5	4.0163 (5)		−*x*, 3 − *y*, 2 − *z*
C20→C25⋯N5→C17	5.255 (1)	61.99 (9)	40.2	3.6197 (9)		−*x*, 3 − *y*, 2 − *z*
C20→C25⋯C20→C25	5.364 (1)	0.0 (1)	51.9	3.3067 (9)	4.224	−*x*, 2 − *y*, 2 − *z*
**5**·(TCNQ)_2_						
C3→C11⋯C3→C11	3.798 (1)	0.02 (9)	32.1	3.2167 (8)	2.020	1 − *x*, 1 − *y*, 2 − *z*
**6**·(TCNQ)_2_						
C3A→C11A⋯C3B→C11B	5.033 (4)	0.5 (3)	51.0	3.145 (3)	3.910	*x*, *y*, *z*
C3A→C11A⋯C3B→C11B	5.082 (4)	0.5 (3)	50.3	3.271 (3)	3.908	*x*, 1 + *y*, *z*
C3A→C11A⋯C3B→C11B	3.727 (4)	0.5 (3)	33.0	3.124 (3)	2.030	1 + *x*, *y*, *z*
C3B→C11B⋯C3A→C11A	3.726 (4)	0.5 (3)	33.1	3.125 (3)	2.032	−1 + *x*, *y*, *z*
C3B→C11B⋯C3A→C11A	5.082 (4)	0.5 (3)	49.9	3.249 (3)	3.889	*x*, −1 + *y*, *z*
C3B→C11B⋯C3A→C11A	5.034 (4)	0.5 (3)	51.3	3.169 (3)	3.930	*x*, *y*, *z*
**7**·(TCNQ)_2_, 100 K						
C3→C11⋯C3→C11	3.6872 (9)	0.03 (7)	33.2	3.0866 (6)	2.017	−*x*, 2 − *y*, −*z*
C3→C11⋯C3→C11	5.1357 (9)	0.03 (7)	52.2	3.1470 (6)	4.059	1 − *x*, 1 − *y*, −*z*
C3→C11⋯C3→C11	4.9180 (9)	0.03 (7)	49.9	3.1696 (6)	3.760	1 − *x*, 2 − *y*, −*z*
**7**·(TCNQ)_2_, RT						
C3→C11⋯C3→C11	3.7390 (9)	0.02 (7)	32.7	3.1455 (6)	2.021	−*x*, −*y*, 1 − *z*
C3→C11⋯C3→C11	5.0008 (9)	0.02 (7)	49.7	3.2335 (6)	3.815	1 − *x*, −*y*, 1−*z*
C3→C11⋯C3→C11	5.1723 (9)	0.02 (7)	51.3	3.2310 (6)	4.039	1 − *x*, 1 − *y*, 1 − *z*
**8**·(TCNQ)_2_						
C3A→C11A⋯C3A→C11A	3.771 (1)	0.(1)	24.9	3.4198 (9)	1.590	2 − *x*, 1 − *y*, 2 − *z*
C3A→C11A⋯C3B→C11B	3.685 (1)	1.6 (1)	32.5	3.1408 (9)	1.978	*x*, *y*, 1 + *z*
C3B→C11B⋯C3A→C11A	3.685 (1)	1.6 (1)	31.5	3.1089 (9)	1.927	*x*, *y*, −1 + *z*
C3B→C11B⋯C3B→C11B	3.718 (1)	0.0 (1)	28.0	3.2831 (9)	1.744	1 − *x*, −*y*, −*z*
**9**·(TCNQ)_2_						
C3A→C11A⋯C3A→C11A	5.1343 (7)	16.73 (6)	43.1	2.8125 (5)	3.509	*x*, 1/2 − *y*, 1/2 + *z*
C3A→C11A⋯C3B→C11B	3.7304 (7)	2.08 (6)	31.6	3.1874 (5)	1.956	*x*, *y*, *z*
C3B→C11B⋯C3A→C11A	3.7304 (7)	2.08 (6)	31.3	3.1765 (5)	1.938	*x*, *y*, *z*
C3B→C11B⋯C3B→C11B	3.7423 (7)	0.00 (6)	31.9	3.1785 (5)	1.975	1 − *x*, 1 − *y*, *−z*
**10**·(TCNQ)_3_						
C3A→C11A⋯C3A→C11A	4.907 (1)	0.03 (9)	47.0	3.3459 (7)	3.590	−*x*, 3 − *y*, 1 − *z*
C3A→C11A⋯C3A→C11A	5.181 (1)	0.03 (9)	54.6	3.0009 (7)	4.224	1 − *x*, 3 − *y*, 1 − *z*
C3A→C11A⋯C3B→C5B_a	3.646 (1)	1.23 (8)	33.9	3.0362 (7)	2.036	*x*, *y*, *z*
C3B→C5B_a⋯C3A→C11A	3.646 (1)	1.23 (8)	33.6	3.0251 (7)	2.019	*x*, *y*, *z*
**11**_2_·I·(TCNQ)_2_						
C3A→C11A⋯C3A→C11A	3.638 (2)	0.0 (2)	31.4	3.105 (1)	1.896	1 − *x*, 2 − *y*, 2 − *z*
C3A→C11A⋯C3B→C11B	5.255 (2)	7.2 (2)	59.2	3.173 (1)	4.515	*x*, *y*, *z*
C3A→C11A⋯C3B→C11B	4.592 (2)	7.2 (2)	41.5	3.104 (1)	3.043	1 + *x*, *y*, *z*
C3B→C11B⋯C3A→C11A	4.592 (2)	7.2 (2)	47.5	3.438 (1)	3.383	−1 + *x*, *y*, *z*
C3B→C11B⋯C3A→C11A	5.255 (2)	7.2 (2)	52.9	2.689 (1)	4.189	*x*, *y*, *z*
C3B→C11B⋯C3B→C11B	3.688 (2)	0.0 (2)	33.4	3.079 (1)	2.029	−*x*, 3 − *y*, 2 − *z*
N5→C17⋯N5→C17	4.622 (2)	0.0 (2)	36.3	3.725 (1)	2.736	−*x*, 2 − *y*, 1 − *z*
**12**·(TCNQ)_2_						
C3A→C11A⋯C3A→C11A	4.017 (2)	0.0 (1)	33.3	3.357 (1)	2.207	1 − *x*, 1 − *y*, 1 − *z*
C3A→C11A⋯C3A→C11A	3.694 (2)	0.0 (1)	33.0	3.098 (1)	2.012	2 − *x*, 1 − *y*, 1 − *z*
C3B→C10B_a⋯C3C→C10C_b	4.240 (2)	1.1 (1)	39.9	3.307 (1)	2.718	−1 + *x*, −1 + *y*, *z*
C3C→C10C_b⋯C3B→C10B_a	4.240 (2)	1.1 (1)	38.7	3.254 (1)	2.653	*x*, *y*, *z*
**13**·(TCNQ)_2_, 100 K						
C3A→C11A⋯C3A→C11A	3.689 (1)	0.02 (9)	33.0	3.0946 (8)	2.008	1 − *x*, 2 − *y*, 2 − *z*
C3A→C11A⋯C3B→C11B	5.064 (1)	8.23 (9)	47.3	2.9296 (8)	3.719	−*x*, 1 − *y*, 1 − *z*
C3A→C11A⋯C3B→C11B	4.935 (1)	8.23 (9)	53.9	3.3783 (8)	3.990	1 − *x*, 1 − *y*, 1 − *z*
C3B→C11B⋯C3A→C11A	5.064 (1)	8.23 (9)	54.7	3.4366 (8)	4.130	−*x*, 1 − *y*, 1 − *z*
C3B→C11B⋯C3A→C11A	4.935 (1)	8.23 (9)	46.8	2.9043 (8)	3.597	1 − *x*, 1 − *y*, 1 − *z*
C3B→C11B⋯C3B→C11B	3.689 (1)	0.02 (9)	33.1	3.0914 (8)	2.013	−*x*, 1 − *y*, −*z*
**13**·(TCNQ)_2_, RT						
C1→C6⋯C1→C6	3.723 (2)	0.0 (1)	32.5	3.139 (1)	2.002	−*x*, −*y*, 1 − *z*
C1→C6⋯C1→C6	5.017 (2)	0.0 (1)	49.2	3.277 (1)	3.798	1 − *x*, −*y*, 1 − *z*
C1→C6⋯C1→C6	5.082 (2)	0.0 (1)	51.2	3.186 (1)	3.959	1 − *x*, 1 − *y*, 1 − *z*
**14**·(TCNQ)_2_, 100 K						
C3→C11⋯C3→C11	4.771 (2)	0.0 (2)	46.3	3.297 (1)	3.449	−*x*, 1 − *y*, 1 − *z*
C3→C11⋯C3→C11	5.265 (2)	0.0 (2)	53.1	3.162 (1)	4.210	−*x*, 2 − *y*, 1 − *z*
C3→C11⋯C3→C11	3.687 (2)	0.0 (2)	32.9	3.097 (1)	2.000	1 − *x*, 1 − *y*, 1 − *z*
**14**·(TCNQ)_2_, RT						
C3→C11⋯C3→C11	4.742 (2)	0.0 (1)	44.4	3.390 (1)	3.316	−*x*, 1 − *y*, 1 − *z*
C3→C11⋯C3→C11	5.423 (2)	0.0 (1)	53.7	3.207 (1)	4.373	−*x*, 2 − *y*, 1 − *z*
C3→C11⋯C3→C11	3.738 (2)	0.0 (1)	32.4	3.155 (1)	2.004	1−*x*, 1 − *y*, 1 − *z*

† Cg – centroid of the aromatic ring.

‡ α – angle between planes of two interacting rings.

§ β – angle between Cg⋯Cg line and normal to the plane of the first interacting ring.

**Table 4:** Geometric parameters of hydrogen bonds

	*D*—H (Å)	H⋯*A* (Å)	*D*⋯*A* (Å)	*D*—H⋯*A* (°)	Symmetry operations on *A*
**1**_2_·Br·(TCNQ)_2_					
C16—H16⋯N4	0.93	2.48	3.39 (2)	167	*x*, 1 + *y*, *z*
C18—H18A⋯N4	0.96	2.59	3.12 (1)	115	1 + *x*, *y*, *z*
**2**·(TCNQ)_2_, 100 K					
C4—H4⋯N3	0.93	2.59	3.266 (7)	130	*x*, *y*, −1 + *z*
C11—H11⋯N2	0.93	2.57	3.248 (7)	130	*x*, *y*, 1 + *z*
C16—H16⋯N1	0.93	2.53	3.42 (1)	161	−1 + *x*, −1/2 − *y*, 1/2 + *z*
**3**·(TCNQ)_2_					
C4A—H4A⋯N3A	0.93	2.59	3.30 (1)	134	*x*, 1 + *y*, *z*
C4B—H4B⋯N3B	0.93	2.54	3.28 (1)	136	*x*, 1 + *y*, *z*
C11A—H11A⋯N2A	0.93	2.56	3.29 (1)	135	*x*, −1 + *y*, *z*
C11B—H11B⋯N2B	0.93	2.61	3.36 (1)	138	*x*, −1 + *y*, *z*
C13—H13⋯N1A	0.93	2.31	3.24 (1)	177	−1 + *x*, 1 + *y*, −1 + *z*
C15—H15⋯N4B	0.93	2.61	3.35 (2)	136	1 + *x*, *y*, 1 + *z*
C18—H18A⋯N4B	0.96	2.59	3.55 (2)	179	*x*, 1 + *y*, 1 + *z*
**4**·(TCNQ)_2_					
C5A—H5A⋯N2B	0.93	2.60	3.331 (2)	136	−*x*, 2 − *y*, 1 − *z*
C11A—H11A⋯N3B	0.93	2.48	3.282 (2)	145	1 − *x*, 1 − *y*, 1 − *z*
C14A—H14A⋯N1B	0.93	2.45	3.170 (2)	134	*x*, 1 + *y*, 1 + *z*
C16—H16⋯N2B	0.93	2.54	3.457 (2)	168	*x*, *y*, *z*
C18—H18C⋯N4A	0.96	2.49	3.305 (2)	143	*x*, 1 + *y*, *z*
C22—H22⋯N1B	0.93	2.52	3.374 (3)	153	−*x*, 2 − *y*, 1 − *z*
**6**·(TCNQ)_2_					
C13—H13⋯N4A	0.93	2.55	3.27 (1)	135	*x*, *y*, 1 + *z*
C16—H16⋯N1B	0.93	2.50	3.29 (1)	143	*x*, *y*, *z*
C17—H17⋯N1A	0.93	2.59	3.48 (2)	143	−1 + *x*, *y*, *z*
**7**·(TCNQ)_2_, 100 K					
C13—H13⋯N1	0.93	2.54	3.242 (2)	133	1 − *x*, 1 − *y*, 1 − *z*
**7**·(TCNQ)_2_, RT					
N6—H16A⋯N1	1.00 (4)	2.56 (4)	3.316 (4)	133 (3)	−1 + *x*, *y*, *z*
C13—H13⋯N1	0.93	2.56	3.302 (3)	137	1 − *x*, 1 − *y*, 2 − *z*
**8**·(TCNQ)_2_					
C11A—H11A⋯N1A	0.93	2.57	3.250 (3)	130	−1 + *x*, *y*, *z*
C13—H13⋯N2B	0.93	2.46	3.220 (3)	139	*x*, 1 + *y*, 1 + *z*
C17—H17⋯N3A	0.93	2.53	3.284 (3)	139	−1 + *x*, *y*, −1 + *z*
C18—H18B⋯N4B	0.97	2.62	3.366 (3)	134	−*x*, 1 − *y*, −*z*
**9**·(TCNQ)_4_					
C13—H13⋯N2A	0.93	2.43	3.353 (2)	172	1 − *x*, 1 − *y*, 1 − *z*
C1—H1⋯N2B	0.93	2.42	3.332 (2)	167	1 − *x*, 1 − *y*, 1 − *z*
C17—H17⋯N3B	0.93	2.40	3.239 (2)	150	−*x*, 1 − *y*, −*z*
**10**·(TCNQ)_3_					
C13—H13⋯N3A	0.93	2.54	3.447 (3)	165	−*x*, 2 − *y*, 2 − *z*
C14—H14⋯N3B	0.93	2.34	3.116 (2)	140	−1 + *x*, *y*, 1 + *z*
C17—H17⋯N2A	0.93	2.55	3.250 (2)	132	1 − *x*, 3 − *y*, 1 − *z*
**11**_2_·I·(TCNQ)_2_					
C5A—H5A⋯N4A	0.93	2.60	3.341 (4)	137	1 + *x*, *y*, *z*
C5B—H5B⋯N4B	0.93	2.51	3.267 (4)	139	1 + *x*, *y*, *z*
C10A—H10A⋯N1A	0.93	2.57	3.277 (4)	133	−1 + *x*, *y*, *z*
C10B—H10B⋯N1B	0.93	2.53	3.285 (4)	139	−1 + *x*, *y*, *z*
C13B—H13B⋯I1	0.93	2.95	3.848 (4)	164	2 − *x*, 1 − *y*, 1 − *z*
C15B—H15B⋯N2A	0.93	2.60	3.332 (6)	136	2 − *x*, 2 − *y*, 1 − *z*
C17A—H17A⋯N2A	0.93	2.59	3.495 (5)	164	−1 + *x*, *y*, *z*
C17B—H17B⋯N2B	0.93	2.54	3.225 (5)	131	2 − *x*, 2 − *y*, 1 − *z*
C18B—H18B⋯I1	0.96	3.05	3.784 (4)	134	−1 + *x*, *y*, *z*
C18B—H18D⋯N3B	0.96	2.54	3.484 (5)	166	2 + *x*, −1 + *y*, −1 + *z*
**12**·(TCNQ)_2_					
C4C—H4C⋯N1C	0.93	2.62	3.244 (3)	125	2 − *x*, −*y*, −*z*
C13—H13⋯N3C	0.93	2.51	3.375 (4)	155	*x*, −1 + *y*, *z*
**13**·(TCNQ)_2_, 100 K					
C4A—H4A⋯N3A	0.93	2.62	3.318 (3)	132	1 + *x*, *y*, *z*
C4B—H4B⋯N3B	0.93	2.60	3.272 (3)	130	1 + *x*, *y*, *z*
C11B—H11B⋯N2B	0.93	2.60	3.271 (3)	129	−1 + *x*, *y*, *z*
C13—H13⋯N1B	0.93	2.44	3.356 (3)	170	*x*, *y*, *z*
C14—H14⋯N4B	0.93	2.49	3.316 (3)	148	−*x*, 1 − *y*, −*z*
C15—H15⋯N4B	0.93	2.56	3.305 (4)	137	1 + *x*, 1 + *y*, 1 + *z*
C16—H16⋯N1A	0.93	2.60	3.362 (4)	139	2 − *x*, 2 − *y*, 2 − *z*
C17—H17⋯N4A	0.93	2.61	3.446 (3)	150	1 + *x*, *y*, *z*
**14**·(TCNQ)_2_, 100 K					
C5—H5⋯N4	0.93	2.56	3.270 (5)	134	*x*, 1 + *y*, *z*
C10—H10⋯N1	0.93	2.60	3.317 (5)	134	*x*, −1 + *y*, *z*
C15—H15⋯N3	0.93	2.60	3.338 (6)	137	1 + *x*, *y*, *z*
**14**·(TCNQ)_2_, RT					
C5—H5⋯N4	0.93	2.59	3.299 (4)	134	*x*, 1 + *y*, *z*

**Table 5:** Electrical conductivity (σ_DC_) and activation energy for conductivity (*E*_DC_) of selected compounds

Compound	σ_DC_ (Ω cm) ^−1^ at 20°C		*E*_DC_ (eV)
**1**_2_·Br·(TCNQ)_2_	2.36 × 10^−7^		0.22
**4**·(TCNQ)_2_	4.15 × 10^−6^		0.26
**6**·(TCNQ)_2_	3.35 × 10^−6^		0.12
**8**·(TCNQ)_2_	5.29 × 10^−5^		0.21
**9**·(TCNQ)_4_	2.44 × 10^−2^		0.05
**10**·(TCNQ)_3_	1.91 × 10^−4^		0.22
**12**·(TCNQ)_2_	5.07 × 10^−5^		0.19

**Table 6:** Spin counting results

Sample	*M*_r_	*N*_mol_	*N*_spin_	*N*_spin_/*N*_mol_	Formal charge
**1**_2_·Br·(TCNQ)_2_	461.15	5.2584e+18	2.4524e+18	0.46±0.09	1
**4**·(TCNQ)_2_	606.61	1.8107e+18	5.6066e+17	0.31±0.06	1
**6**·(TCNQ)_2_	746.39	2.3884e+18	2.2957e+18	0.96±0.19	1
**8**·(TCNQ)_2_	746.39	2.3884e+18	9.6413e+14	0.0004±0.00008	1
**9**·(TCNQ)_4_	662.68	1.0416e+18	1.9589e+18	1.88±0.38	2
**10**·(TCNQ)_3_	594.63	1.2618e+18	7.2700e+16	0.06±0.01	2
**12**·(TCNQ)_2_	660.30	1.1908e+18	1.0446e+18	0.87±0.18	1

**Table 7:** Summary of the effective *g*-factor global fitting results for single-crystal and powder samples

Sample	{*g*_1_, *g*_2_, *g*_3_} (*g*_1_ ≥ *g*_2 _ ≥ *g*_3_)	*g*_average_	Eigenvectors
**1**_2_·Br·(TCNQ)_2_	{2.0039, 2.0031, 20.0028}	2.0032	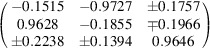
**4**·(TCNQ)_2_	{2.0039, 2.0032, 2.0027}	2.0033	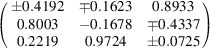
**6**·(TCNQ)_2_	{2.0040, 2.0035, 2.0026}	2.0034	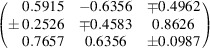
**8**·(TCNQ)_2_	{2.0041, 2.0031, 2.0028}	2.0033	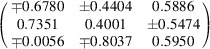
**9**·(TCNQ)_4_	{2.0039, 2.0032, 2.0028}	2.0033	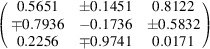
**12**·(TCNQ)_2_	{2.0035, 2.0032, 2.0027}	2.0031	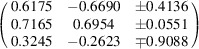

**Table:** 

Compound	**1**_2_·(TCNQ)_2_·Br	**2**·(TCNQ)_2_ 100 K	**2**·(TCNQ)_2_ RT	**3**·(TCNQ)_2_
Empirical formula	C_18_H_11_Br_1.50_N_5_	C_31_H_10_N_9_	C_31_H_10_N_9_	C_30_H_15_IN_9_
Formula weight (g mol^−1^)	417.18	508.48	508.48	628.41
Colour	Black	Black	Black	Black
Crystal dimensions (mm)	0.18 × 0.06 × 0.04	0.25 × 0.13 × 0.10	0.25 × 0.13 × 0.10	0.12 × 0.10 × 0.05
Space group	*P* 1	*P*2_1_/*c*	*P*2_1_/*c*	*P*1
*a* (Å)	7.2378 (4)	13.2712 (6)	13.3766 (2)	7.1680 (4)
*b* (Å)	7.7475 (4)	12.5807 (5)	12.8023 (2)	7.6291 (4)
*c* (Å)	17.8746 (12)	7.7388 (5)	7.78610 (10)	13.6797 (8)
α (°)	83.578 (5)	90	90	78.422 (5)
β (°)	83.241 (5)	90.466 (4)	90.3960 (10)	83.441 (5)
γ (°)	67.104 (5)	90	90	69.680 (5)
*Z*	2	2	2	1
*V* (Å^3^)	914.53 (10)	1292.04 (11)	1333.35 (3)	686.44 (7)
*D*_calc_ (g cm^−3^)	1.515	1.266	1.266	1.518
λ (Å)	1.54184 (Cu *K*α)	1.54184 (Cu *K*α)	1.54184 (Cu *K*α)	1.54184 (Cu *K*α)
μ (mm^−1^)	4.415	0.653	0.653	9.464
*Θ* range (°)	4.99–73.47	4.84–75.70	4.75–75.91	3.30–75.99
*T* (K)	293 (2)	100 (2)	293 (2)	293 (2)
Diffractometer type	Xcalibur Nova	Xcalibur Nova	Xcalibur Nova	Xcalibur Nova
Range of *h* *k* *l*	−9 < *h* < 8; −9 < *k* < 8; −19 < *l* < 22	−15 < *h* < 16; −15 < *k* < 14; −9 < *l* < 9	−16 < *h* < 13; −15 < *k* < 13; −9 < *l* < 9	−7 < *h* < 8; −7 < *k* < 9; −15 < *l* < 17
Reflections collected	7195	5148	7175	5556
Independent reflections	3677	2569	2655	3390
Observed reflections (*I* ≥ 2σ)	3245	1751	2368	3380
Absorption correction	Multi-scan	Multi-scan	Multi-scan	Multi-scan
*T*_min_, *T*_max_	0.2258, 1.0000	0.4314, 1.0000	0.7402, 1.0000	0.5928, 1.0000
*R*_int_	0.0267	0.0797	0.0155	0.0367
*R*(*F*)	0.0659	0.1360	0.0801	0.0421
*R*_w_(*F* ^2^)	0.2150	0.3914	0.2436	0.1145
Goodness of fit	1.055	1.064	1.063	1.032
H atom treatment	Constrained	Constrained	Constrained	Constrained
No. of parameters	224	190	190	361
No. of restraints	0	58	59	3
Δρ_max_, Δρ_min_, Δρ_r.m.s._ (eÅ^–3^)	1.054, −0.608, 0.127	1.088, −0.690, 0.132	0.495, −0.466, 0.057	0.536, −1.182, 0.079

**Table:** 

Compound	**4**·(TCNQ)_2_	**5**·(TCNQ)_2_	**6**·(TCNQ)_2_	**7**·(TCNQ)_2_ 100 K
Empirical formula	C_37_H_20_N_9_O	C_16_H_8_N_5_	C_30_H_15_IN_9_	C_15_H_9_N_5_
Formula weight (g mol^−1^)	606.62	270.27	628.41	259.27
Colour	Black	Black	Black	Black
Crystal dimensions (mm)	0.40 × 0.15 × 0.12	0.22 × 0.20 × 0.03	0.15 × 0.09 × 0.05	0.38 × 0.32 × 0.05
Space group	*P* 1	*P* 1	*P*1	*P* 1
*a* (Å)	9.1474 (2)	7.5381 (5)	7.0735 (5)	7.0634 (4)
*b* (Å)	11.3666 (5)	8.0130 (7)	7.8171 (4)	7.8154 (5)
*c* (Å)	15.9321 (6)	13.3368 (12)	13.8419 (4)	13.4680 (8)
α (°)	106.234 (4)	103.618 (8)	74.353 (4)	73.628 (5)
β (°)	91.277 (3)	90.811 (6)	88.928 (4)	89.550 (5)
γ (°)	99.804 (3)	115.727 (8)	67.934 (6)	67.258 (6)
*Z*	2	2	1	2
*V* (Å^3^)	1562.98 (10)	699.07 (11)	680.10 (7)	653.56 (7)
*D*_calc_ (g cm^−3^)	1.289	1.267	1.534	1.317
λ (Å)	1.54184 (Cu *K*α)	1.54184 (Cu *K*α)	1.54184 (Cu *K*α)	1.54184 (Cu *K*α)
μ (mm^−1^)	0.665	0.648	9.552	0.679
*Θ* range (°)	4.11–75.96	3.44–76.34	6.28–75.70	3.42–75.92
*T* (K)	293 (2)	293 (2)	293 (2)	100 (2)
Diffractometer type	Xcalibur Nova	Xcalibur Nova	Xcalibur Nova	Xcalibur Nova
Range of *h* *k* *l*	−7 < *h* < 11; −13 < *k* < 14; −18 < *l* < 20	−9 < *h* < 8; −9 < *k* < 9; −16 < *l* < 15	−8 < *h* < 8; −9 < *k* < 9; −11 < *l* < 17	−7 < *h* < 8; −6 < *k* < 9; −14 < *l* < 16
Reflections collected	13967	4489	5812	4851
Independent reflections	6405	2612	3377	2657
Observed reflections (*I* ≥ 2σ)	5339	2083	3335	2343
Absorption correction	Multi-scan	Multi-scan	Multi-scan	Multi-scan
*T*_min_, *T*_max_	0.6377, 1.0000	0.5524, 1.0000	0.4396, 1.0000	0.6578, 1.0000
*R*_int_	0.0226	0.0320	0.0317	0.0455
*R*(*F*)	0.0458	0.0725	0.0625	0.0592
*R*_w_(*F* ^2^)	0.1796	0.2367	0.1460	0.1813
Goodness of fit	0.712	1.066	1.099	0.996
H atom treatment	Constrained	Constrained	Constrained	Constrained
No. of parameters	424	217	362	190
No. of restraints	0	0	5	6
Δρ_max_, Δρ_min_, Δρ_r.m.s._ (eÅ^–3^)	0.161, −0.146, 0.030	0.359, −0.235, 0.061	2.770, −0.802, 0.143	0.332, −0.313, 0.065

**Table:** 

Compound	**7**·(TCNQ)_2_ RT	**8**·(TCNQ)_2_	**9**·(TCNQ)_4_	**10**·(TCNQ)_3_
Empirical formula	C_15_H_9_N_5_	C_31_H_16_Br_2_N_9_	C_31_H_17_N_9_	C_48_H_26_N_14_
Formula weight (g mol^−1^)	259.27	674.35	515.53	798.83
Colour	Black	Black	Black	Black
Crystal dimensions (mm)	0.30 × 0.28 × 0.05	0.41 × 0.28 × 0.04	0.20 × 0.18 × 0.08	0.31 × 0.12 × 0.10
Space group	*P* 1	*P* 1	*P*2_1_/*c*	*P* 1
*a* (Å)	7.1924(3)	8.0361 (3)	13.0864 (2)	7.8212 (5)
*b* (Å)	7.8489 (5)	13.0398 (3)	25.3377 (4)	9.7542 (7)
*c* (Å)	13.6170 (9)	14.2071(4)	7.84030 (10)	13.2653 (7)
α (°)	106.174 (6)	92.624 (2)	90	78.103 (5)
β (°)	90.739 (4)	98.576 (2)	92.6510 (10)	75.829 (5)
γ (°)	112.525 (5)	104.767 (3)	90	82.246 (5)
*Z*	2	2	4	1
*V* (Å^3^)	675.74 (7)	1418.00 (7)	2596.90 (7)	956.32 (11)
*D*_calc_ (g cm^−3^)	1.274	1.579	1.319	1.387
λ (Å)	1.54184 (Cu *K*α)	1.54184 (Cu *K*α)	1.54184 (Cu *K*α)	1.54184 (Cu *K*α)
μ (mm^−1^)	0.657	3.943	0.671	0.706
*Θ* range (°)	6.27–75.85	3.15–75.81	3.77–75.75	3.49–76.47
*T* (K)	293 (2)	293 (2)	293 (2)	100 (2)
Diffractometer type	Xcalibur Nova	Xcalibur Nova	Xcalibur Nova	Xcalibur Nova
Range of *h* *k* *l*	−8 < *h* < 5; −9 < *k* < 9; −16 < *l* < 16	−10 < *h* < 10; −16 < *k* < 15; −14 < *l* < 17	−15 < *h* < 16; −25 < *k* < 31; −9 < *l* < 9	−9 < *h* < 9; −12 < *k* < 12; −16 < *l* < 13
Reflections collected	5385	12861	13505	8051
Independent reflections	2719	5835	5368	3944
Observed reflections (*I* ≥ 2σ)	2333	5366	4600	3294
Absorption correction	Multi-scan	Multi-scan	Multi-scan	Multi-scan
*T*_min_, *T*_max_	0.4767, 1.0000	0.5426, 1.0000	0.6352, 1.0000	0.6772, 1.0000
*R*_int_	0.0279	0.0451	0.0180	0.0484
*R*(*F*)	0.0589	0.0416	0.0468	0.0686
*R*_w_(*F* ^2^)	0.1930	0.1124	0.1416	0.2069
Goodness of fit	1.018	0.778	0.929	0.928
H atom treatment	Constrained	Constrained	Constrained	Constrained
No. of parameters	190	379	371	280
No. of restraints	6	0	2	0
Δρ_max_, Δρ_min_, Δρ_r.m.s._ (eÅ^–3^)	0.231, −0.287, 0.044	0.836, −1.020, 0.095	0.257, −0.252, 0.032	0.465, −0.326, 0.083

**Table:** 

Compound	**11**_2_·I·(TCNQ)_2_	**12**·(TCNQ)_2_	**13**·(TCNQ)_2_ 100 K	**13**·(TCNQ)_2_ RT
Empirical formula	C_36_H_22_Br_2_IN_10_	C_30_H_14_Br_2_N_9_	C_30_H_16_N_9_	C_30_H_16_N_9_
Formula weight (g mol^−1^)	881.35	660.32	502.52	502.52
Colour	Black	Black	Black	Black
Crystal dimensions (mm)	0.31 × 0.14 × 0.12	0.33 × 0.19 × 0.04	0.35 × 0.32 × 0.20	0.20 × 0.17 × 0.07
Space group	*P* 1	*P* 1	*P* 1	*P* 1
*a* (Å)	7.5693 (3)	6.6911 (2)	7.7274 (4)	7.2867 (5)
*b* (Å)	13.0300 (3)	7.8201 (3)	13.1422 (6)	7.7545 (4)
*c* (Å)	18.5239 (5)	27.6491 (6)	13.3145 (6)	13.1305 (7)
α (°)	105.159 (2)	97.673 (2)	99.902 (4)	92.616 (5)
β (°)	90.971 (3)	90.168 (2)	106.251 (5)	103.414 (5)
γ (°)	98.382 (3)	108.973 (3)	97.225 (4)	113.979 (6)
*Z*	2	2	2	1
*V* (Å^3^)	1741.67 (9)	1354.24 (8)	1257.00 (11)	651.18 (7)
*D*_calc_ (g cm^−3^)	1.681	1.619	1.328	1.230
λ (Å)	1.54184 (Cu *K*α)	1.54184 (Cu *K*α)	1.54184 (Cu *K*α)	1.54184 (Cu *K*α)
μ (mm^−1^)	10.252	4.115	0.679	0.640
*Θ* range (°)	3.55–76.25	3.21–76.29	3.47–75.21	6.31–76.07
*T* (K)	100 (2)	293 (2)	100 (2)	293 (2)
Diffractometer type	Xcalibur Nova	Xcalibur Nova	Xcalibur Nova	Xcalibur Nova
Range of *h* *k* *l*	−9 < *h* < 9; −15 < *k* < 16; −23 < *l* < 21	−8 < *h* < 8; −9 < *k* < 9; −23 < *l* < 34	−8 < *h* < 9; −15 < *k* < 16; −16 < *l* < 15	−8 < *h* < 9; −9 < *k* < 7; −16 < *l* < 14
Reflections collected	15827	11901	8993	4998
Independent reflections	7163	5582	4926	2624
Observed reflections (*I* ≥ 2σ)	6810	5100	4067	1975
Absorption correction	Multi-scan	Multi-scan	Multi-scan	Multi-scan
*T*_min_, *T*_max_	0.3460, 1.0000	0.5628, 1.0000	0.3544, 1.0000	0.7450, 1.0000
*R*_int_	0.0504	0.0433	0.0708	0.0210
*R*(*F*)	0.0381	0.0434	0.0815	0.1198
*R*_w_(*F* ^2^)	0.1125	0.1177	0.2380	0.3926
Goodness of fit	0.898	1.042	1.699	1.558
H atom treatment	Constrained	Constrained	Constrained	Constrained
No. of parameters	442	370	352	190
No. of restraints	0	0	0	90
Δρ_max_, Δρ_min_, Δρ_r.m.s._ (eÅ^–3^)	1.313, −1.421, 0.132	0.873, −0.679, 0.107	0.434, −0.351, 0.085	0.853, −0.783, 0.098

**Table:** 

Compound	**14**·(TCNQ)_2_ 100 K	**14**·(TCNQ)_2_ RT
Empirical formula	C_36_H_16_N_10_	C_36_H_16_N_10_
Formula weight (g mol^−1^)	588.59	588.59
Colour	Black	Black
Crystal dimensions (mm)	0.21 × 0.09 × 0.08	0.32 × 0.11 × 0.09
Space group	*P* 1	*P* 1
*a* (Å)	7.1249 (13)	7.2179 (10)
*b* (Å)	7.6572 (13)	7.6887 (14)
*c* (Å)	14.4249 (10)	14.5445 (13)
α (°)	89.017 (10)	89.176 (10)
β (°)	83.589 (12)	82.570 (9)
γ (°)	68.340 (17)	69.284 (14)
*Z*	1	1
*V* (Å^3^)	726.6 (2)	748.2 (2)
*D*_calc_ (g cm^−3^)	1.345	1.306
λ (Å)	1.54184 (Cu *K*α)	1.54184 (Cu *K*α)
μ (mm^−1^)	0.685	0.665
*Θ* range (°)	6.17–75.55	6.15–74.72
*T* (K)	100 (2)	293 (2)
Diffractometer type	Xcalibur Nova	Xcalibur Nova
Range of *h* *k* *l*	−8 < *h* < 6; −9 < *k* < 9; −18 < *l* < 18	−9 < *h* < 8; −9 < *k* < 9; −17 < *l* < 18
Reflections collected	5931	6183
Independent reflections	2996	3038
Observed reflections (*I* ≥ 2σ)	2391	2470
Absorption correction	Multi-scan	Multi-scan
*T*_min_, *T*_max_	0.6330, 1.0000	0.6757, 1.0000
*R*_int_	0.0572	0.0374
*R*(*F*)	0.1281	0.1078
*R*_w_(*F* ^2^)	0.3816	0.3477
Goodness of fit	1.481	1.400
H atom treatment	Constrained	Constrained
No. of parameters	217	217
No. of restraints	0	0
Δρ_max_, Δρ_min_, Δρ_r.m.s._ (eÅ^–3^)	1.251, −0.400, 0.120	0.918, −0.236, 0.080
