# Supplementary material for: Semiconductive 2D arrays of pancake-bonded oligomers of partially charged TCNQ radicals
Source: IUCrJ. 2022 May 28;9(Pt 4):449–67. doi: 10.1107/S2052252522004717 (PMC9252159; doi:10.1107/S2052252522004717)
Supplement: Supplementary file 2 [file m-09-00449-sup2.pdf]

IUCrJ

Volume 9 (2022)

Supporting information for article:

Semiconductive 2D arrays of pancake-bonded oligomers of partially charged TCNQ radicals

Krešimir Molčanov, Valentina Milašinović, Biserka Kojić-Prodić, Nadica Maltar-Strmečki, Jiangyang You, Ana Šantić, Lidija Kanižaj, Vladimir Stilinović and Luka Fotović

Contents

S1 ORTEP diagrams of cations and TCNQ anions

S2 Disorder of cations

S3 Details on molecular geometries of TCNQ anions

S4 Diagrams of crystal packings

S5 Powder EPR results

S6 Single-crystal EPR results

S7 IR spectra

S8 Powder diffractograms

S1. ORTEP diagrams of cations and TCNQ anions

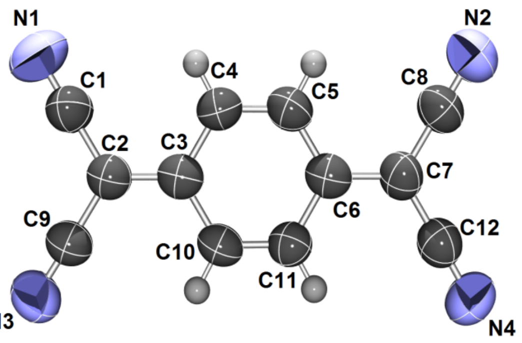

1_2·Br·$(TCNQ)_2$

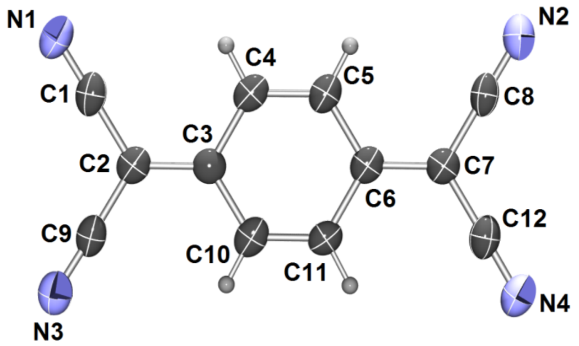

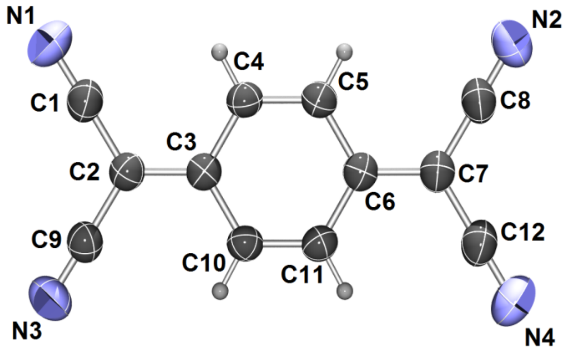

2·$(TCNQ)_2$ (100 K) **(RT)**

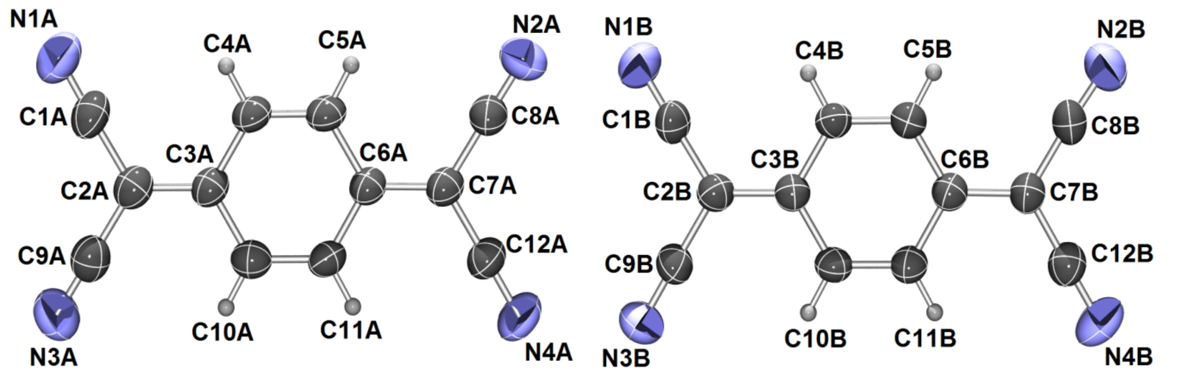

3·(TCNQ)$_2$

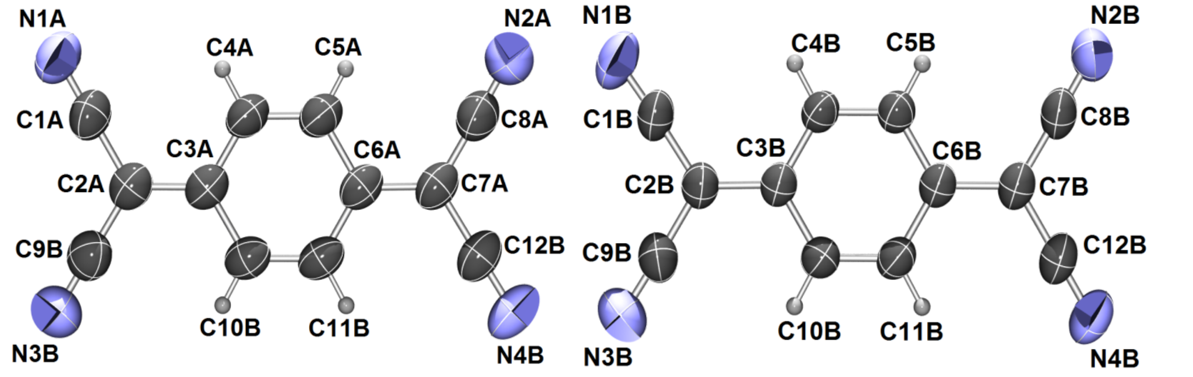

4·(TCNQ)$_2$

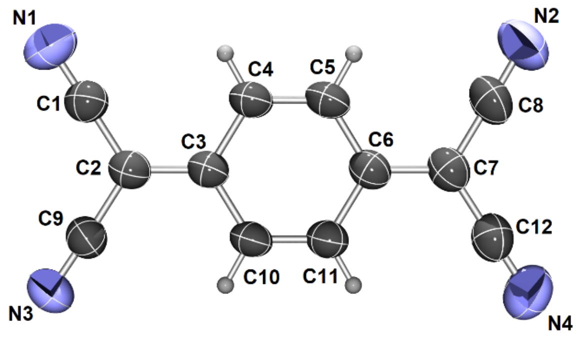

5·(TCNQ)$_2$

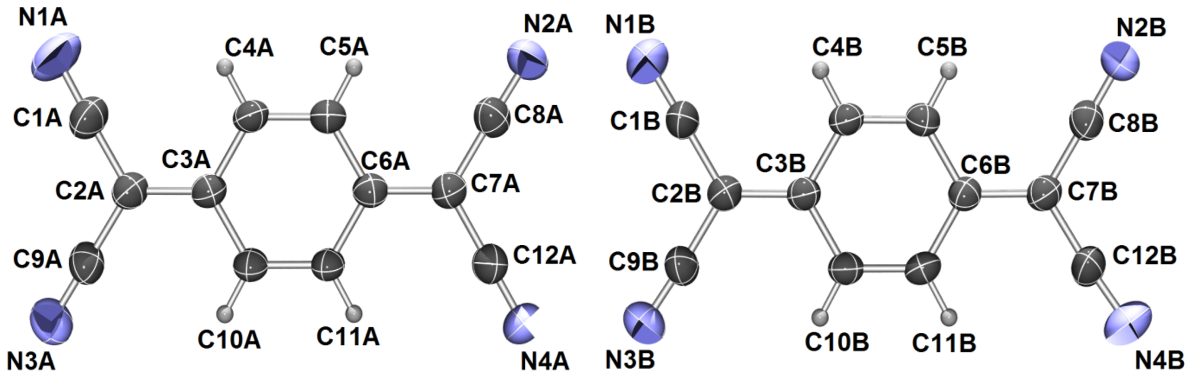

6·(TCNQ)$_2$

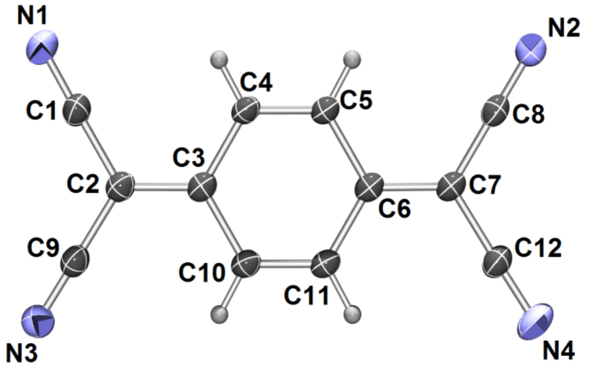

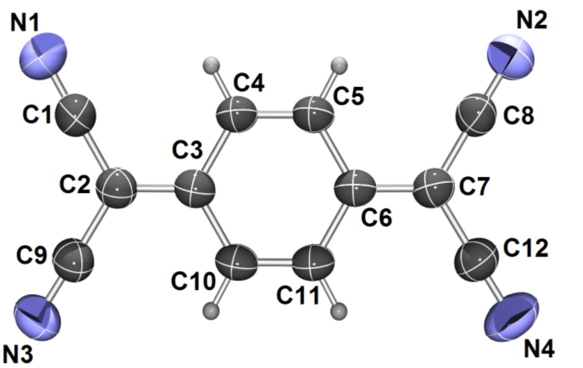

7·(TCNQ)$_2$ (100 K) **(RT)**

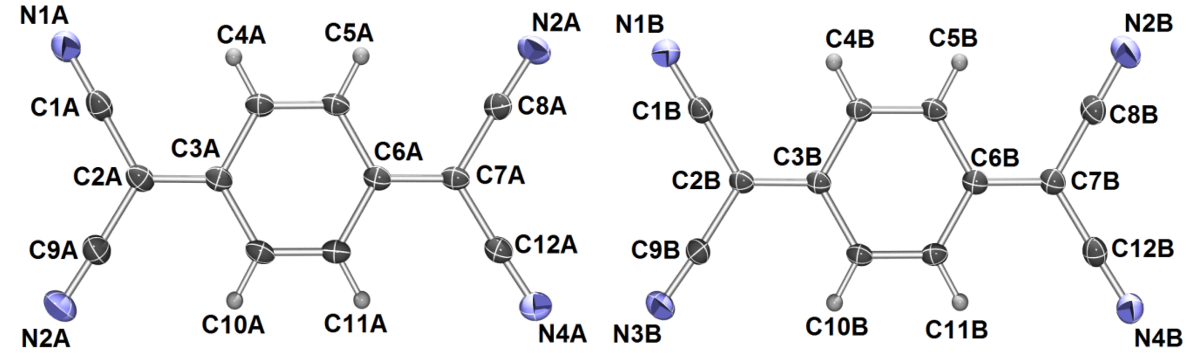

8·(TCNQ)$_2$

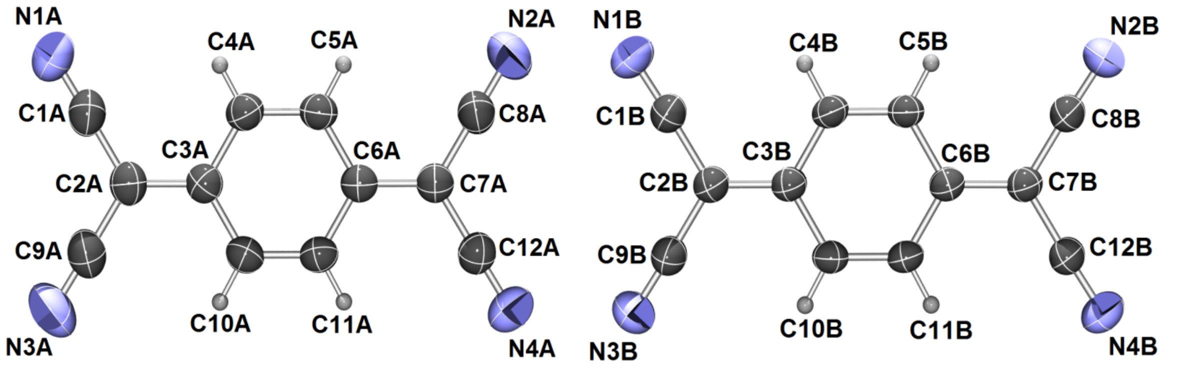

9·(TCNQ)$_4$

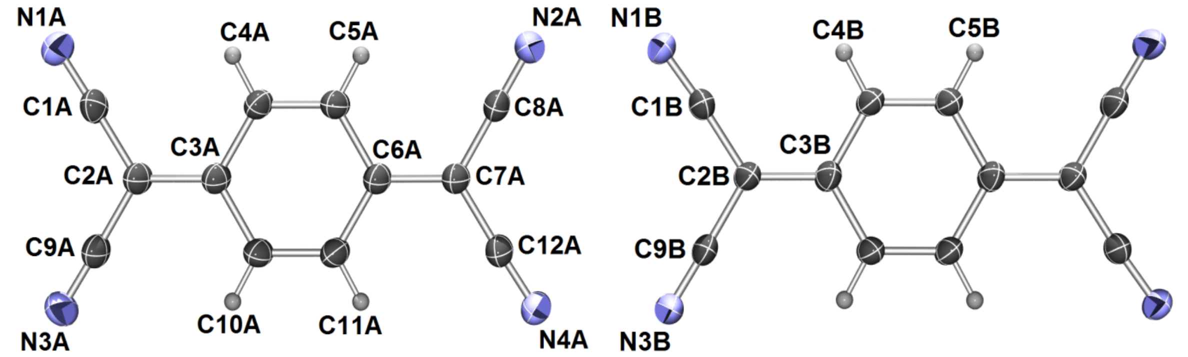

10·(TCNQ)$_3$

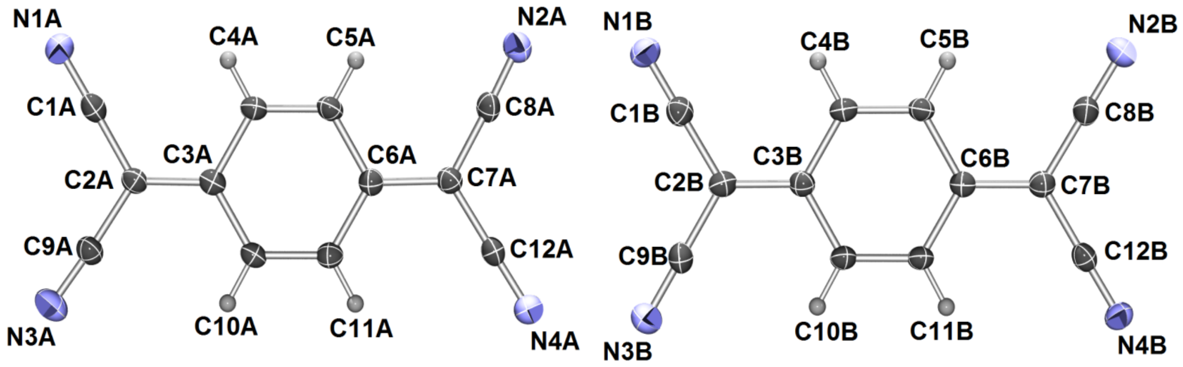

11_2·I·(TCNQ)$_2$

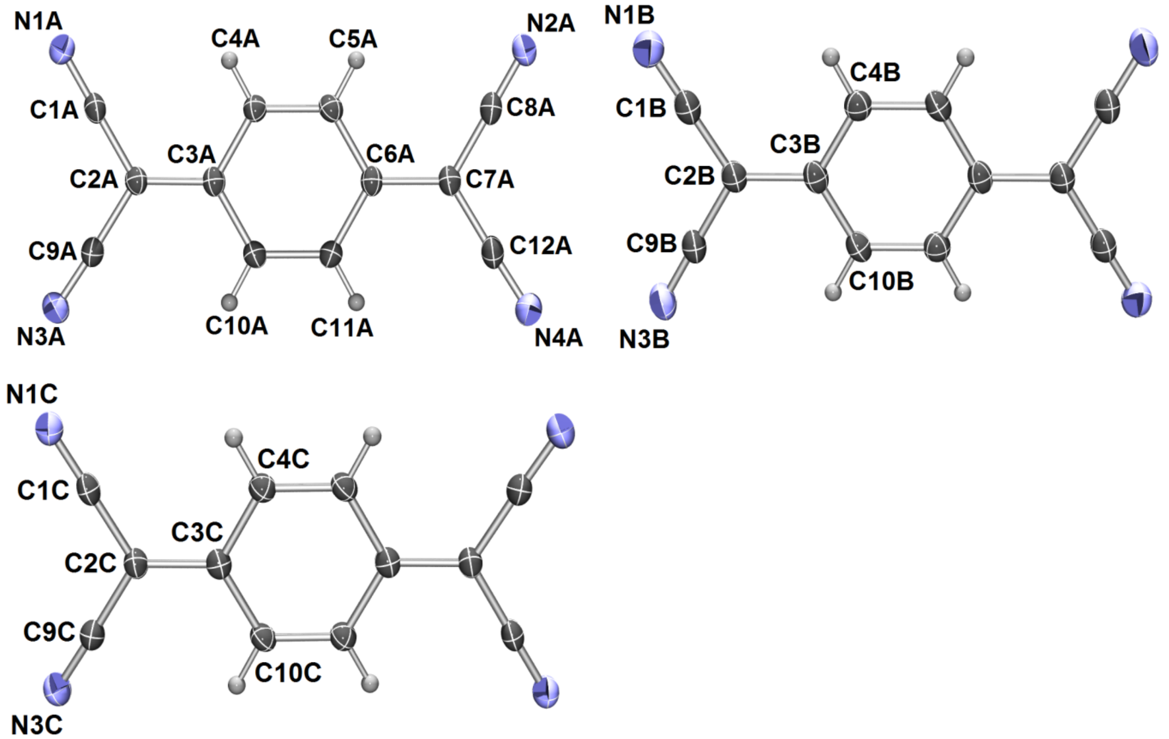

12·(TCNQ)$_2$

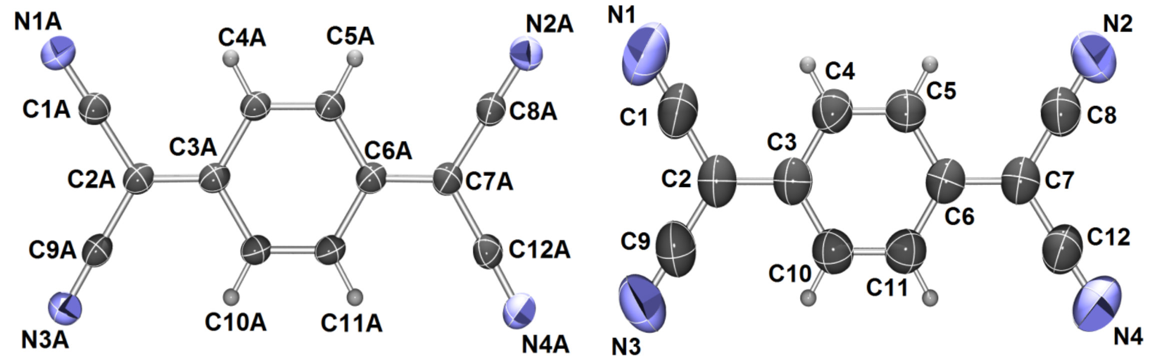

13·(TCNQ)$_2$

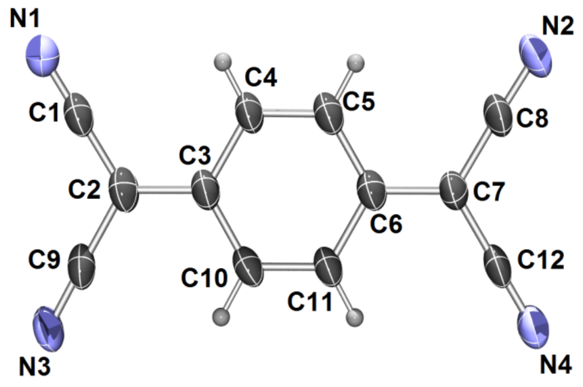

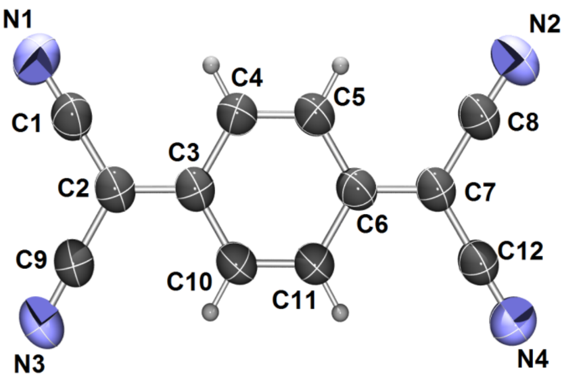

14·(TCNQ)$_2$ (100 K) **(RT)**

Figure S1 ORTEP-3 drawings of TCNQ$^{\delta-}$ radical anions in the studied compounds with atom numbering scheme (only symmetry-independent part is labelled). Displacement ellipsoids are drawn for the probability of 50 % and hydrogen atoms are shown as spheres of arbitrary radii.

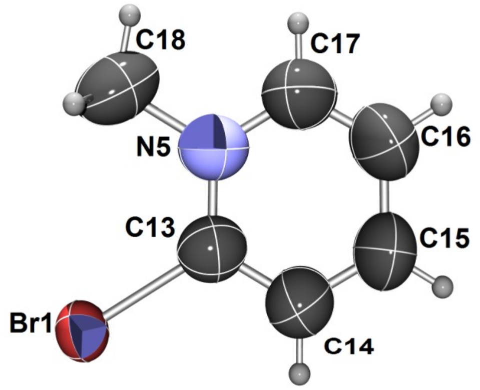

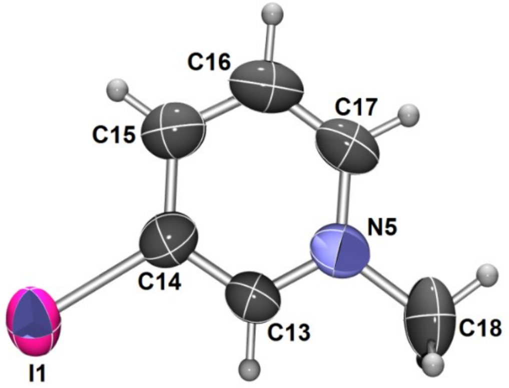

1$_2$·Br·(TCNQ)$_2$ **3·(TCNQ)$_2$**

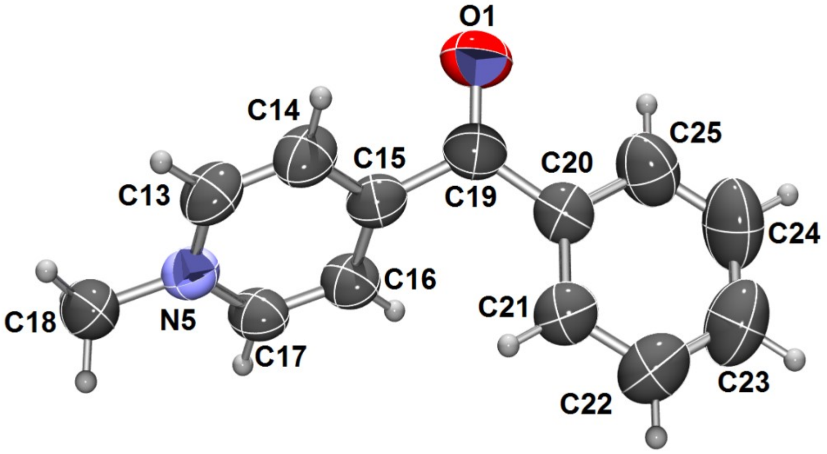

4·(TCNQ)$_2$

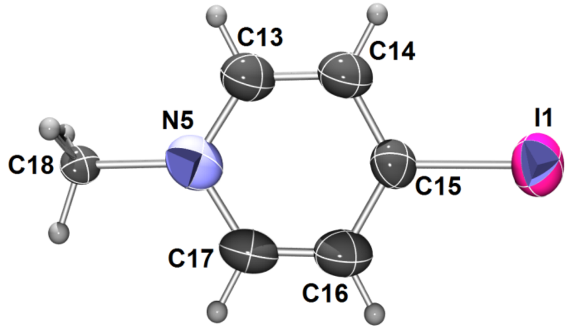

6·(TCNQ)$_2$

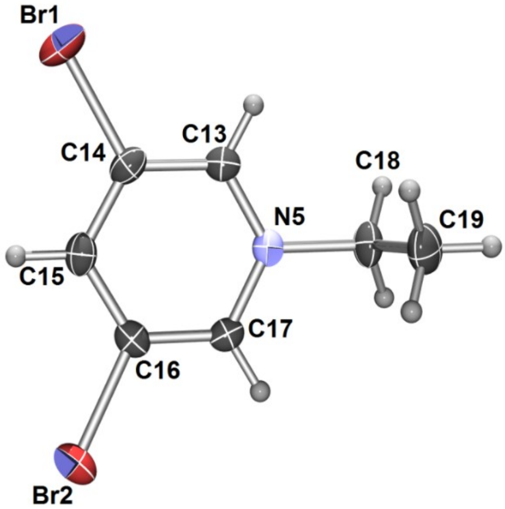

8·(TCNQ)$_2$

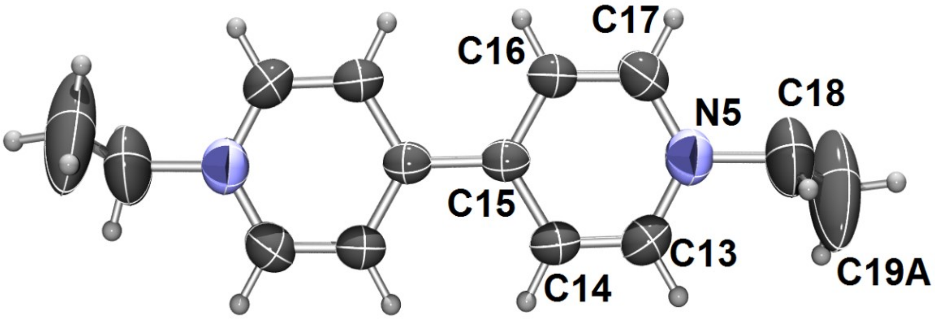

9·(TCNQ)$_4$

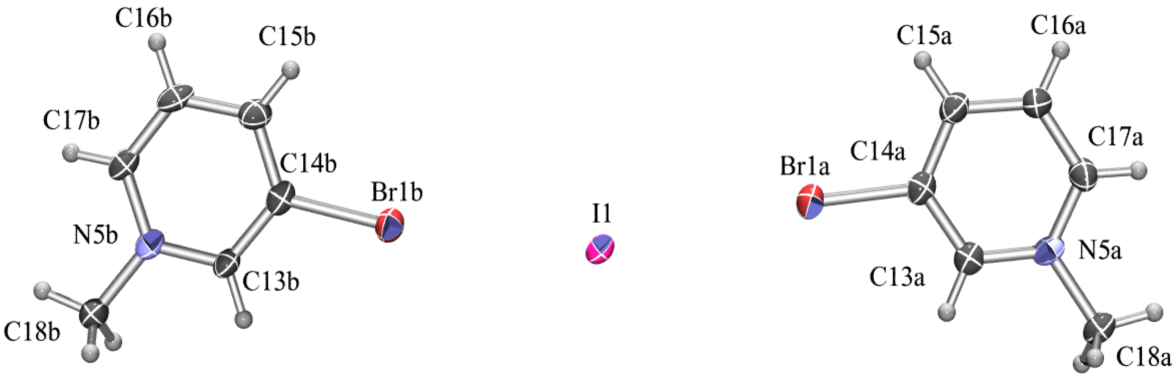

11$_2$·I·(TCNQ)$_2$

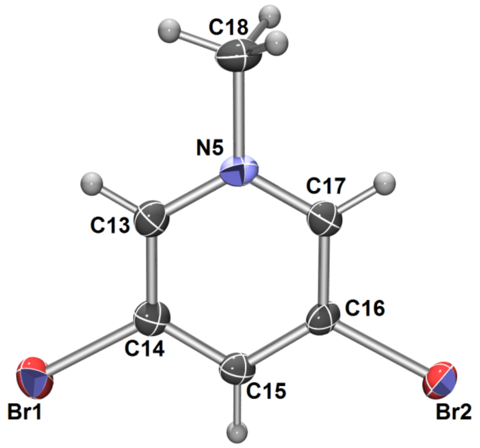

13·(TCNQ)$_2$ (100 K)

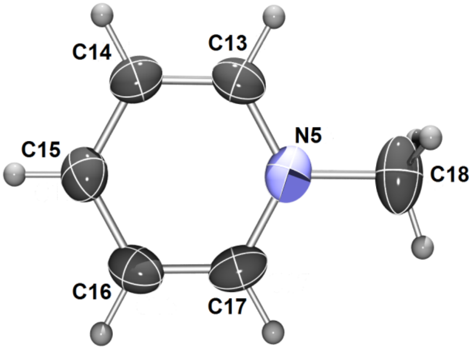

12·(TCNQ)$_2$

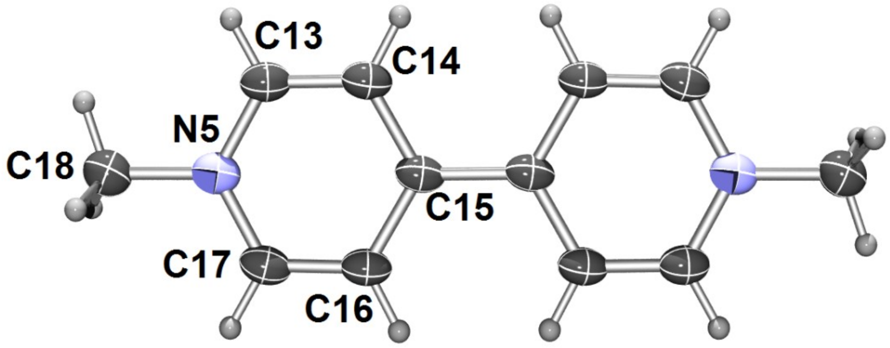

10·(TCNQ)$_3$

Figure S2 ORTEP-3 drawings of non-disordered cations in the studied compounds with atom numbering scheme (only symmetry-independent part is labelled). Displacement ellipsoids are drawn for the probability of 50 % and hydrogen atoms are shown as spheres of arbitrary radii.

S2. Disorder of the cations

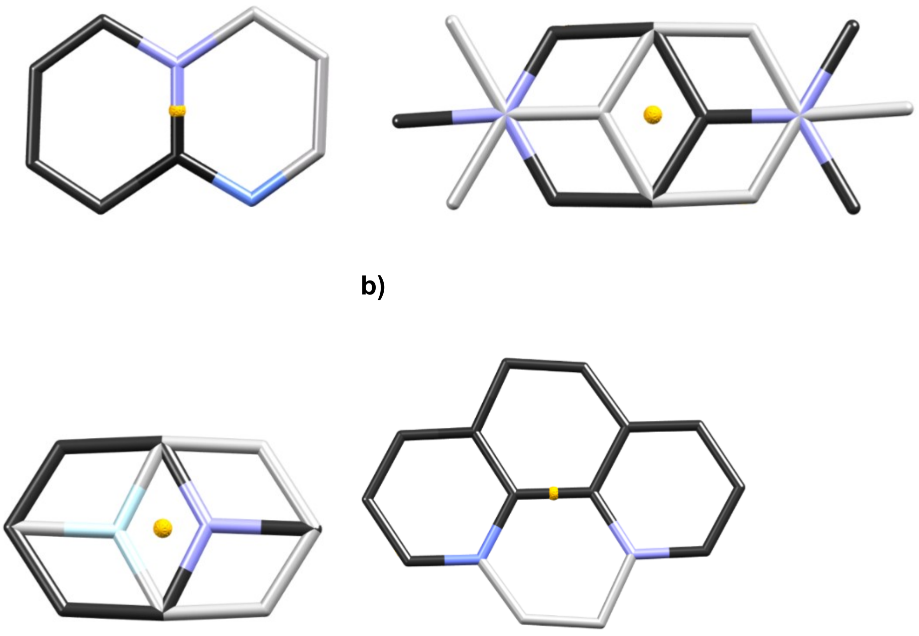

a) **b)**

c) **d)**

Figure S3 Schematic representation of disorder of cations in a) **2·(TCNQ)$_2$**, b) **5·(TCNQ)$_2$**, c) **13·(TCNQ)$_2$** at RT and d) **14·(TCNQ)$_2$**. Inversion centre is shown as a yellow sphere. For clarity, one orientation is showed in light grey colour and hydrogen atoms have been omitted.

S3. Details on molecular geometries of TCNQ anions

Table S1 Bond lengths in TCNQ radicals (Å). Only symmetry-independent bonds are listed.

	**1_2·Br·(TCNQ)$_2$**	**2·(TCNQ)$_2$, 100K**	**2·(TCNQ)$_2$, RT**	**3·(TCNQ)$_2$ A**	**3·(TCNQ)$_2$ B**	**4·(TCNQ)$_2$ A**	**4·(TCNQ)$_2$ B**	**5·(TCNQ)$_2$**	**6·(TCNQ)$_2$ A**
C1-C2	1.44(1)	1.433(8)	1.420(3)	1.45(1)	1.430(10)	1.42(2)	1.416(2)	1.421(3)	1.41(1)
C2-C3	1.37(1)	1.372(7)	1.392(3)	1.382(9)	1.390(8)	1.389(2)	1.401(2)	1.386(3)	1.393(9)
C2-C9	1.423(9)	1.432(8)	1.424(3)	1.43(1)	1.423(9)	1.422(2)	1.417(2)	1.425(3)	1.42(1)
C3-C4	1.433(9)	1.458(7)	1.433(3)	1.44(1)	1.431(8)	1.430(2)	1.426(2)	1.441(2)	1.439(9)
C3-C10	1.45(1)	1.428(7)	1.432(3)	1.429(9)	1.430(8)	1.438(2)	1.429(1)	1.440(3)	1.430(9)
C4-C5	1.35(1)	1.344(7)	1.351(3)	1.34(1)	1.352(9)	1.347(2)	1.356(1)	1.352(3)	1.359(9)
C5-C6	1.41(1)	1.432(6)	1.436(3)	1.441(9)	1.427(9)	1.441(2)	1.428(1)	1.430(3)	1.431(9)
C6-C7	1.39(1)	1.380(7)	1.392(3)	1.391(8)	1.407(8)	1.388(2)	1.406(2)	1.391(3)	1.393(9)
C6-C11	1.436(9)	1.460(7)	1.428(3)	1.433(9)	1.433(8)	1.430(2)	1.424(2)	1.437(3)	1.436(8)
C7-C8	1.42(1)	1.433(7)	1.420(3)	1.43(1)	1.428(10)	1.420(2)	1.417(2)	1.431(4)	1.43(1)
C7-C12	1.42(1)	1.424(7)	1.429(3)	1.418(9)	1.407(9)	1.429(2)	1.426(2)	1.425(3)	1.42(1)
C10-C11	1.34(1)	1.340(7)	1.353(3)	1.369(9)	1.366(8)	1.347(2)	1.352(2)	1.345(3)	1.361(9)
C1-N1	1.13(1)	1.144(7)	1.148(3)	1.12(1)	1.141(12)	1.142(2)	1.141(2)	1.154(3)	1.16(1)
C8-N2	1.13(1)	1.165(8)	1.146(3)	1.14(1)	1.140(11)	1.148(3)	1.144(2)	1.138(4)	1.14(1)
C9-N3	1.15(1)	1.174(8)	1.145(3)	1.12(1)	1.162(14)	1.145(3)	1.143(2)	1.142(3)	1.15(1)
C12-N4	1.17(1)	1.152(7)	1.144(4)	1.15(1)	1.156(14)	1.137(2)	1.146()	1.147(4)	1.16(1)

	**6·(TCNQ)$_2$ B**	**7·(TCNQ)$_2$, 100K**	**7·(TCNQ)$_2$, RT**	**8·(TCNQ)$_2$ A**	**8·(TCNQ)$_2$ B**	**9·(TCNQ)$_4$ A**	**9·(TCNQ)$_4$ B**	**10·(TCNQ)$_3$ A**	**10·(TCNQ)$_3$ B**
C1-C2	1.42(1)	1.430(2)	1.425(2)	1.419(4)	1.433(4)	1.427(2)	1.424(2)	1.429(2)	1.423(2)
C2-C3	1.392(9)	1.389(2)	1.394(2)	1.403(3)	1.391(3)	1.391(2)	1.400(2)	1.394(2)	1.412(2)
C2-C9	1.42(1)	1.424(2)	1.425(2)	1.430(3)	1.431(3)	1.423(2)	1.421(2)	1.429(2)	1.416(2)
C3-C4	1.441(9)	1.437(2)	1.437(2)	1.438(3)	1.438(3)	1.434(2)	1.428(2)	1.437(2)	1.424(2)
C3-C10	1.439(9)	1.442(2)	1.431(2)	1.430(4)	1.443(4)	1.433(2)	1.434(2)	1.442(2)	1.431(2)
C4-C5	1.346(8)	1.354(2)	1.354(2)	1.354(3)	1.358(3)	1.349(2)	1.358(2)	1.355(2)	1.364(2)

C5-C6	1.434(8)	1.444(2)	1.433(2)	1.439(4)	1.431(4)	1.439(2)	1.435(2)	1.439(2)	1.431(2)
C6-C7	1.396(9)	1.389(2)	1.389(2)	1.391(3)	1.401(3)	1.384(2)	1.400(2)	1.397(2)	
C6-C11	1.436(9)	1.434(2)	1.441(2)	1.437(3)	1.438(3)	1.432(2)	1.428(2)	1.438(2)	
C7-C8	1.42(1)	1.427(2)	1.427(2)	1.429(3)	1.427(3)	1.422(2)	1.421(2)	1.425(2)	
C7-C12	1.43(1)	1.433(2)	1.424(2)	1.429(4)	1.423(4)	1.427(2)	1.424(2)	1.429(2)	
C10-C11	1.356(9)	1.354(2)	1.355(2)	1.355(3)	1.356(3)	1.354(2)	1.355(2)	1.355(2)	
C1-N1	1.14(1)	1.153(2)	1.138(3)	1.156(4)	1.149(4)	1.145(2)	1.144(2)	1.152(2)	1.151(2)
C8-N2	1.1651)	1.149(2)	1.140(2)	1.149(3)	1.153(3)	1.140(2)	1.147(2)	1.154(2)	
C9-N3	1.15(1)	1.154(2)	1.142(2)	1.147(3)	1.149(3)	1.144(2)	1.145(2)	1.150(2)	1.144(3)
C12-N4	1.16(1)	1.150(2)	1.145(2)	1.151(4)	1.154(4)	1.144(2)	1.143(2)	1.153(2)	

	**11$_2$·I·(TCNQ)$_2$, 100K, A**	**11$_2$·I·(TCNQ)$_2$, 100K, B**	**12·(TCNQ)$_2$ A**	**12·(TCNQ)$_2$ B**	**12·(TCNQ)$_2$ C**	**13·(TCNQ)$_2$, 100K, A**	**13·(TCNQ)$_2$, 100K, B**
C1-C2	1.427(5)	1.423(5)	1.428(4)	1.421(4)	1.432(4)	1.431(3)	1.433(3)
C2-C3	1.395(4)	1.401(4)	1.396(3)	1.414(3)	1.382(3)	1.377(3)	1.382(3)
C2-C9	1.427(5)	1.423(5)	1.431(3)	1.426(4)	1.433(3)	1.437(3)	1.437(3)
C3-C4	1.439(4)	1.430(4)	1.433(3)	1.425(4)	1.443(3)	1.438(3)	1.445(3)
C3-C10	1.428(4)	1.431(4)	1.433(3)	1.420(4)	1.436(4)	1.445(3)	1.435(3)
C4-C5	1.357(4)	1.369(4)	1.360(3)	1.370(4)	1.352(3)	1.349(3)	1.344(3)
C5-C6	1.436(4)	1.435(4)	1.427(4)	1.370(4)	1.352(3)	1.441(3)	1.440(3)
C6-C7	1.405(4)	1.396(4)	1.402(3)			1.383(3)	1.380(3)
C6-C11	1.431(4)	1.436(4)	1.438(3)			1.441(3)	1.442(3)
C7-C8	1.422(5)	1.431(5)	1.425(3)			1.432(3)	1.428(3)
C7-C12	1.421(4)	1.425(5)	1.424(4)			1.436(3)	1.431(3)
C10-C11	1.364(4)	1.366(4)	1.365(3)			1.350(3)	1.351(3)
C1-N1	1.151(5)	1.151(5)	1.152(4)	1.155(4)	1.149(4)	1.151(3)	1.147(3)
C8-N2	1.151(5)	1.150(5)	1.155(3)			1.152(3)	1.158(3)
C9-N3	1.153(5)	1.157(5)	1.149(3)	1.145(4)	1.147(3)	1.151(3)	1.147(3)
C12-N4	1.154(4)	1.154(5)	1.152(4)			1.151(3)	1.146(3)

	**13·(TCNQ)$_2$, RT**	**14·(TCNQ)$_2$, 100K**	**14·(TCNQ)$_2$, RT**
C1-C2	1.422(5)	1.431(5)	1.425(4)
C2-C3	1.399(4)	1.394(4)	1.402(4)
C2-C9	1.413(5)	1.433(4)	1.418(4)
C3-C4	1.424(4)	1.443(4)	1.440(4)
C3-C10	1.433(4)	1.428(5)	1.427(4)
C4-C5	1.358(4)	1.349(4)	1.353(4)
C5-C6	1.432(4)	1.433(5)	1.436(4)
C6-C7	1.390(4)	1.399(4)	1.391(4)
C6-C11	1.435(4)	1.434(4)	1.438(4)
C7-C8	1.418(4)	1.430(4)	1.426(4)
C7-C12	1.419(4)	1.426(5)	1.424(4)
C10-C11	1.351(4)	1.362(4)	1.363(4)
C1-N1	1.146(6)	1.131(5)	1.134(4)
C8-N2	1.133(5)	1.154(4)	1.129(4)
C9-N3	1.139(5)	1.158(5)	1.148(4)
C12-N4	1.134(5)	1.149(5)	1.144(4)

Table S2 Estimated charge by geometric correlations. Averaged bond lengths are given.

ring	*a*	*b*	*c*	*d*	*b*−*a*	*c*−*d*	*c*/(*b*+*d*)	(*a*+*c*)/(*b*+*d*)	Estimated charge[a]
**1_2·Br·(TCNQ)$_2$**	1.342	1.433	1.382	1.426	0.091	-0.044	0.483	0.988	0.31
**2·(TCNQ)$_2$, 100K**	1.342	1.445	1.376	1.431	0.103	-0.055	0.478	0.993	0.10
**2·(TCNQ)$_2$, RT**	1.352	1.432	1.392	1.423	0.080	-0.031	0.488	0.989	0.48
**3·(TCNQ)$_2$, A**	1.353	1.436	1.386	1.436	0.083	-0.050	0.483	0.988	0.27
**3·(TCNQ)$_2$, B**	1.360	1.430	1.398	1.422	0.070	-0.024	0.490	0.989	0.59
**4·(TCNQ)$_2$, A**	1.347	1.435	1.389	1.426	0.088	-0.037	0.485	0.988	0.39
**4·(TCNQ)$_2$, B**	1.354	1.427	1.401	1.419	0.073	-0.018	0.492	0.986	0.68
**5·(TCNQ)$_2$**	1.349	1.437	1.388	1.426	0.088	-0.038	0.485	0.990	0.37
**6·(TCNQ)$_2$, A**	1.360	1.433	1.393	1.422	0.073	-0.029	0.488	0.992	0.50
**6·(TCNQ)$_2$, B**	1.351	1.438	1.394	1.426	0.087	-0.032	0.487	0.989	0.45
**7·(TCNQ)$_2$, 100K**	1.354	1.439	1.389	1.429	0.085	-0.040	0.484	0.991	0.35
**7·(TCNQ)$_2$, RT**	1.354	1.436	1.392	1.425	0.082	-0.033	0.487	0.990	0.44
**8·(TCNQ)$_2$, A**	1.354	1.436	1.397	1.427	0.082	-0.030	0.488	0.987	0.50
**8·(TCNQ)$_2$, B**	1.357	1.437	1.396	1.428	0.080	-0.032	0.487	0.989	0.47
**9·(TCNQ)$_4$, A**	1.352	1.435	1.389	1.425	0.083	-0.036	0.486	0.990	0.41
**9·(TCNQ)$_4$, B**	1.356	1.431	1.400	1.423	0.075	-0.023	0.491	0.987	0.60
**11_2·I·(TCNQ)$_2$, A**	1.361	1.433	1.400	1.424	0.072	-0.024	0.490	0.989	0.58
**11_2·I·(TCNQ)$_2$, B**	1.367	1.433	1.398	1.425	0.066	-0.027	0.489	0.991	0.55
**12·(TCNQ)$_2$, A**	1.363	1.433	1.399	1.427	0.070	-0.028	0.489	0.989	0.55
**12·(TCNQ)$_2$, B**	1.370	1.423	1.414	1.424	0.053	-0.010	0.497	0.984	0.86
**12·(TCNQ)$_2$, C**	1.352	1.440	1.382	1.432	0.088	-0.050	0.481	0.992	0.22
**13·(TCNQ)$_2$, 100K, A**	1.350	1.441	1.380	1.434	0.091	-0.054	0.480	0.991	0.17
**13·(TCNQ)$_2$, 100K, B**	1.347	1.440	1.381	1.432	0.093	-0.051	0.481	0.991	0.20
**13·(TCNQ)$_2$, RT**	1.354	1.431	1.395	1.422	0.077	-0.027	0.489	0.989	0.54
**14·(TCNQ)$_2$, 100K**	1.356	1.434	1.396	1.430	0.078	-0.034	0.487	0.987	0.48

**14·(TCNQ)$_2$, RT**	1.358	1.435	1.396	1.423	0.077	-0.027	0.488	0.991	0.52
**10·(TCNQ)$_3$, A**[b]	1.355	1.439	1.396	1.428	0.084	-0.032	0.487	0.989	0.45
**10·(TCNQ)3, B**[b]	1.364	1.429	1.412	1.420	0.065	-0.008	0.496	0.986	0.82
average	1.354(7)	1.435(5)	1.392(8)	1.426(4)	0.08(1)	-0.03(1)	0.487(4)	0.989(2)	0.4(2)
TCNQ neutral[c]	1.343	1.439	1.379	1.431	0.096	-0.052	0.480	0.990	
$TCNQ^{-}$[c]	1.355	1.423	1.407	1.419	0.068	-0.012	0.495	0.983	

[a] from $c/(b+d)$; [b] data for trimer were not used to calculate average; [c] data from Herbstein & Kapon (2008).

S4. Diagrams of crystal packing

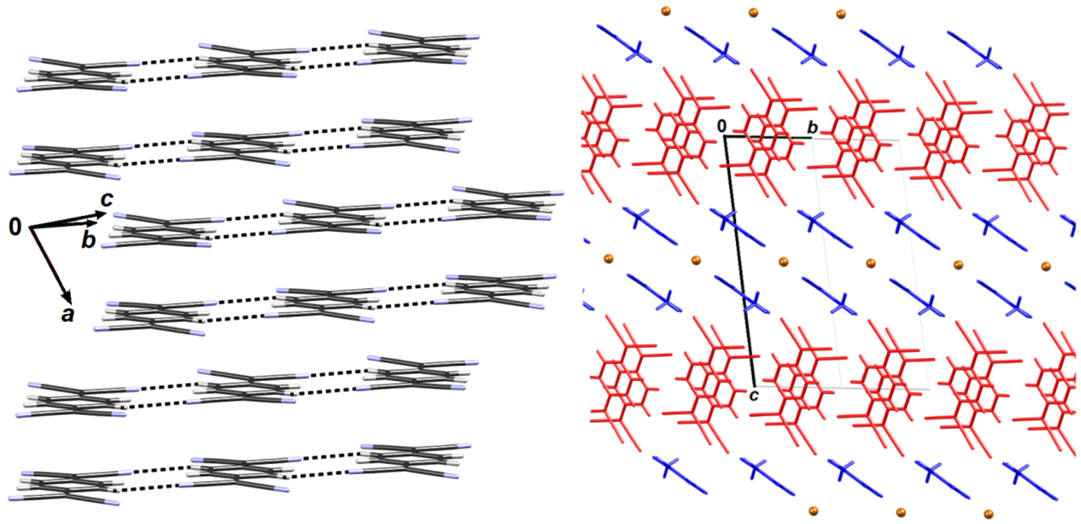

a) b)

Figure S4 Crystal packing of **1_2·Br·(TCNQ)$_2$**: a) 2D 'brick-wall' array of pancake-bonded dimers, b) crystal packing viewed in the direction [100] showing alternating layers of TCNQ$^{\delta-}$ anions (red), cations (blue) and bromide anions (brown; shown as spheres of arbitrary radii).

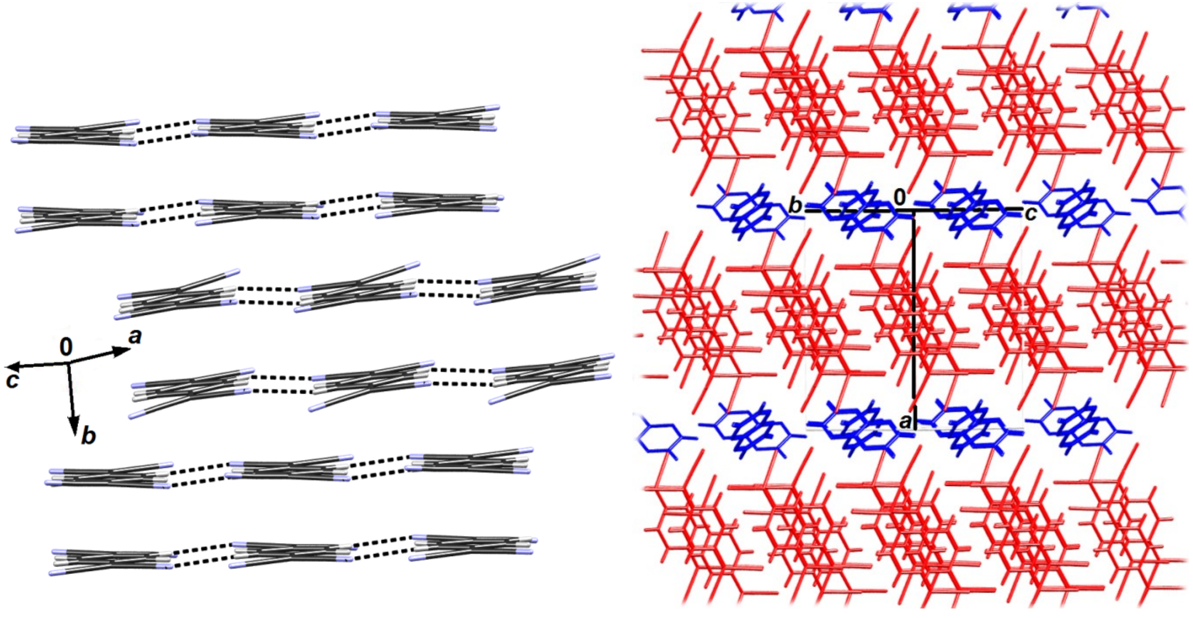

a) b)

Figure S5 Crystal packing of **2·(TCNQ)$_2$**: a) 2D 'brick-wall' array of pancake-bonded dimers, b) crystal packing viewed in the direction [011] showing alternating layers of TCNQ$^{\delta-}$ anions (red) and cations (blue).

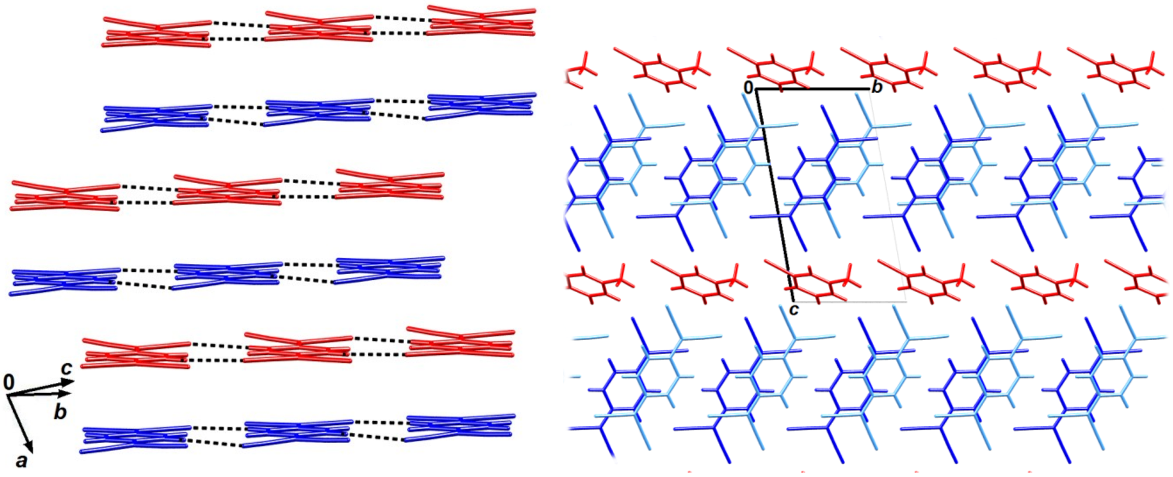

a) b)

Figure S6 Crystal packing of **3·(TCNQ)₂**: a) 2D 'brick-wall' array of pancake-bonded dimers (cation A is blue and cation B is red), b) crystal packing viewed in the direction [100] showing alternating layers of TCNQ$^{\delta-}$ anions (blue) and cations (red).

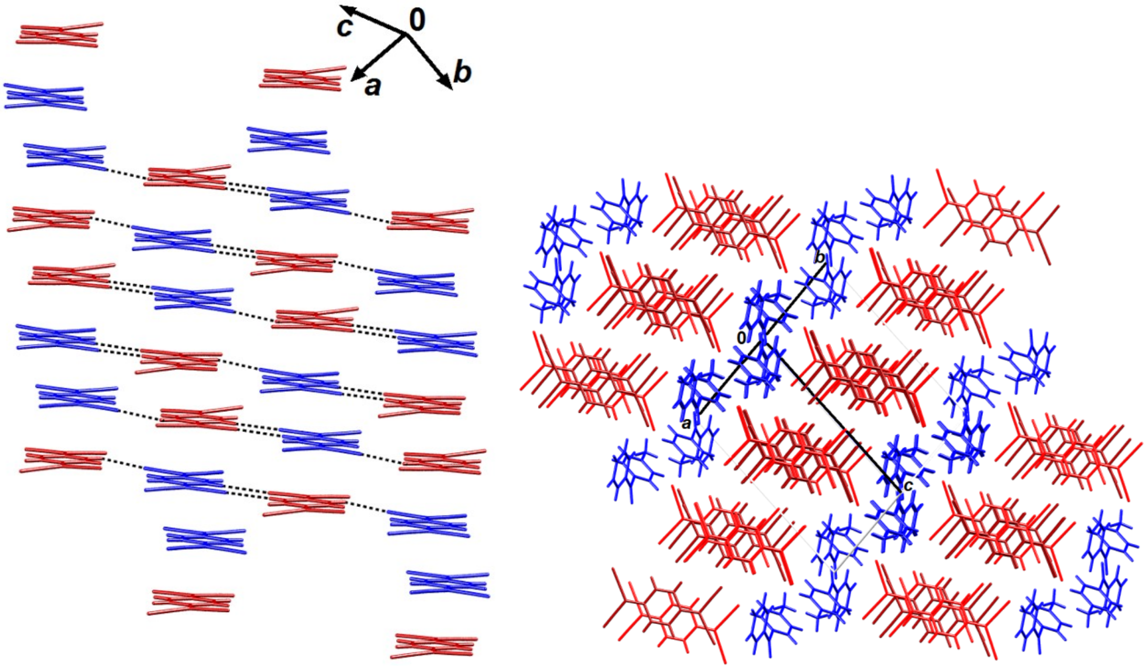

a) b)

Figure S7 Crystal packing of **4·(TCNQ)₂**: a) 2D 'brick-wall' array of pancake-bonded tetramers (cation A is red and cation B is blue), b) crystal packing viewed in the direction [110] showing alternating layers of TCNQ$^{\delta-}$ anions (red) and cations (blue).

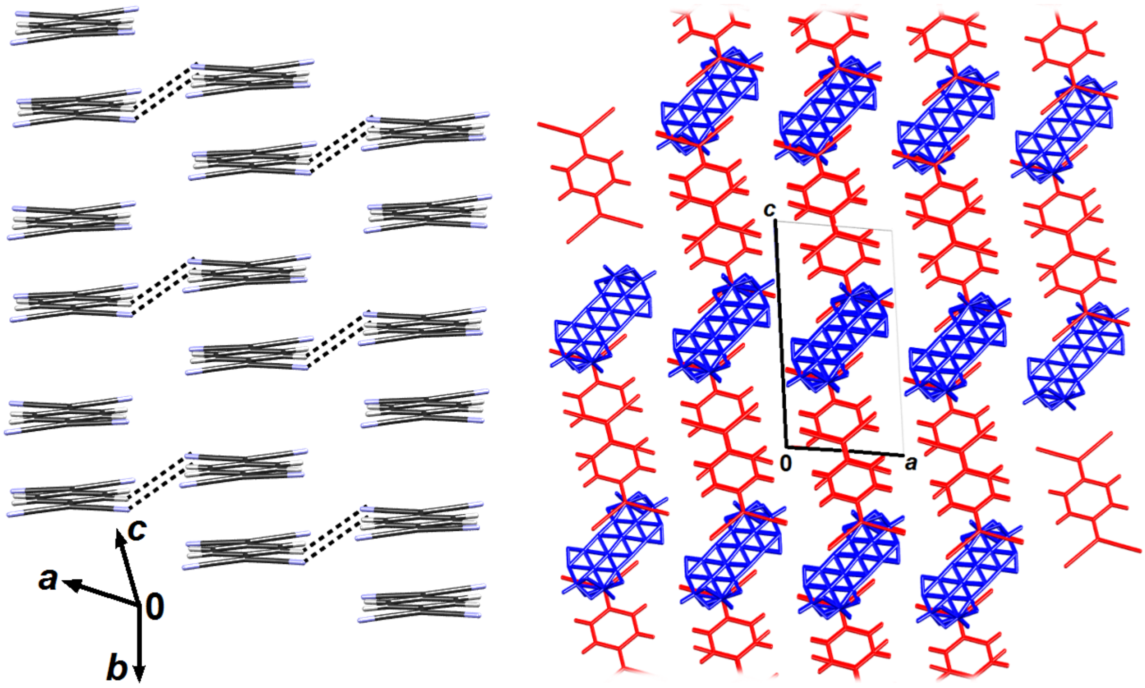

a) b)

Figure S8 Crystal packing of **5·(TCNQ)₂**: a) 2D array of pancake-bonded dimers, b) crystal packing viewed in the direction [010] showing alternating layers of TCNQ$^{\delta-}$ anions (red) and cations (blue).

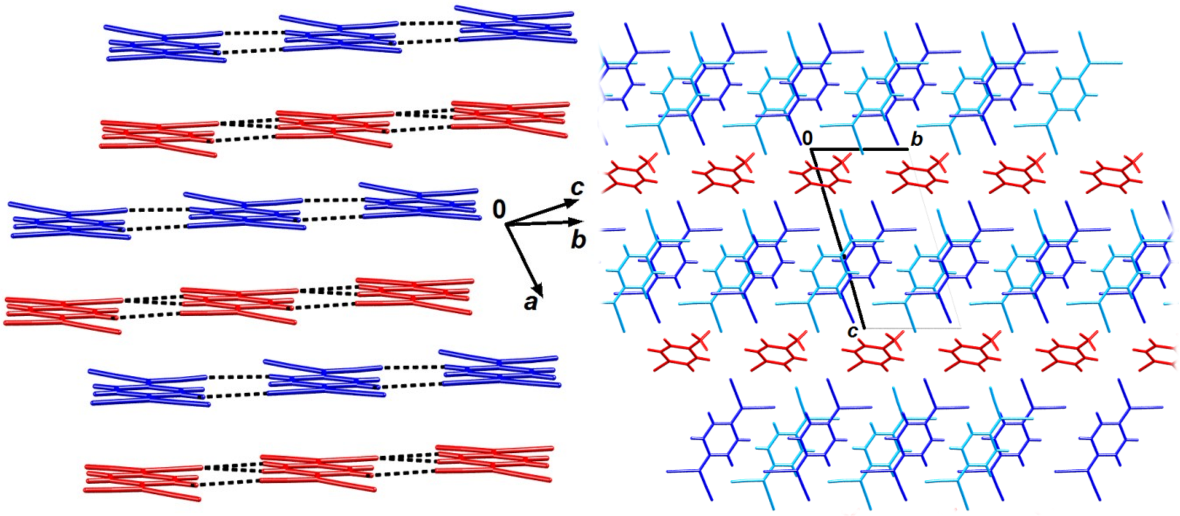

a) b)

Figure S9 Crystal packing of **6·(TCNQ)₂**: a) 2D 'brick-wall' array of pancake-bonded dimers (cation A is blue and cation B is red), b) crystal packing viewed in the direction [100] showing alternating layers of TCNQ$^{\delta-}$ anions (blue) and cations (red).

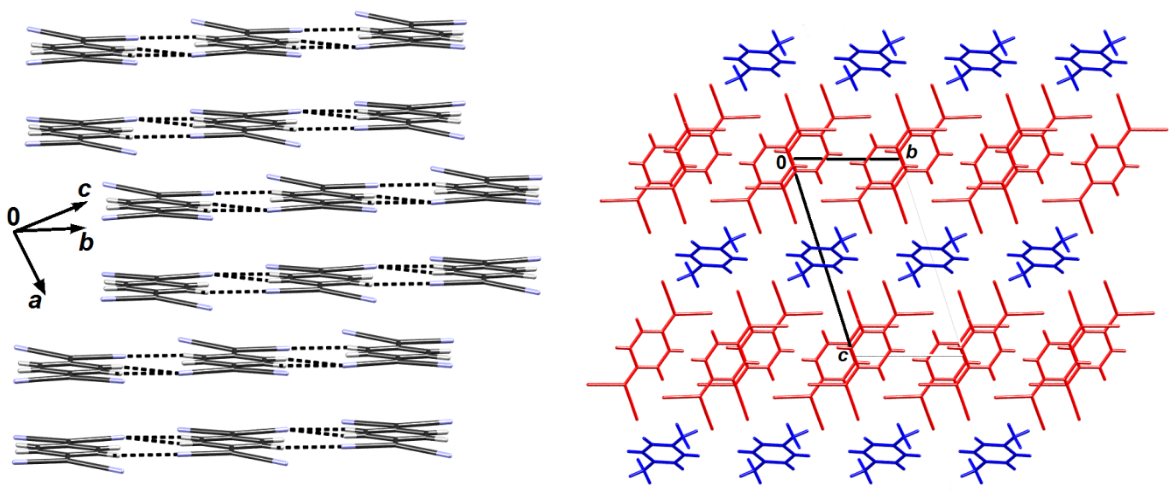

a) **b)**

Figure S10 Crystal packing of **7·(TCNQ)₂**: a) 2D 'brick-wall' array of pancake-bonded dimers, b) crystal packing viewed in the direction [100] showing alternating layers of TCNQ$^{\delta-}$ anions (red) and cations (blue).

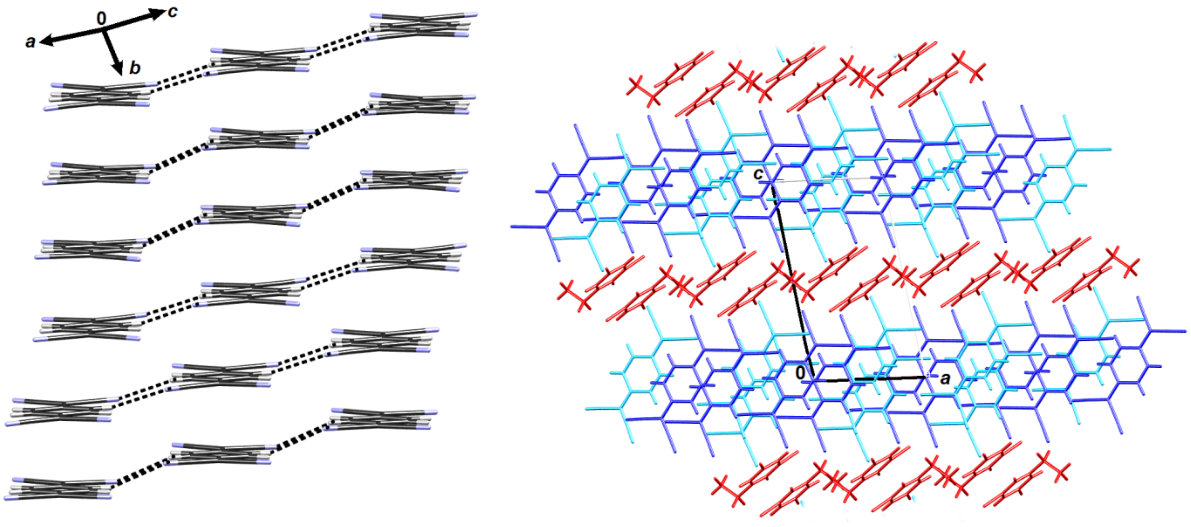

a) **b)**

Figure S11 Crystal packing of **8·(TCNQ)₂**: a) 2D 'brick-wall' array of pancake-bonded dimers, b) crystal packing viewed in the direction [010] showing alternating layers of TCNQ$^{\delta-}$ anions (blue) and cations (red).

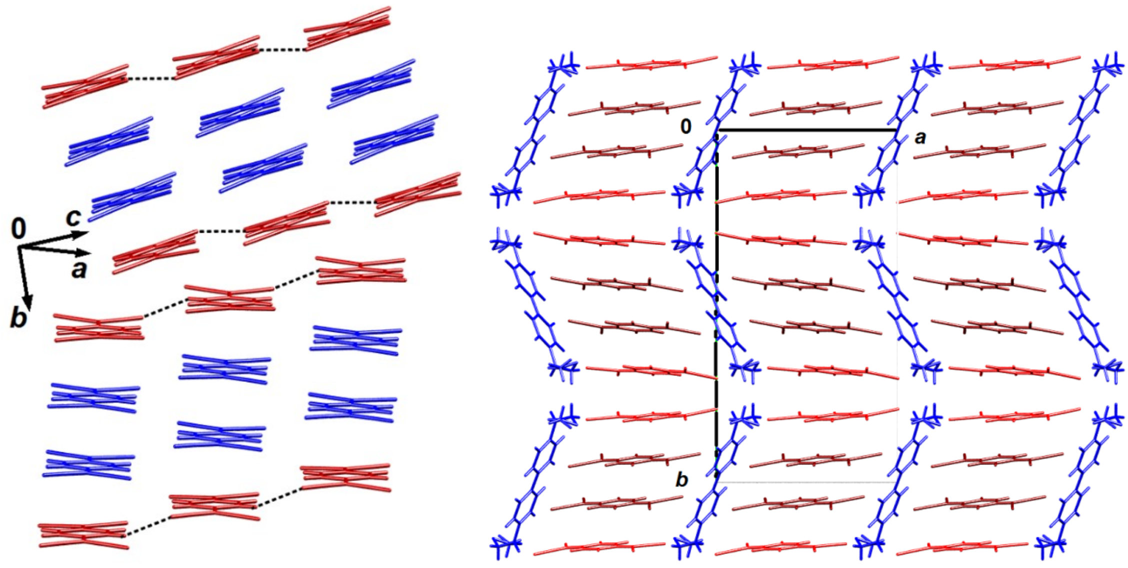

a) **b)**

Figure S12 Crystal packing of **9·(TCNQ)₄**: a) 2D herringbone array of pancake-bonded tetramers (anion A is red and B is blue), b) crystal packing viewed in the direction [010] showing alternating layers of TCNQ$^{\delta-}$ anions (red) and cations (blue).

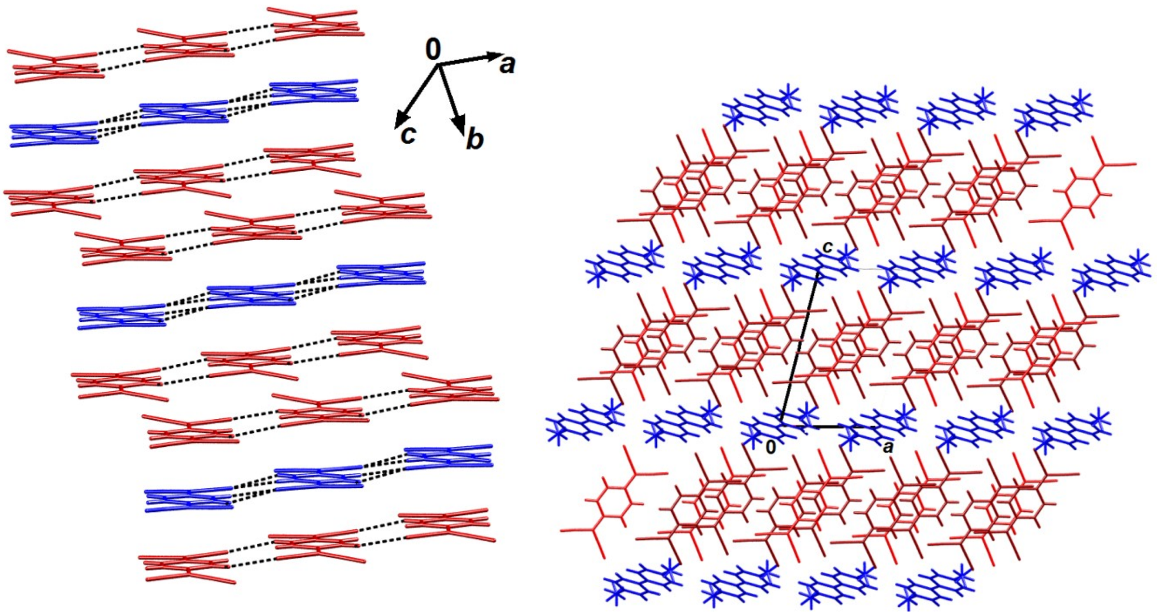

a) **b)**

Figure S13 Crystal packing of **10·(TCNQ)₃**: a) 2D 'brick-wall' array of pancake-bonded trimers (cation A is red and cation B is blue), b) crystal packing viewed in the direction [010] showing alternating layers of TCNQ$^{\delta-}$ anions (red) and cations (blue).

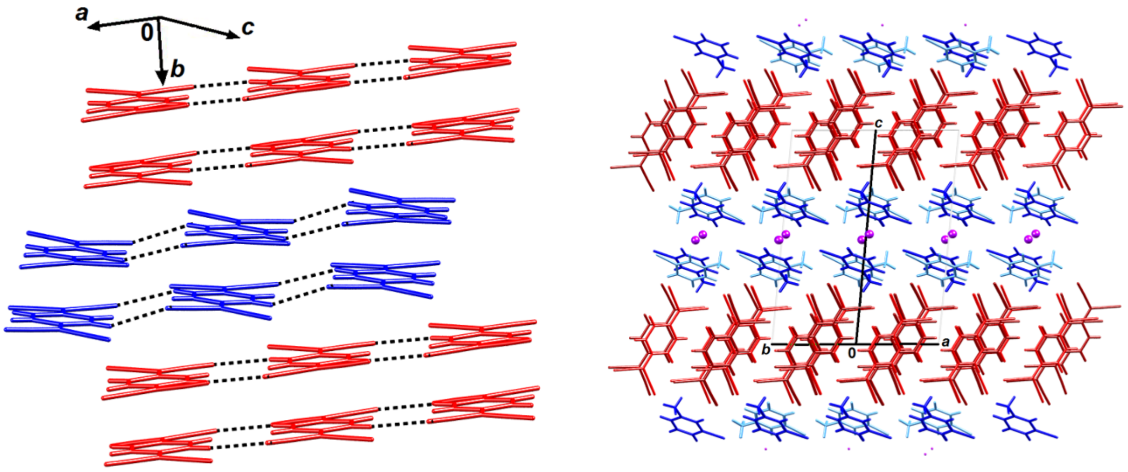

a) **b)**

Figure S14 Crystal packing of **11_2·I·(TCNQ)$_2$**: a) 2D ‘brick-wall’ array of pancake-bonded dimers (cation A is blue and cation B is red), b) crystal packing viewed in the direction [110] showing alternating layers of TCNQ$^{\delta-}$ anions (red), cations (blue) and iodide anions (purple, shown as spheres of arbitrary radii).

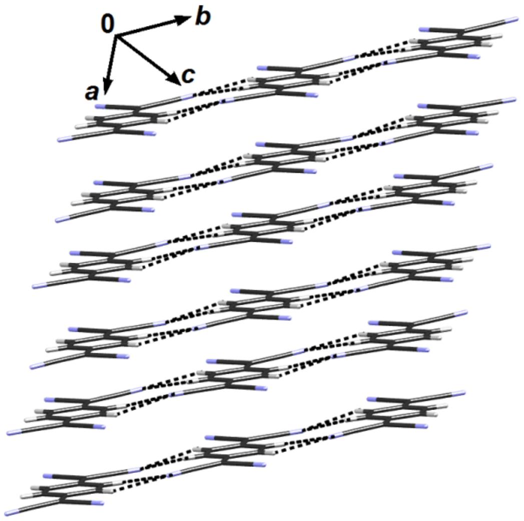

a)

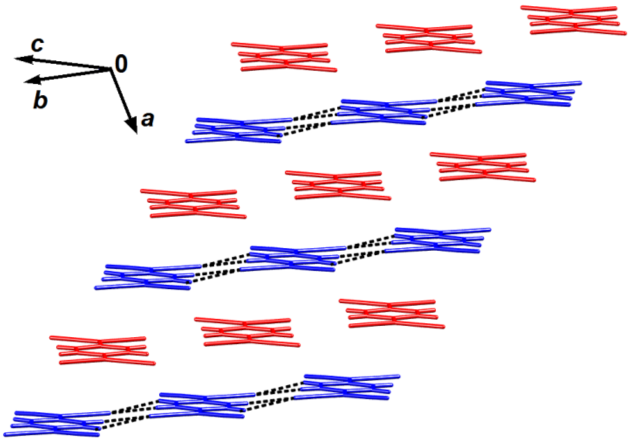

b)

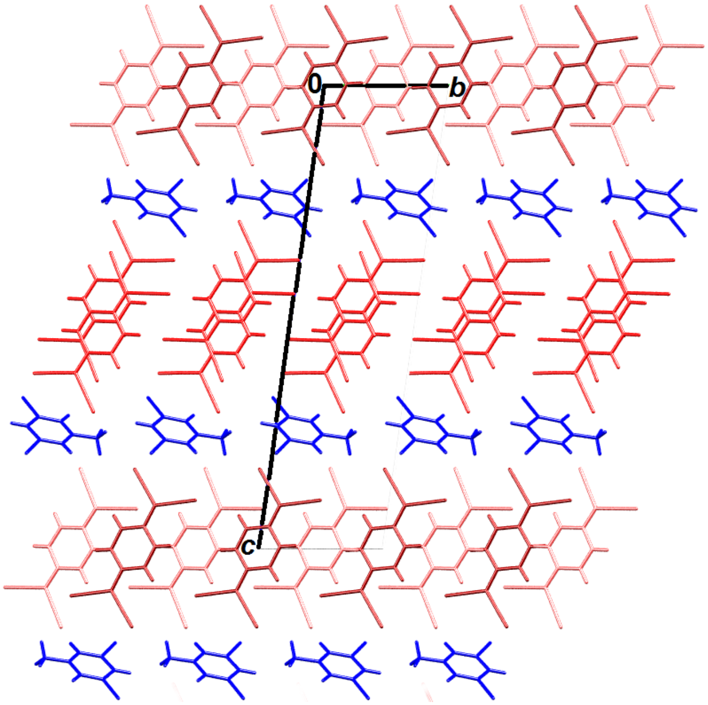

c)

Figure S15 Crystal packing of **12·(TCNQ)$_2$**: a) 2D ‘brick-wall’ array of pancake-bonded dimers of molecules A, b) stacks of equidistant radicals of molecules B and C (red and blue, respectively) and c) crystal packing viewed in the direction [100] showing alternating layers of TCNQ$^{\delta-}$ anions (A: bright red, B: dark red, C: pale red) and cations (blue).

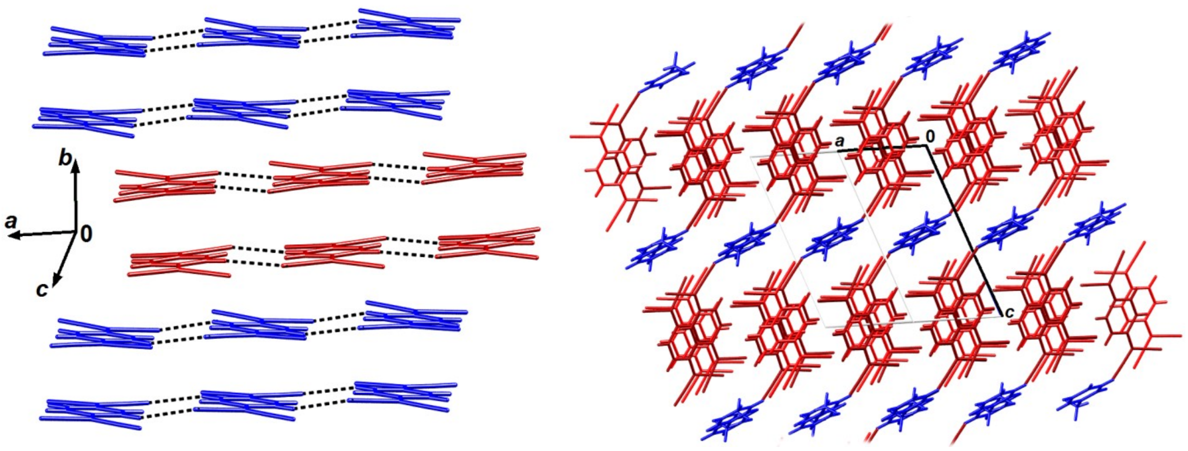

a) b)

Figure S16 Crystal packing of **13·(TCNQ)₂** (100 K): a) 2D 'brick-wall' array of pancake-bonded dimers (cation A is red and cation B is blue), b) crystal packing viewed in the direction [010] showing alternating layers of TCNQ$^{\delta-}$ anions (red) and *N*-MePy cations (blue). Crystal packing at RT is analogous, except that there is only one symmetry-inequivalent TCNQ$^{\delta-}$ radical and the cation is disordered about an inversion centre (as shown in Fig. S3).

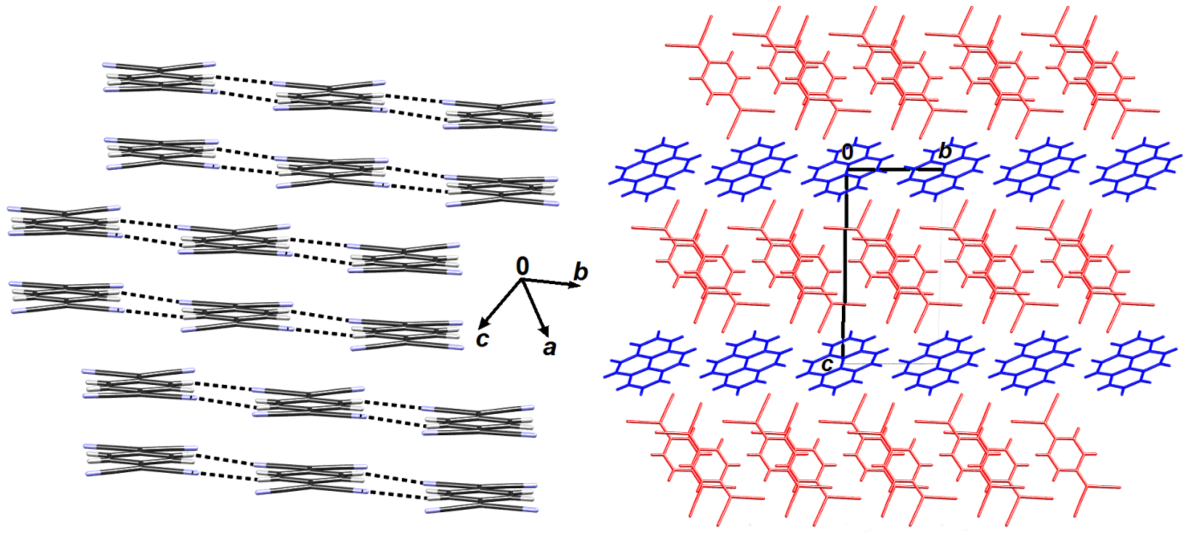

a) b)

Figure S17 Crystal packing of **14·(TCNQ)₂**: a) 2D 'brick-wall' array of pancake-bonded dimers, b) crystal packing viewed in the direction [100] showing alternating layers of TCNQ$^{\delta-}$ anions (red) and *N*-methylphenanthrolinium cations (blue).

S5. Powder EPR results

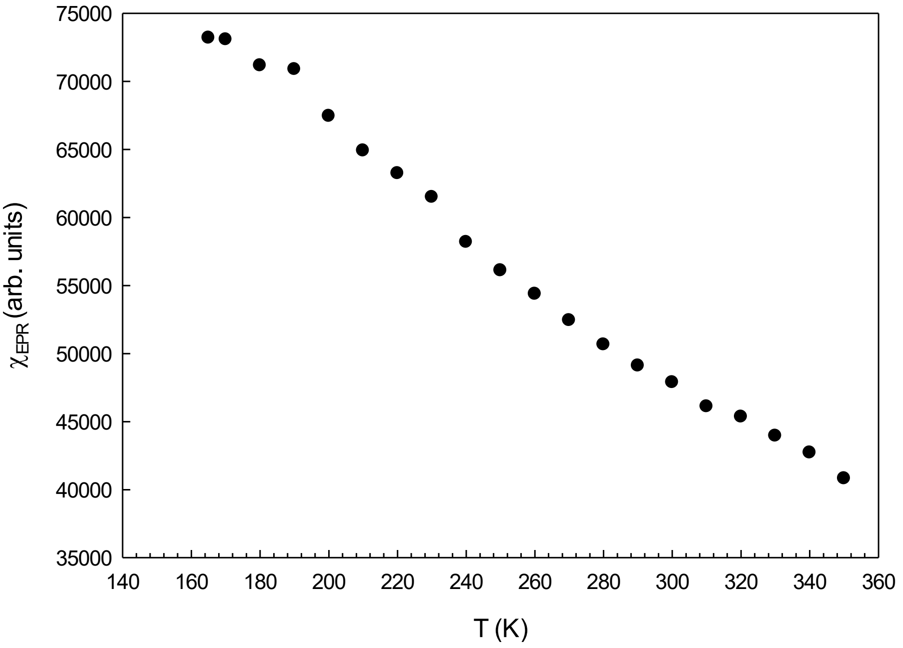

Figure S18 Temperature dependence of the total magnetic susceptibility, χ(EPR), of **1_2·Br·(TCNQ)$_2$** obtained by the double integration of the cumulative EPR spectra.

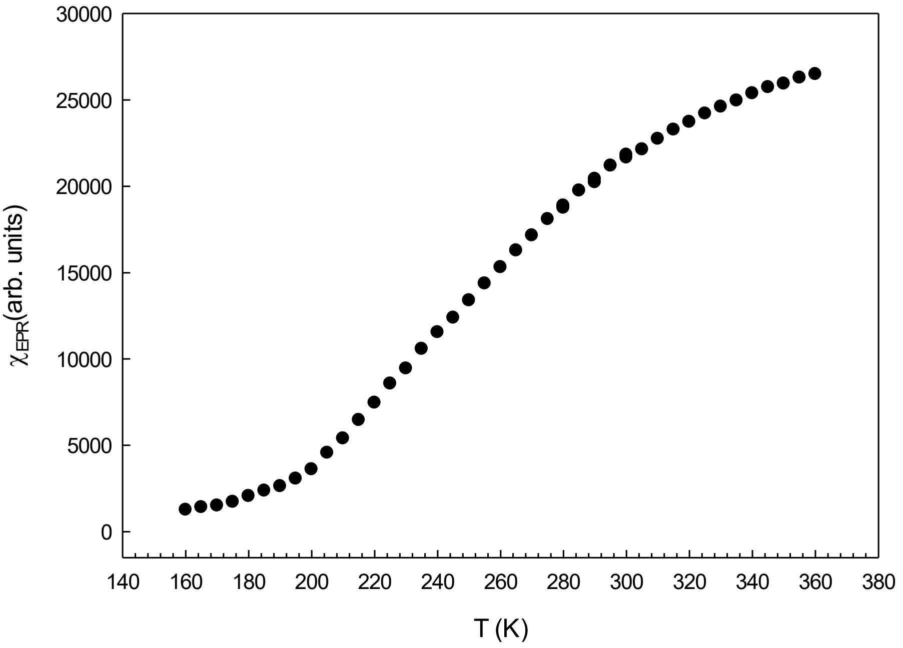

Figure S19 Temperature dependence of the total magnetic susceptibility, χ(EPR), of **4·(TCNQ)$_2$** obtained by the double integration of the cumulative EPR spectra.

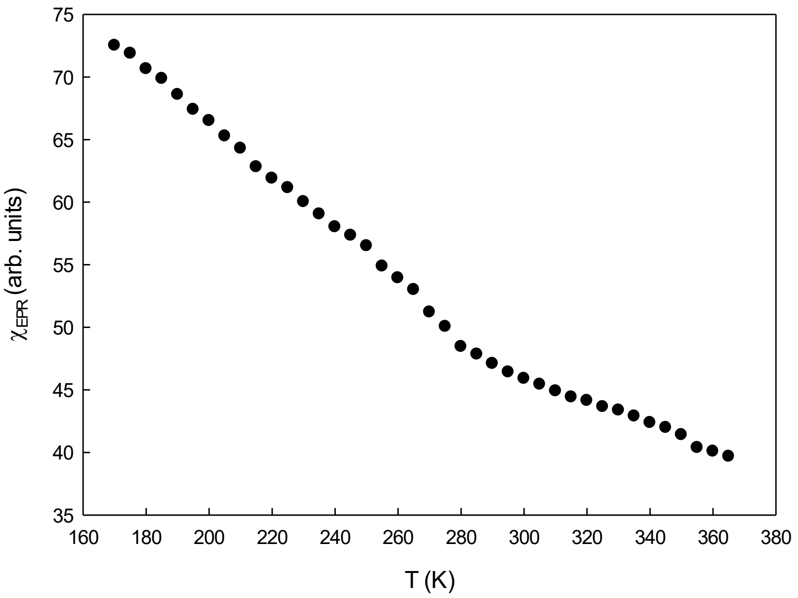

Figure S20 Temperature dependence of the magnetic susceptibility, χ(EPR), of **6·(TCNQ)$_2$** obtained by the double integration of the EPR spectra.

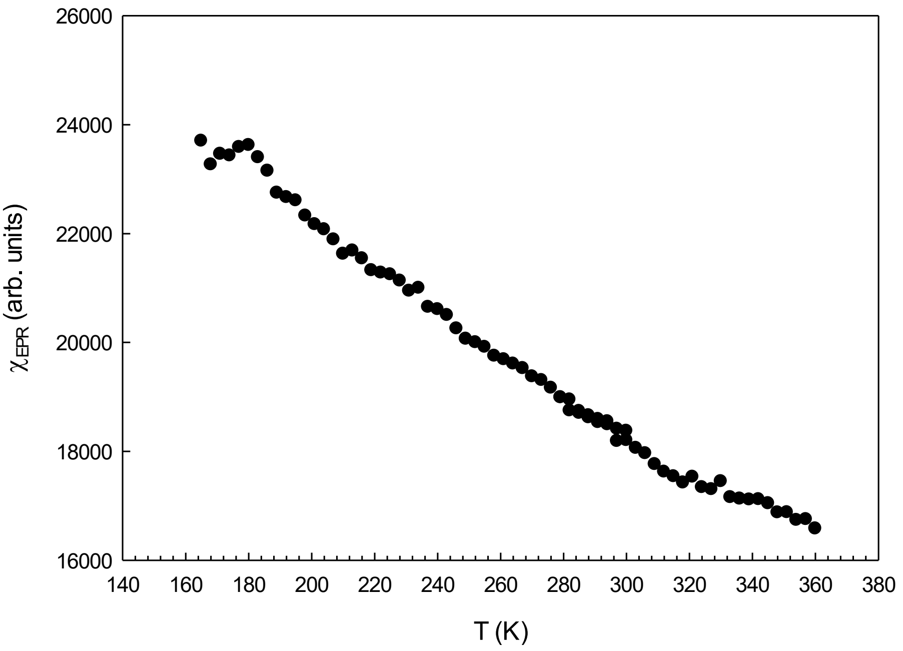

Figure S21 Temperature dependence of the magnetic susceptibility, χ(EPR), of **9·(TCNQ)$_4$** obtained by the double integration of the EPR spectra.

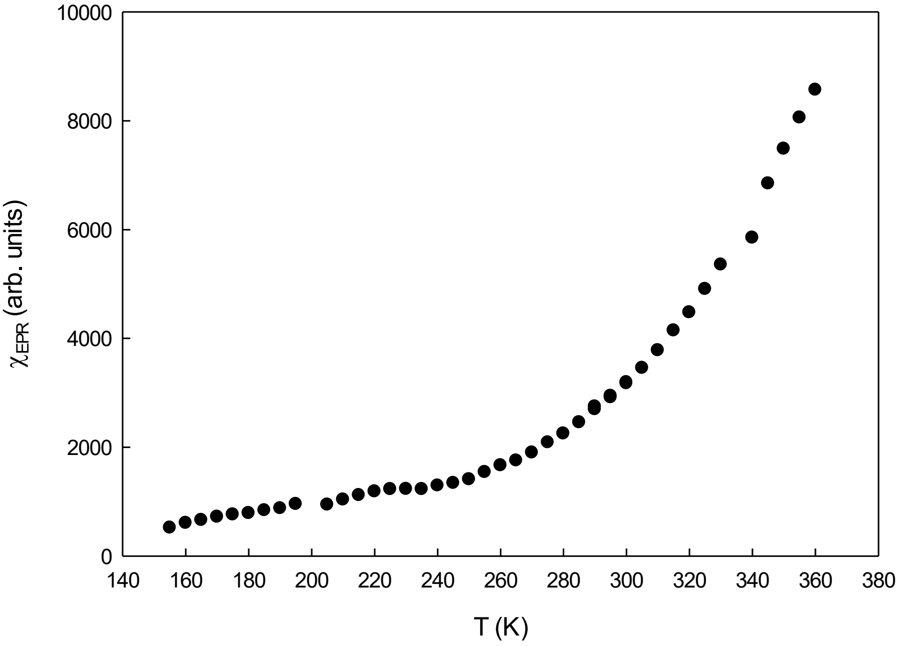

Figure S22 Temperature dependence of the total magnetic susceptibility, χ(EPR), of **10·(TCNQ)$_3$** obtained by the double integration of the cumulative EPR spectra.

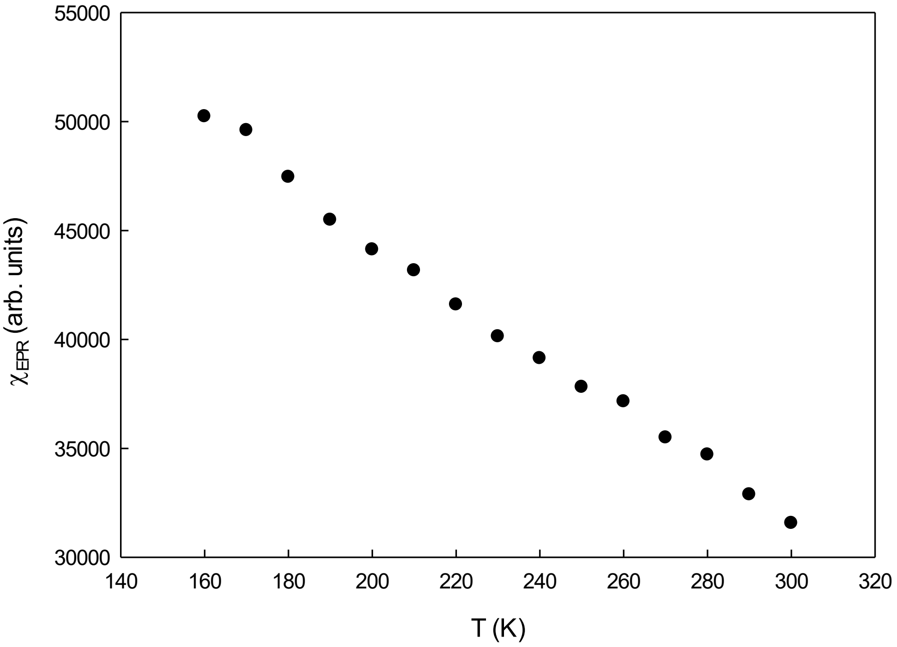

Figure S23 Temperature dependence of the total magnetic susceptibility, χ(EPR), of **12·(TCNQ)$_2$** obtained by the double integration of the cumulative EPR spectra shown.

S6. Single-crystal EPR results

Results obtained for samples **1_2·Br·(TCNQ)$_2$**, **4·(TCNQ)$_2$**, **6·(TCNQ)$_2$**, **9·(TCNQ)$_4$**, **10·(TCNQ)$_3$** and **12·(TCNQ)$_2$** are shown in Figs. S24-S29 for all three crystal orientations (red, green, blue). The orientation dependence of g-value was presented by down-pointing triangles, ▼, peak-to-peak linewidth, Γ, with squares, ■, inversed peak-to-peak intensity, I_{pp}^{-1}, with cricles, ●, and asymmetry with up-pointing triangles, ▲. It was assumed that we know the first rotation is set up by mounting the crystal onto the holder on the plane *ab* and with crystal axis *a* along the holder rod axis. Then two more orientations have been defined: $c^*=a\times b$ and $b'=c^*\times a$. The second rotation would have *b*' along the holder axis, and the third rotation c^*. A Cartesian coordinate system has been defined too to be attached with the crystal based on these three orientations: *a* has been assigned as *w*-axis, c^* as *u*-axis and *b*' as *v*-axis.

Sample **1_2·Br·(TCNQ)$_2$** has 2D brick-wall-like radical distribution in the plane (001) with the strongest interaction in the direction of the *a* axis. Plane bc is identified as the mounting plane. Three rotation axes are b, c' and a^*. Three rotation planes are a^*c', ba^* and bc. One could treat plane ba^* to be approximately the same as ab since angles α and β are both close to 90°. All three rotations show two peaks each; around the b axis (first) shows the smallest second peak. Rotations around a and b show 2D interactions and conductivity.

The relatively large linewidth changes in all three rotations have been observed. In all three rotations, two linewidth peaks, 90° apart, are observed. These two peaks have significantly different magnitudes in the first and third rotations yet are nearly equal in the second rotation. It can be considered the linewidth evolution in the first and third rotations is in accordance with a 2D spin system. The large variation of linewidths in the second rotation is still an open question. We can then identify the linewidth maximum of rotation 1 as the orientation *c*', at which the magnetic field becomes perpendicular to the ‘brick wall’, and the smaller, local maximum of linewidth as approximately the orientation a^*, at which the magnetic field is parallel to the brick wall. Similarly, the linewidth maximum of rotation 3 could again be identified as the orientation *c*’, at which the magnetic field becomes perpendicular to the brick wall, and the smaller, local maximum of linewidth as approximately the orientation *b*, at which the magnetic field is parallel to the brick wall.

Sample **4·(TCNQ)$_2$** exhibits the largest linewidth variations (0.05~0.3 mT) among all samples measured, which are observed in rotations 1 and 3. Rotation 2 exhibits less variation than the other two. Three rotation axes are a, b' and c^*. Three rotation planes are $b'c^*$, ac^* and ab. The results support 2D ‘brick wall’ of tetramers in the plane *ab* (001) and the strongest interaction in the [110] direction (i.e. between axes *a* and *b*).

Sample **6·(TCNQ)₂** is 2D 'brick wall' of dimers in the plane ab (001) with the strongest interaction (stacking) in direction a. Three rotation axes are b, a' and c^*. Three rotation planes are $a'c^*$, bc^* and ab. Line widths are relatively large ($0.2{\sim}0.3\ mT$) and not varying much in all three rotations. The single linewidth peak has been noticed in each rotation.

Sample **8·(TCNQ)₂** has brick wall-like distribution of the radicals in the *ab* plane, i.e. 2D arrangement of parallel dimer stacks, in the plane (001). The strongest interaction is in the b-axis direction. The crystal habitus is unknown; therefore, one cannot connect the rotation experiment with crystal structure directly. It can be noticed that rotation 2 exhibits the large/small linewidth peaks smilar to samples **12·(TCNQ)₂**, **1₂·Br·(TCNQ)₂** and **4·(TCNQ)₂** while rotations 1 and 3 seem to have no such feature.

Sample **9·(TCNQ)₄** is monoclinic, compared to the rest of the samples that are triclinic and has tetramers arranged in a 2D herringbone pattern, in the plane (100). The radicals stack in a tilted fashion along the b-axis, tile along the c-axis and finally the "rippled wall" repeats itself along the a-axis. The strongest interaction is approximately along axis *b*. Three rotation axes are a, b' and c^*. Three rotation planes are $b'c^*$, ac^* and ab. Since the crystal is monoclinic and with $\beta \approx 93°$, we could approximate $b' \approx b$ and $c^* \approx c$. In the rotation 1 the sample exhibits large/small linewidth peaks like similar to samples **12·(TCNQ)₂**, **1₂·Br·(TCNQ)₂** and **4·(TCNQ)₂**, while rotation 3 has a strong linewidth peak with a magnitude about the same as rotation 1, yet no small secondary peak. The change of the linewidth is less accentuated in the case of rotation 2.

Sample **12·(TCNQ)₂** has 2D brick-wall-like distribution of the radicals in the *ab* plane, which is also identified as the mounting plane. Three rotation axes are *a*, *b*' and *c**. Three rotation planes are *b*'*c**, *ac** and *ab*. Linewidths are small (<0.1 mT) at all orientations. Large linewidth changes in the first and second rotations have been observed. Both rotations develop one large and one small linewidth maximum which are approximately 90° apart from each other. The third rotation exhibits much smaller linewidth changes than the other two. This behaviour as in accordance with a 2D spin system: 2D arrangement (dimers arranged as a 'brick wall' and equidistant stacks) in the plane (001); stacking in two directions, one in the direction of the a axis, the other in the [110] direction (i.e. along the bisector of the *a* and *b* axes). The repeatable line asymmetries in the rotation 1 and 3 of sample **12·(TCNQ)₂** have been observed which is unique among all samples studied. Taking into account that the conductivity is small, the asymmetry of other samples can be considered more likely induced by imperfections. EPR parameters obtained by simulation have been presented in Table 6.

Table S3 The fitting parameters of peak-to peak line width, Γ, for each rotation extracted from Eq. (4) shown in Figs. S24-S29 by down-pointing triangle,▼

Sample	rotation	$A\ (G)$	$B\ (G)$	$C\ (G)$	$\alpha\ (°)$
	1	-0.0113	0.4916	2.6217	177.35
**6·(TCNQ)$_2$**	2	-0.0163	0.6150	2.2285	64.01
	3	-0.0018	0.7660	2.3117	171.55
	1	0.1005	-0.0255	0.4055	28.49
**12·(TCNQ)$_2$**	2	0.1115	-0.0408	0.3642	148.25
	3	0.0125	0.0170	0.4695	179.96
	1	0.1992	0.3704	1.0950	66.93
**1·Br·(TCNQ)$_2$**	2	0.7721	-2.2950	1.6734	136.52
	3	0.2556	-0.1996	1.8091	93.01
	1	0.9900	-2.0890	1..3047	52.31
**4·(TCNQ)$_2$**	2	0.1847	0.6089	0.6784	126.42
	3	0.5385	0.4456	0.4910	59.27
	1	0.0099	0.1820	0.3622	132.17
**8·(TCNQ)$_2$**	2	0.0746	0.1077	0.2971	109.25
	3	0.0112	0.1441	0.3623	120.37
	1	0.2429	-0.4737	0.4824	4.34
**9·(TCNQ)$_4$**	2	0.0363	0.3583	0.2148	102.37
	3	0.0900	0.4390	0.1817	124.03

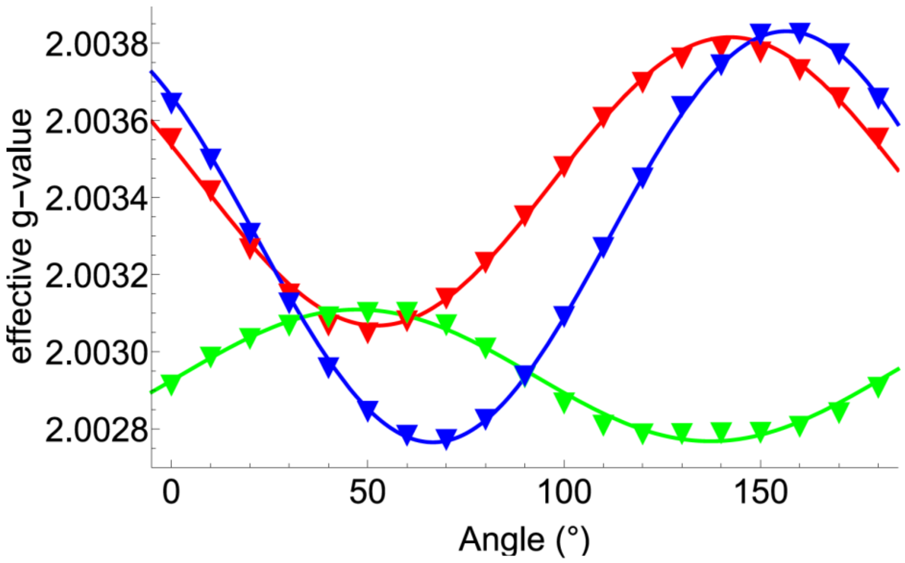
2.0038
2.0036
2.0034
2.0032
2.0030
2.0028
effective g−value
0
50
100
150
Angle (°)

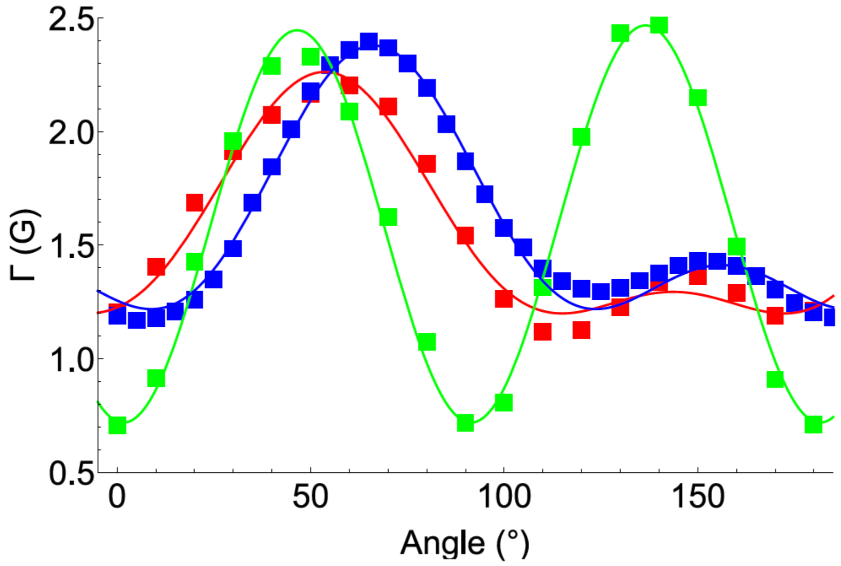
2.5
2.0
1.5
1.0
0.5
Γ (G)
0
50
100
150
Angle (°)

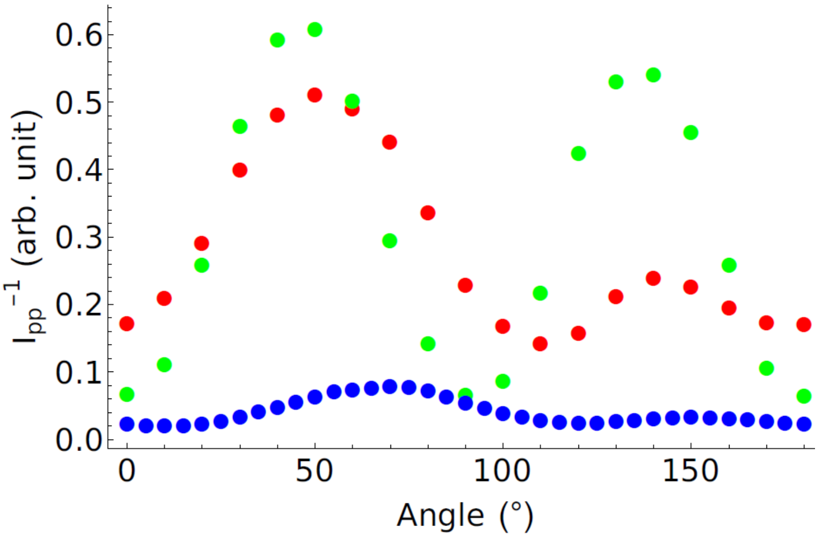
0.6
0.5
0.4
0.3
0.2
0.1
0.0
I_{pp}^{-1} (arb. unit)
0
50
100
150
Angle (°)

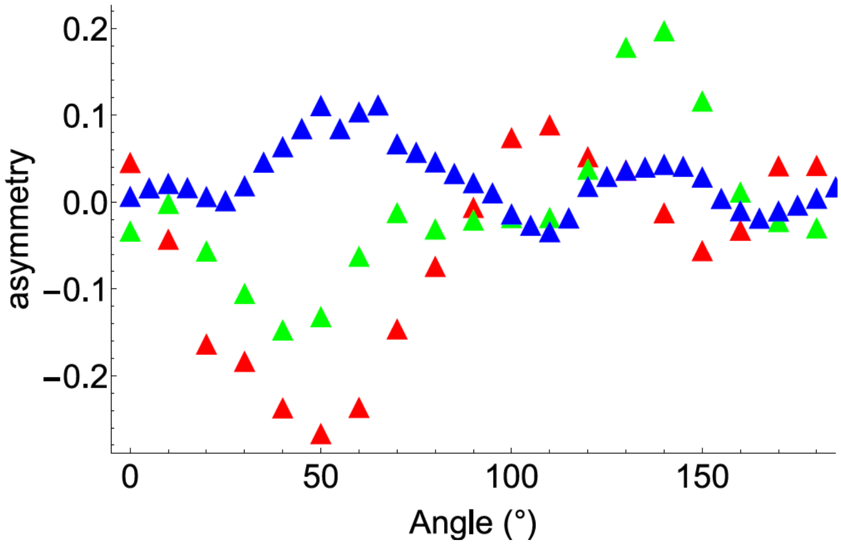

Figure S24 Single-crystal EPR results for the compound **1_2·Br·(TCNQ)$_2$**. The orientation dependence of *g*-value is shown as by down-pointing triangles (▼), peak-to-peak linewidth, *Γ*, as squares (■), inversed peak-to-peak intensity, I_{pp}^{-1}, as circles (●) and asymmetry as up-pointing triangles (▲). Three rotation axes are *b*, *c*' and *a**, which are shown as blue, red and green colours, respectively.

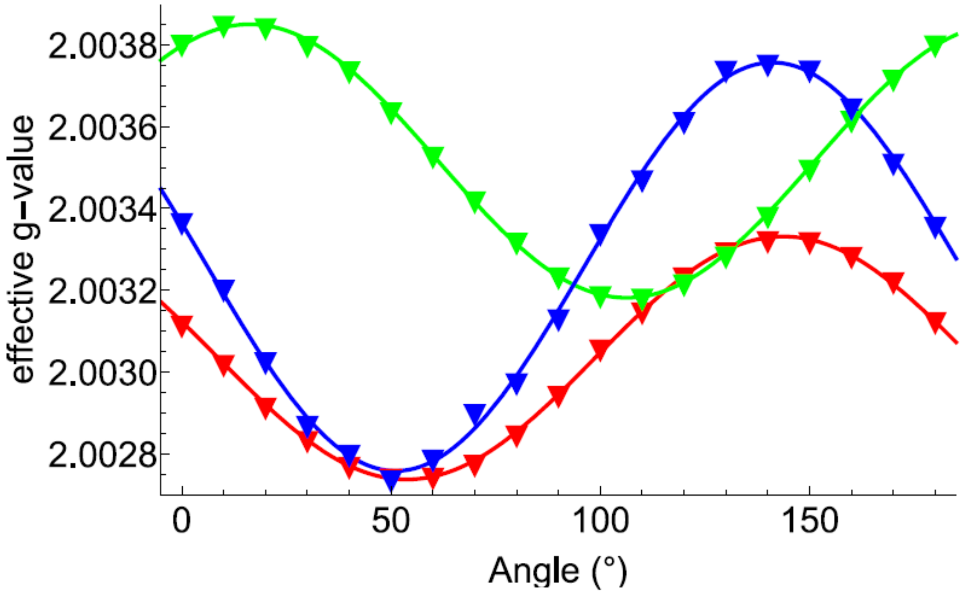

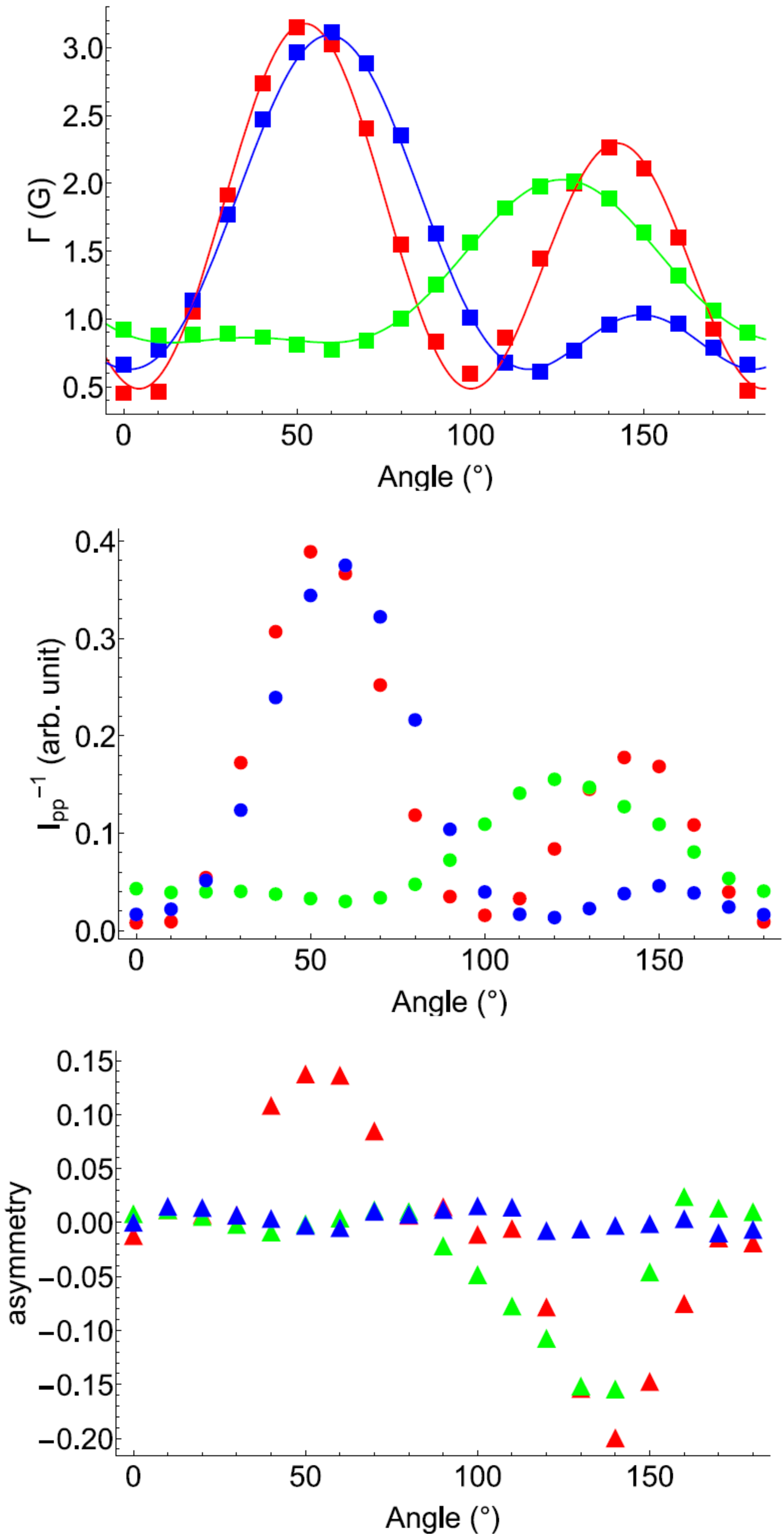
Γ (G)
3.0
2.5
2.0
1.5
1.0
0.5
0
50
100
150
Angle (°)
I_{pp}^{-1} (arb. unit)
0.4
0.3
0.2
0.1
0.0
0
50
100
150
Angle (°)
asymmetry
0.15
0.10
0.05
0.00
−0.05
−0.10
−0.15
−0.20
0
50
100
150
Angle (°)

Figure S25 Single-crystal EPR results for the compound **4·(TCNQ)₂**. The orientation dependence of g-value is shown as by down-pointing triangles (▼), peak-to-peak linewidth, Γ, as squares (■), inversed peak-to-peak intensity, I_{pp}^{-1}, as circles (●) and asymmetry as up-pointing triangles (▲). Three rotation axes are b, c' and a^*, which are shown as blue, red and green colours, respectively.

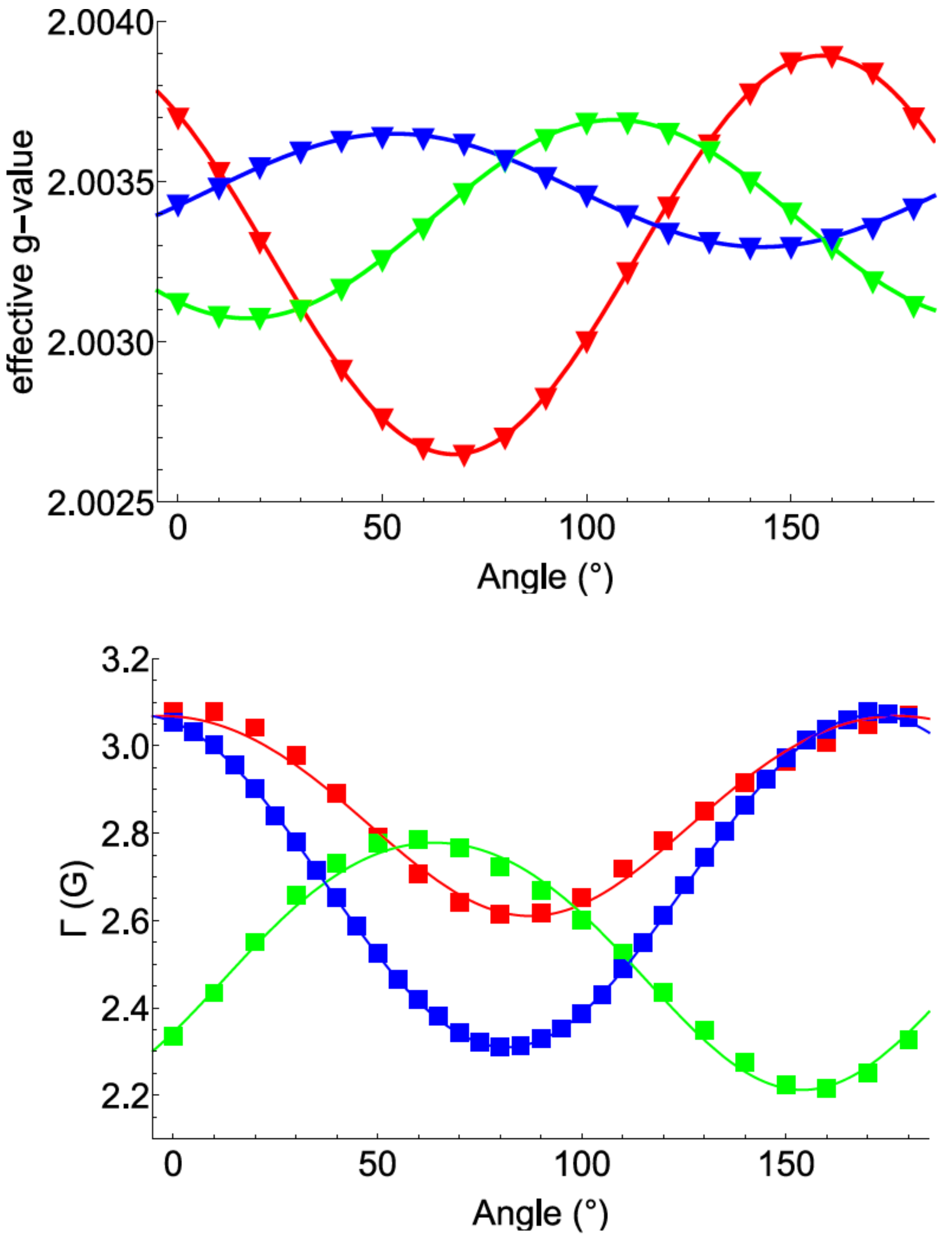

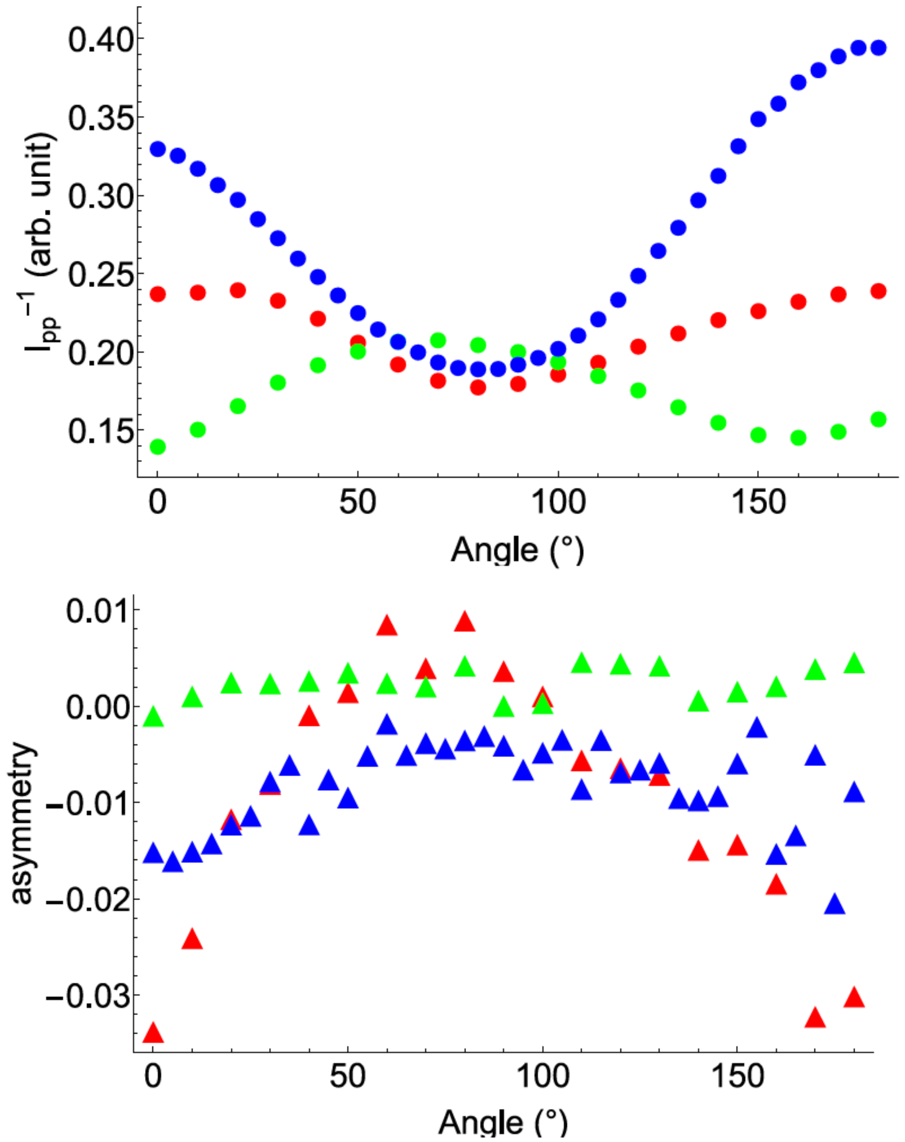

Figure S26 Single-crystal EPR results for the compound **6·(TCNQ)₂**. The orientation dependence of *g*-value is shown as by down-pointing triangles (▼), peak-to-peak linewidth, Γ, as squares (■), inversed peak-to-peak intensity, I_{pp}^{-1}, as circles (●) and asymmetry as up-pointing triangles (▲). Three rotation axes are b, c' and a^{*}, which are shown as blue, red and green colours, respectively.

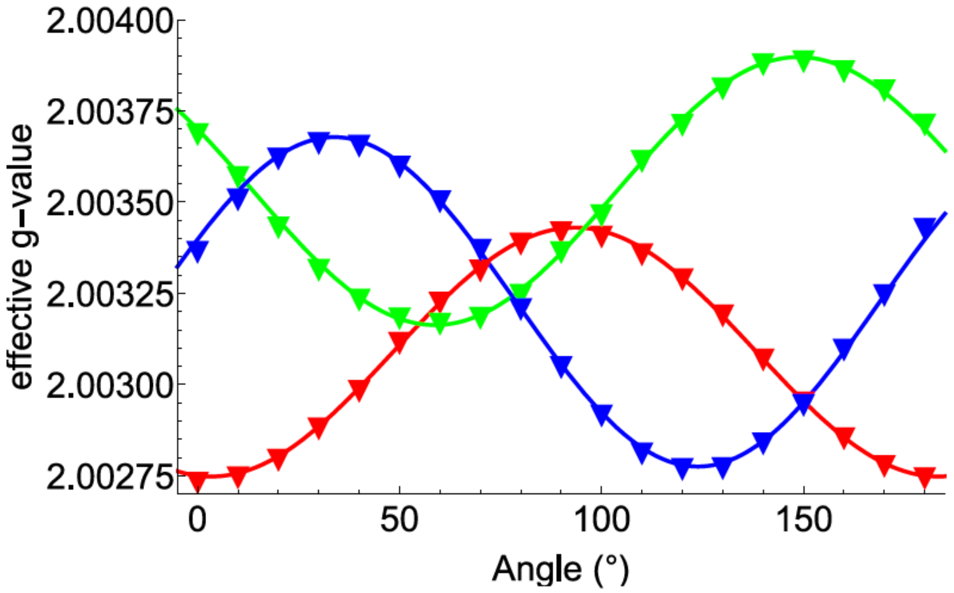
2.00400
2.00375
2.00350
2.00325
2.00300
2.00275
effective g−value
0
50
100
150
Angle (°)

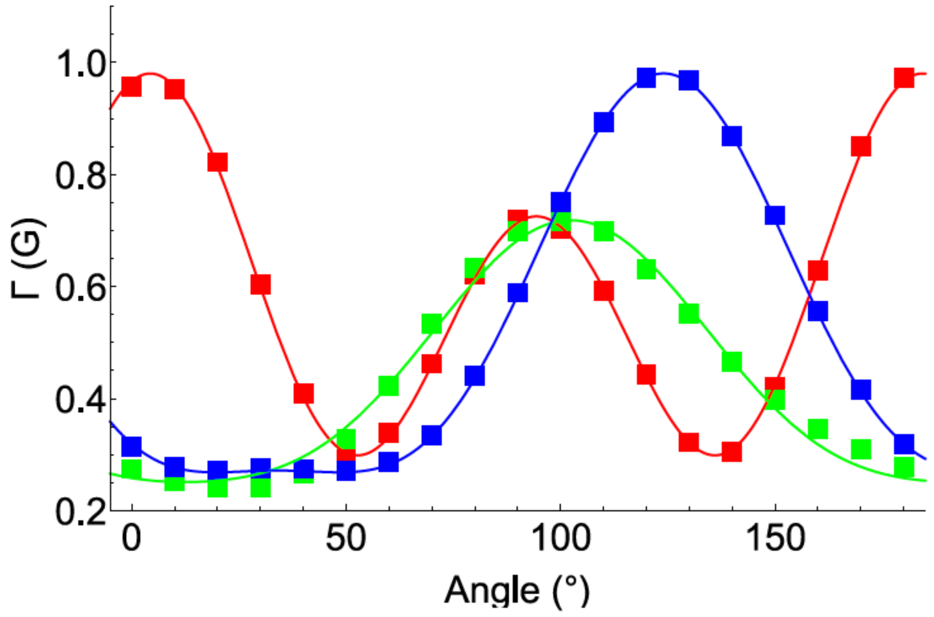
1.0
0.8
0.6
0.4
0.2
Γ (G)
0
50
100
150
Angle (°)

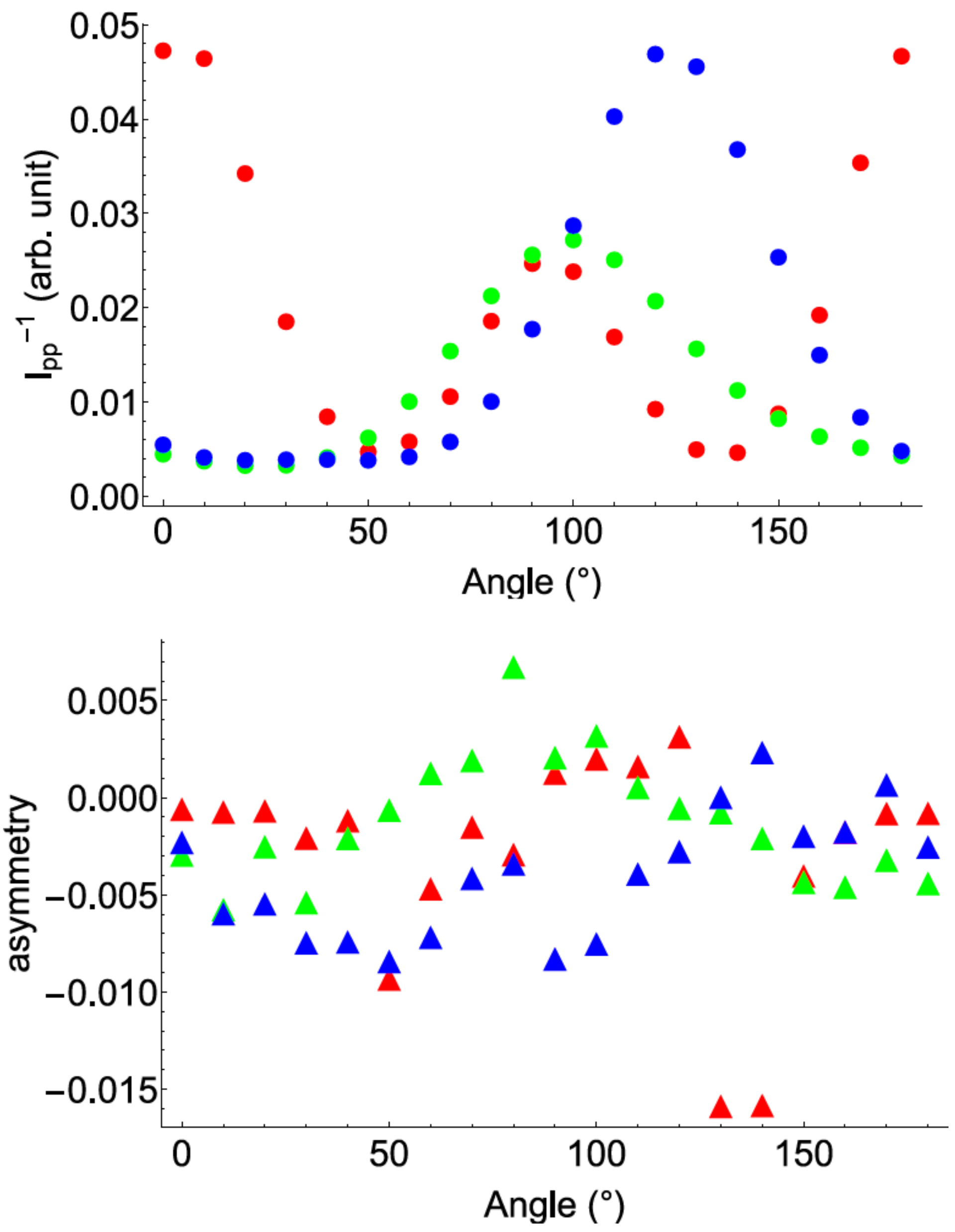

Figure S27 Single-crystal EPR results for the compound **8·(TCNQ)₂**. The orientation dependence of *g*-value is shown as by down-pointing triangles (▼), peak-to-peak linewidth, *Γ*, as squares (■), inversed peak-to-peak intensity, I_{pp}^{-1}, as circles (●) and asymmetry as up-pointing triangles (▲). Three rotation axes are *b*, *c*' and *a**, which are shown as blue, red and green colours, respectively.

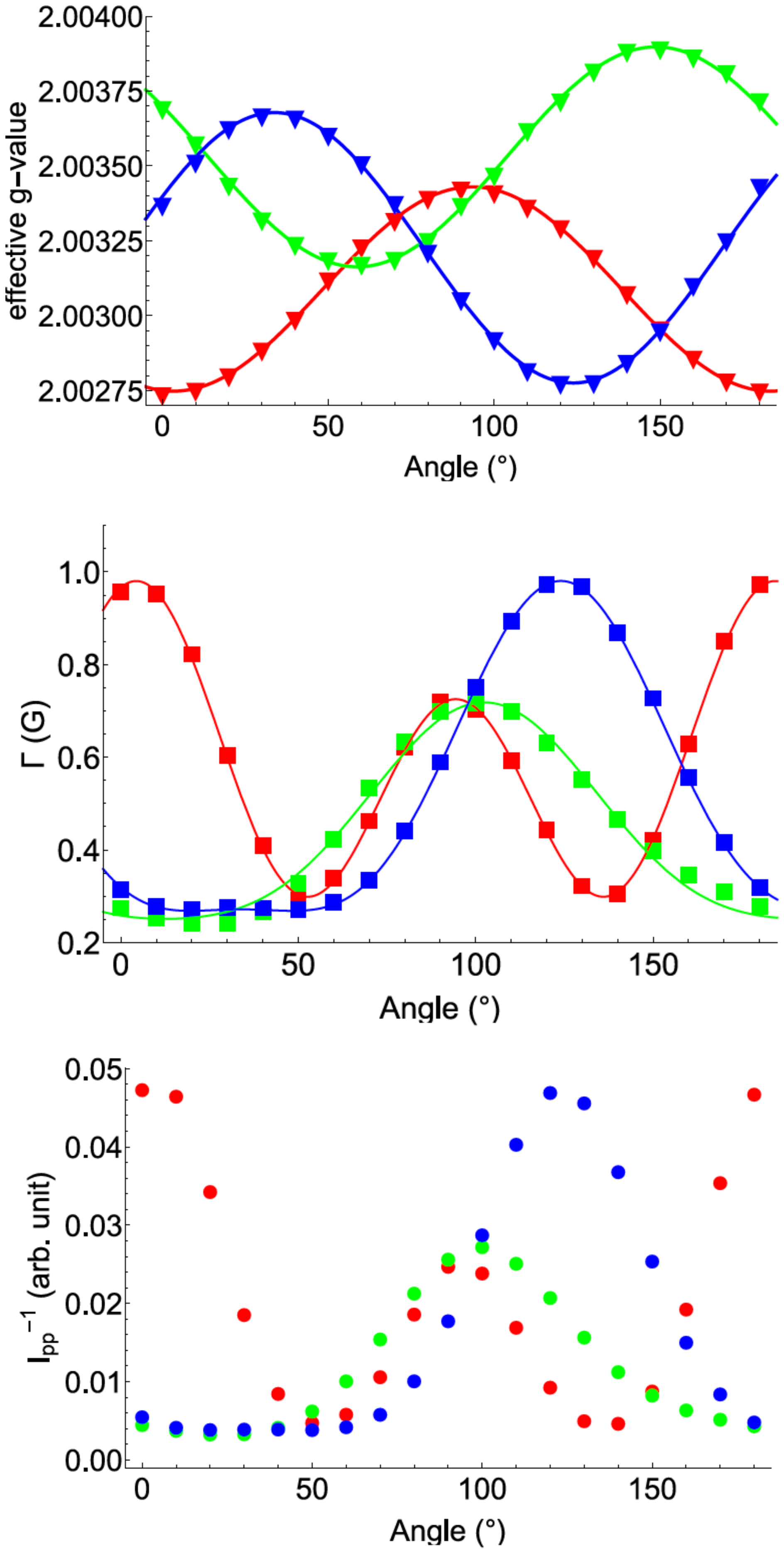

effective g−value
2.00400
2.00375
2.00350
2.00325
2.00300
2.00275
0
50
100
150
Angle (°)
Γ (G)
1.0
0.8
0.6
0.4
0.2
0
50
100
150
Angle (°)
I_{pp}^{-1} (arb. unit)
0.05
0.04
0.03
0.02
0.01
0.00
0
50
100
150
Angle (°)

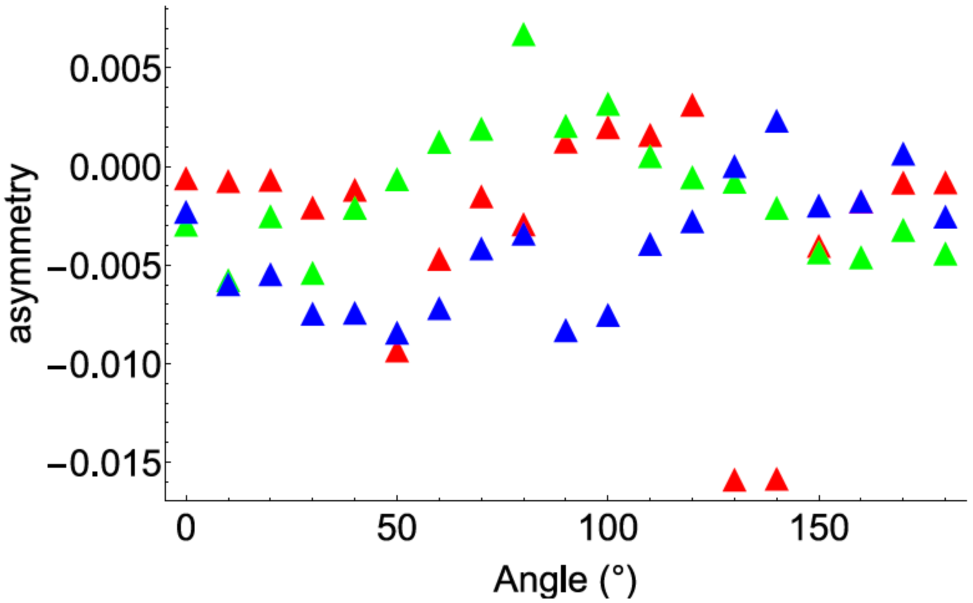

Figure S28 Single-crystal EPR results for the compound **9·(TCNQ)₄**. The orientation dependence of *g*-value is shown as by down-pointing triangles (▼), peak-to-peak linewidth, *Γ*, as squares (■), inversed peak-to-peak intensity, I_{pp}^{-1}, as circles (●) and asymmetry as up-pointing triangles (▲). Three rotation axes are b, c' and a^*, which are shown as blue, red and green colours, respectively.

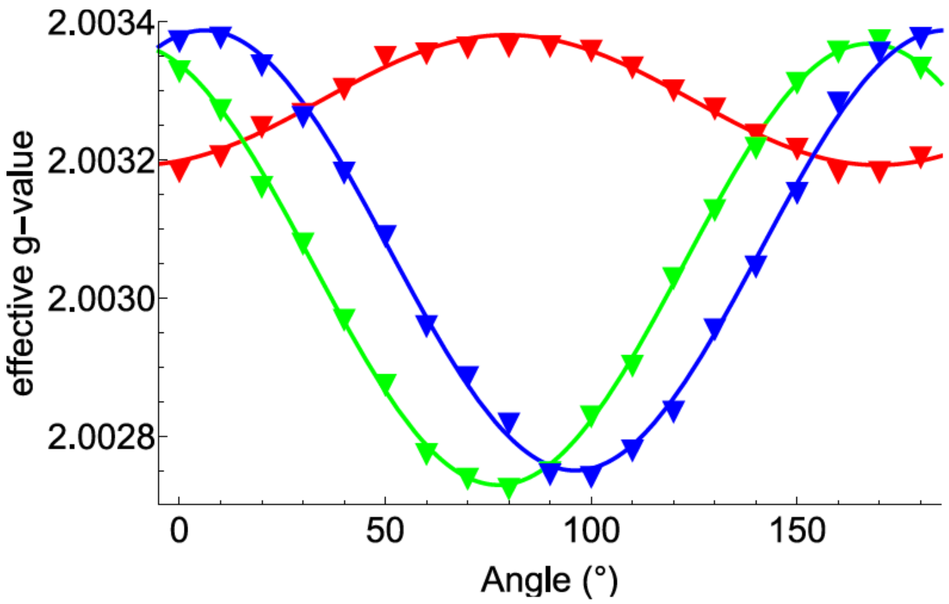

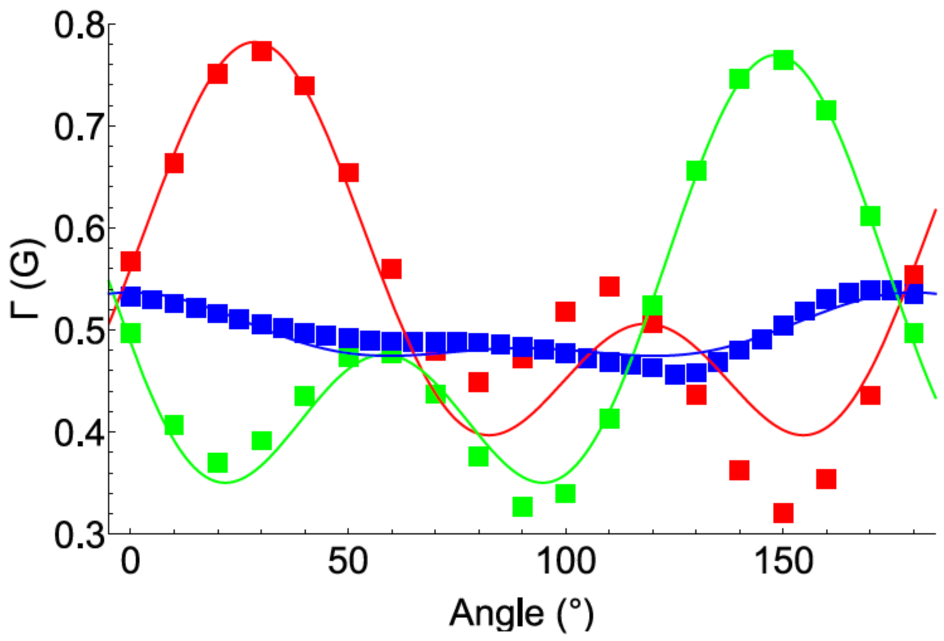
0.8
0.7
0.6
0.5
0.4
0.3
Γ (G)
0
50
100
150
Angle (°)

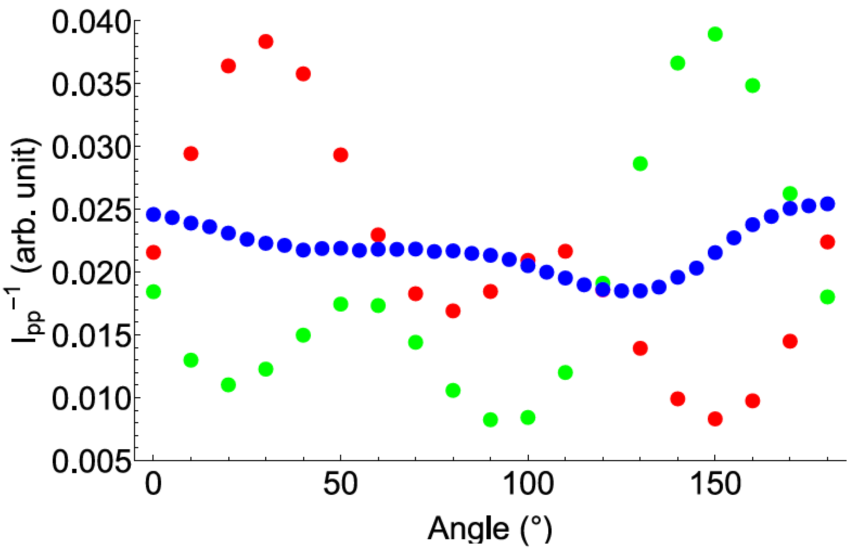
0.040
0.035
0.030
0.025
0.020
0.015
0.010
0.005
I_{pp}^{-1} (arb. unit)
0
50
100
150
Angle (°)

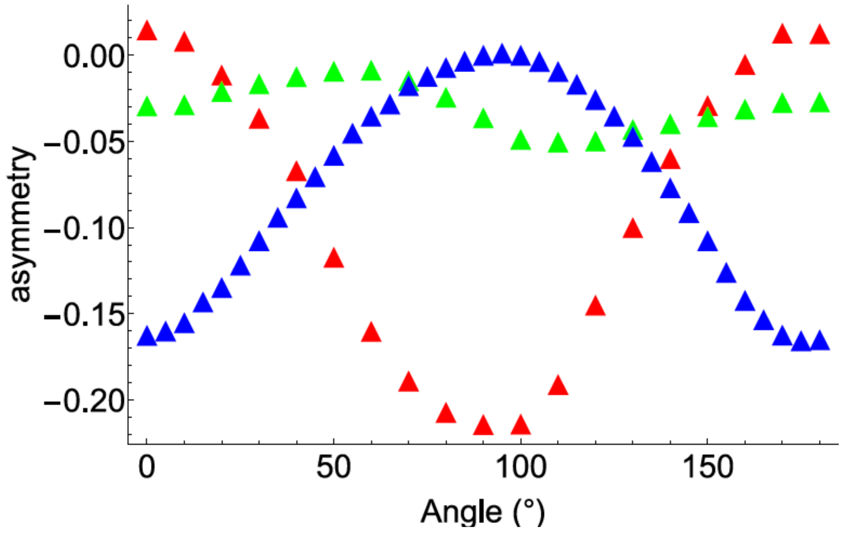
0.00
−0.05
−0.10
−0.15
−0.20
asymmetry
0
50
100
150
Angle (°)

Figure S29 Single-crystal EPR results for the compound **12·(TCNQ)₂**. The orientation dependence of g-value is shown as by down-pointing triangles (▼), peak-to-peak linewidth, Γ, as squares (■), inversed peak-to-peak intensity, I_{pp}^{-1}, as circles (●) and asymmetry as up-pointing triangles (▲). Three rotation axes are b, c' and a^*, which are shown as blue, red and green colours, respectively.

b-axis

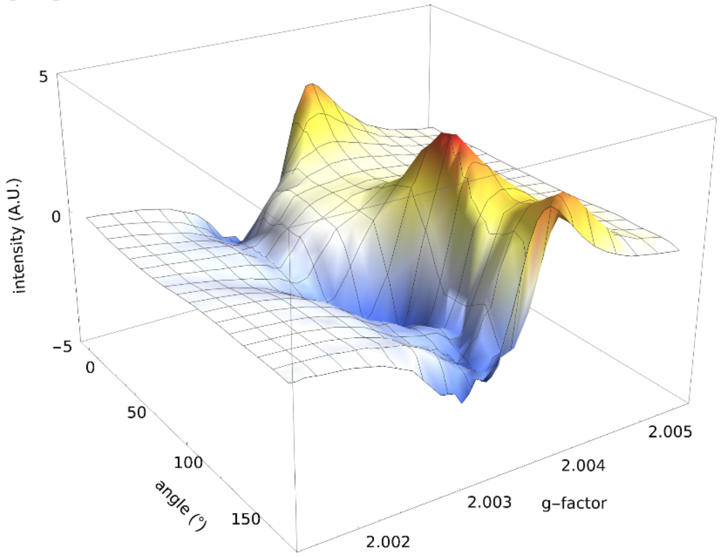

c'-axis

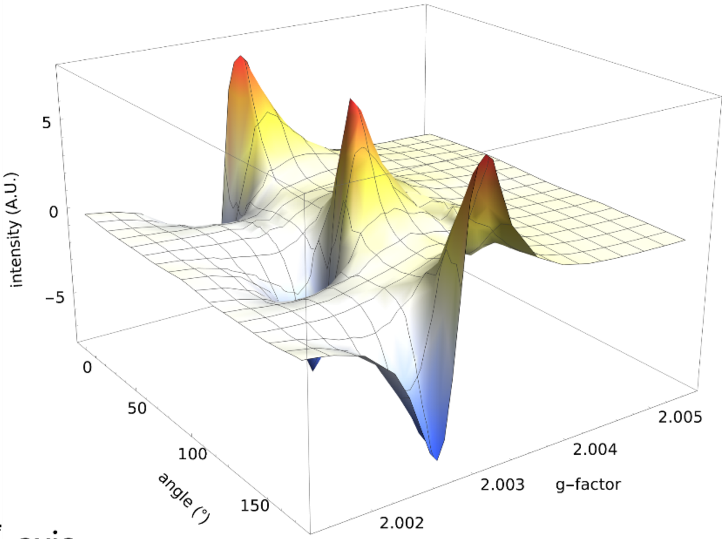

a*-axis

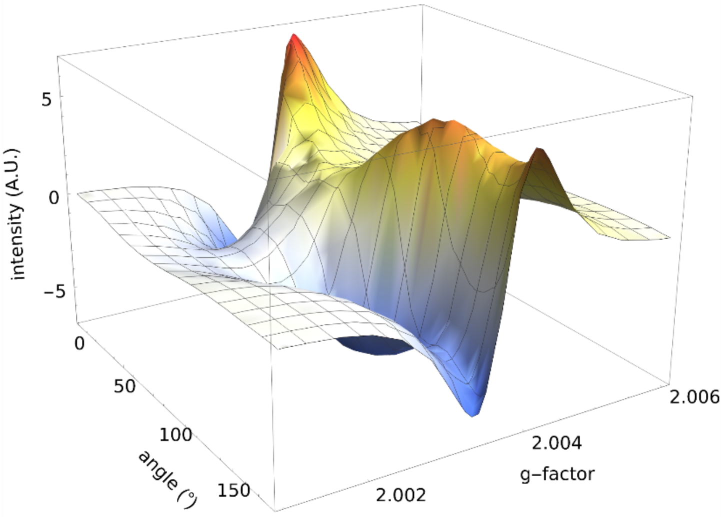

Figure S30 Angular dependence of the EPR spectra, recorded at RT, for **1_2·Br·(TCNQ)$_2$** single crystal.

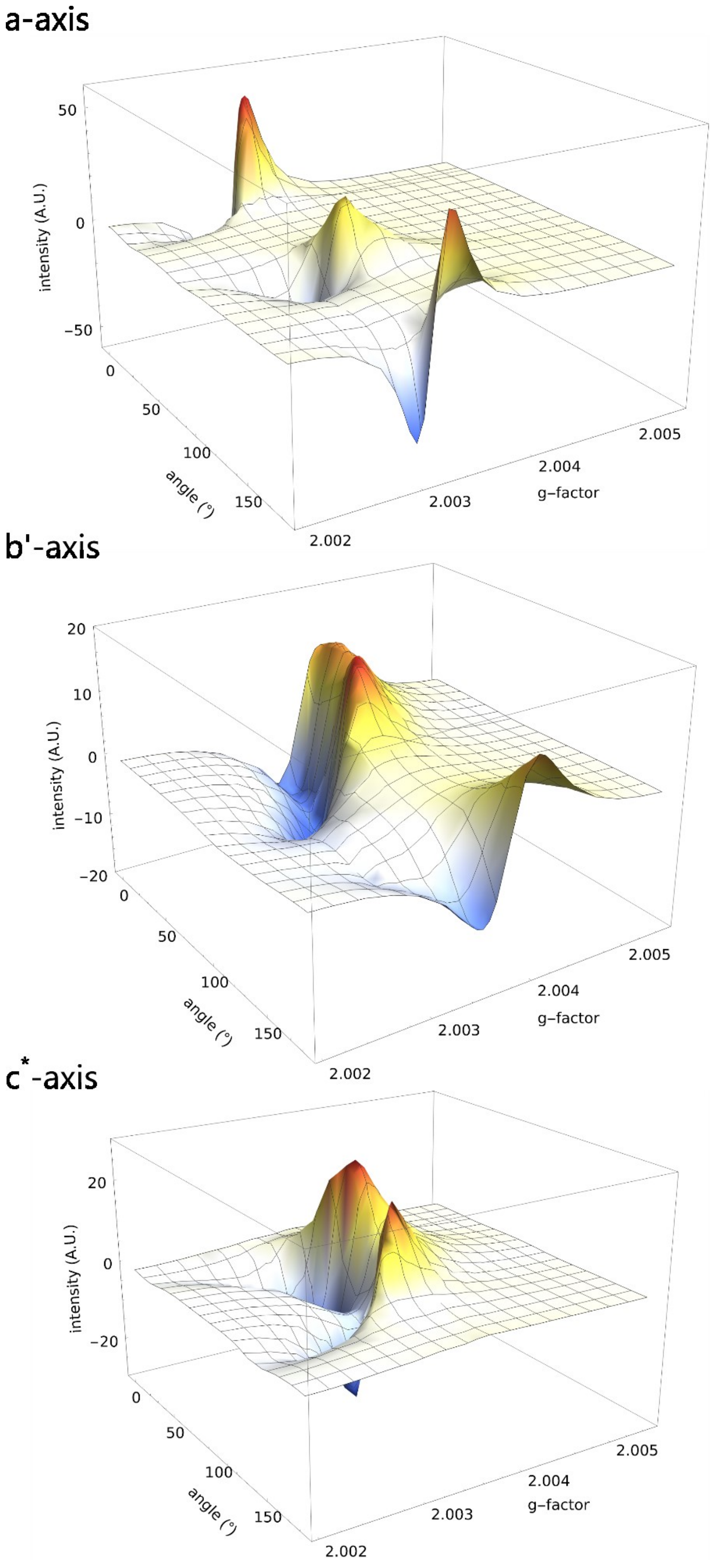

Figure S31 Angular dependence of the EPR spectra, recorded at RT, for **4·(TCNQ)$_2$** single crystal.

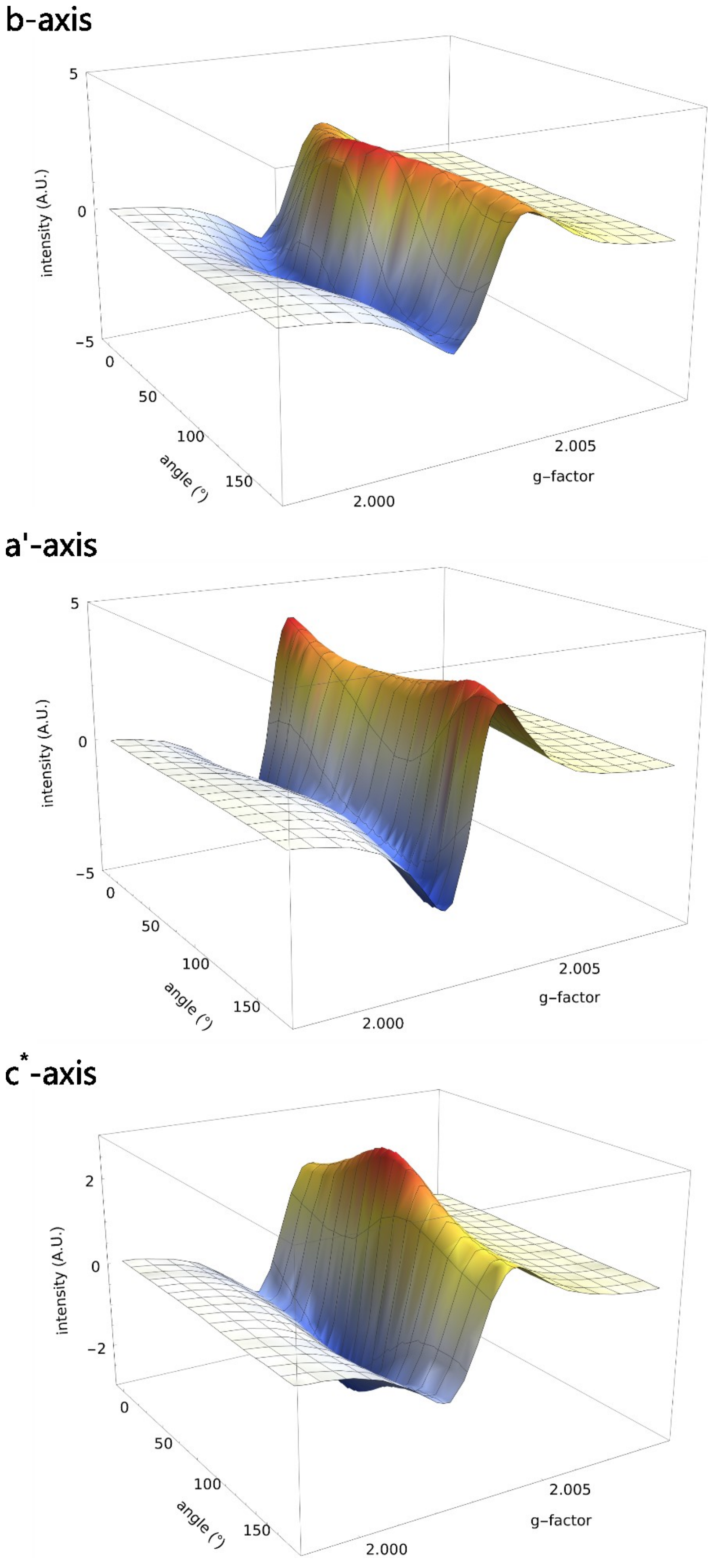

Figure S32 Angular dependence of the EPR spectra, recorded at RT, for **6·(TCNQ)$_2$** single crystal.

1

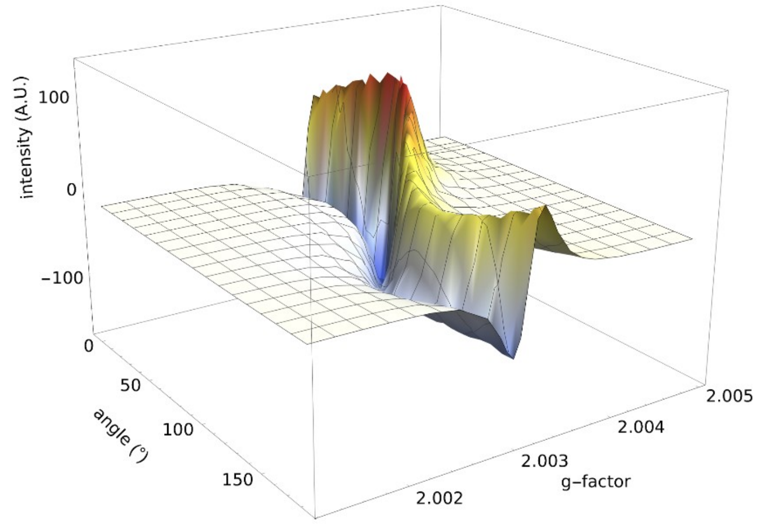

2

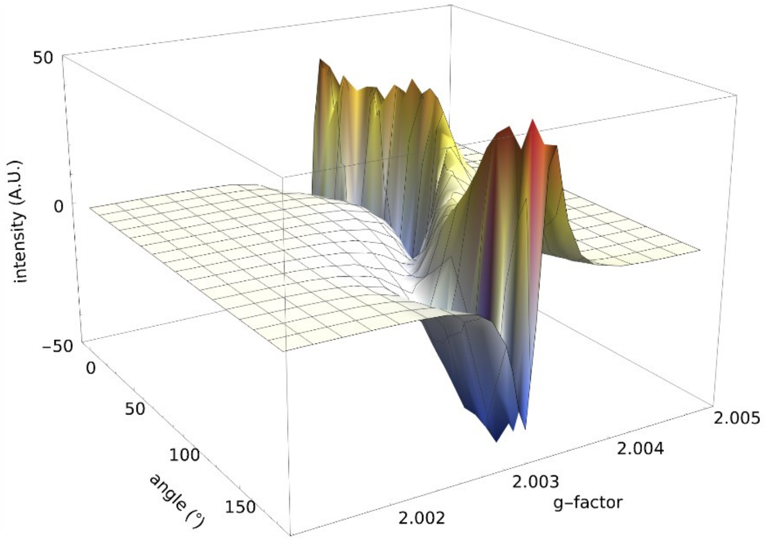

3

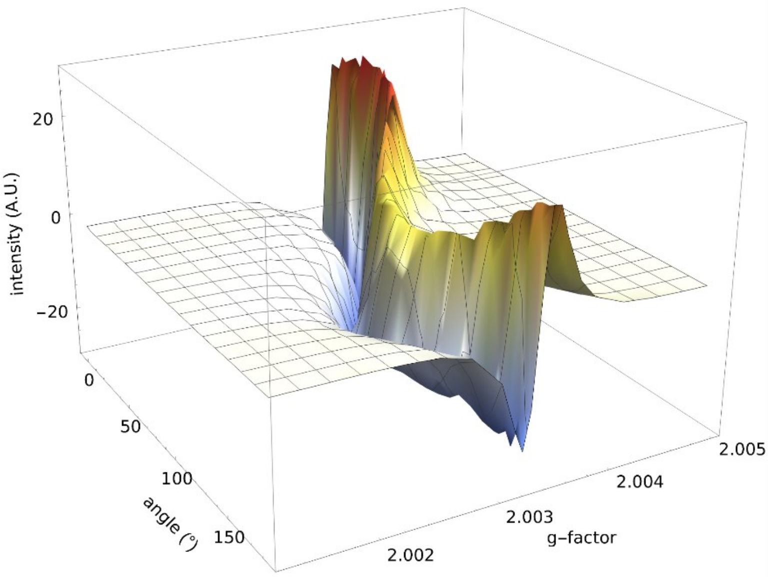

Figure S33 Angular dependence of the EPR spectra, recorded at RT, for **8·(TCNQ)$_2$** single crystal.

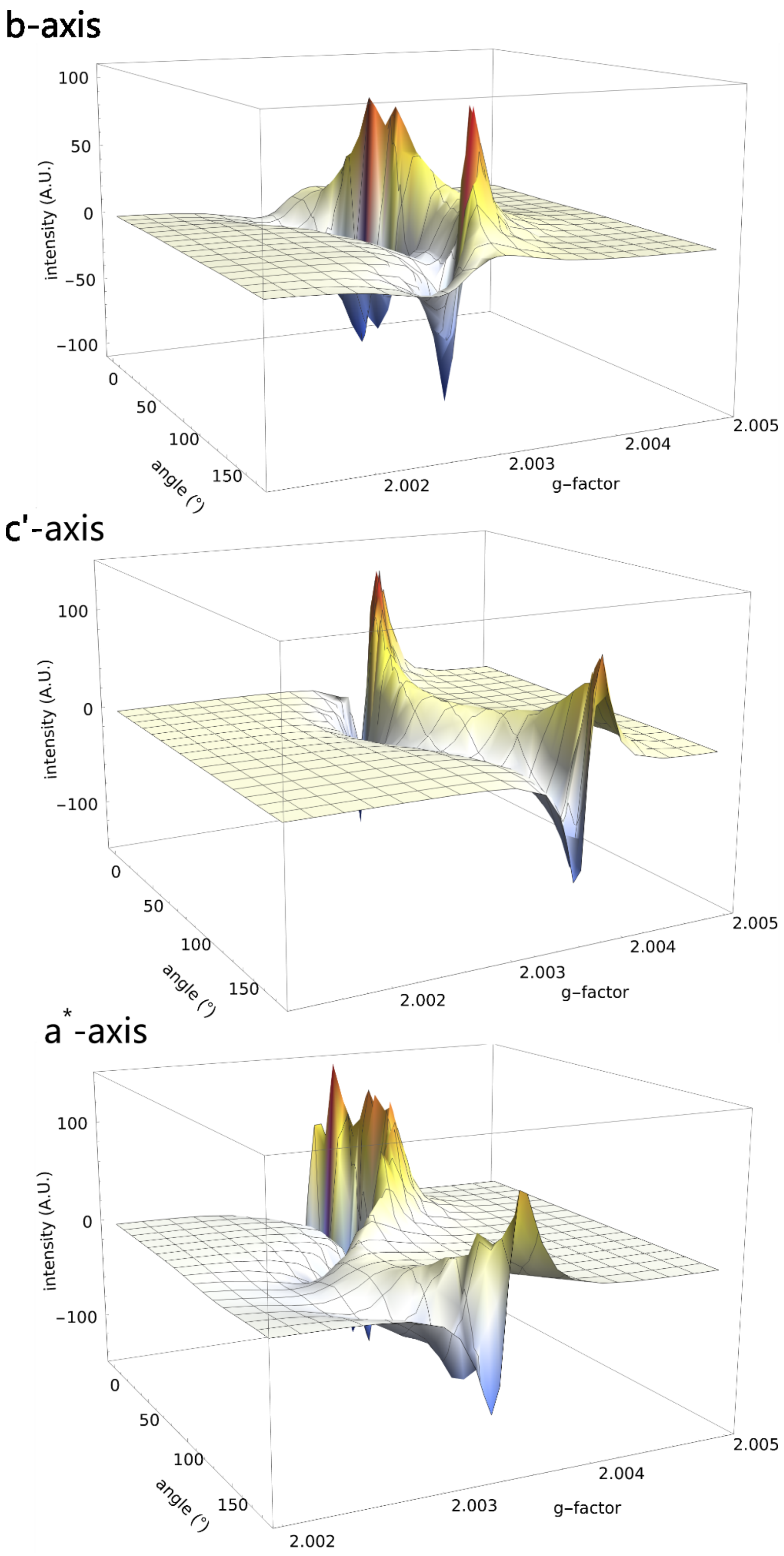

Figure S34 Angular dependence of the EPR spectra, recorded at RT, for **9·(TCNQ)$_4$** single crystal.

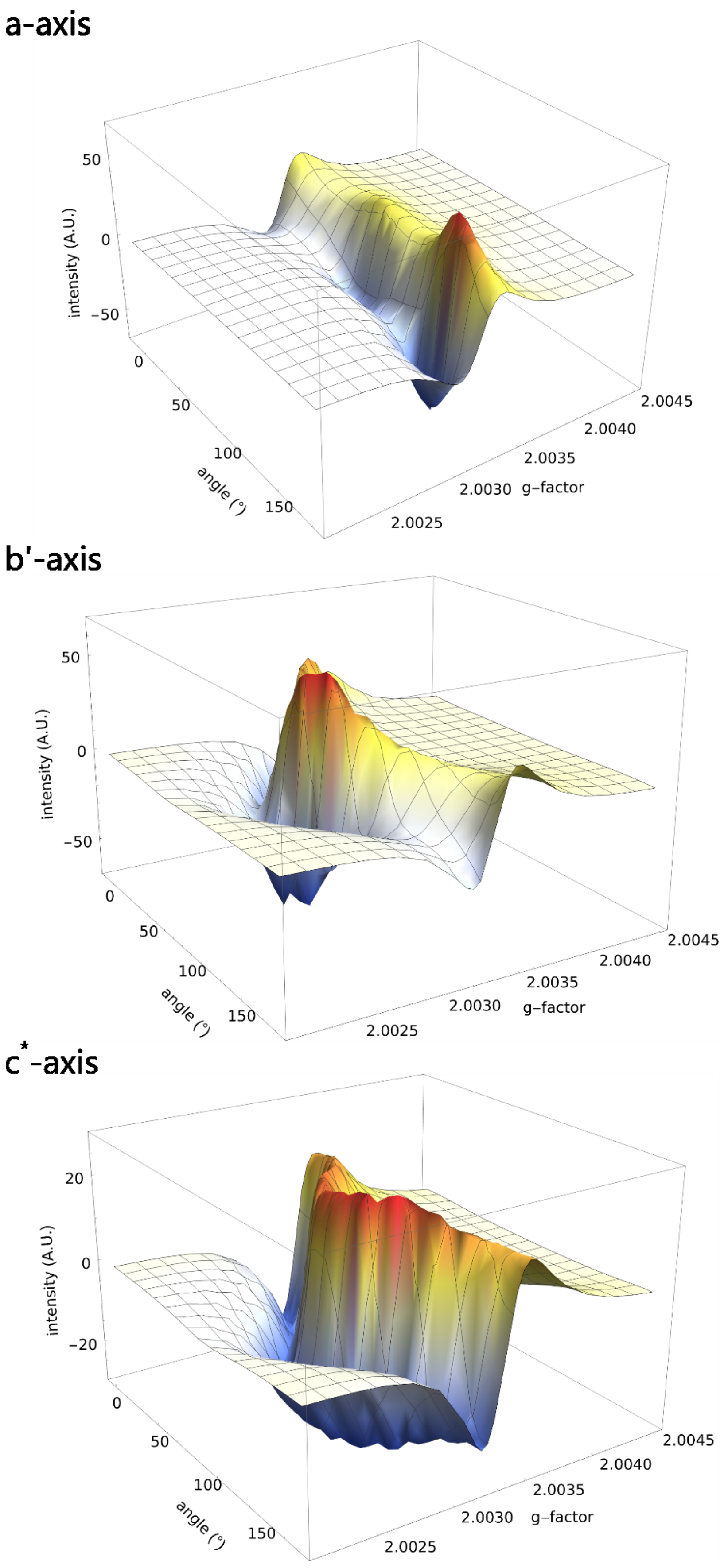

Figure S35 Angular dependence of the EPR spectra, recorded at RT, for **12·(TCNQ)$_2$** single crystal.

S7. IR spectra

1.1. Preparation and IR spectroscopy

All chemicals and solvents used were purchased from commercial sources (Merck, Alfa Aesar, Kemika), were of p.a. or reagent grade and were used without additional purification.

The compounds were prepared by a modification of our method for preparation of salts of semiquinone radicals with organic cations (Molčanov *et al.*, 2011, 2012, 2016, 2018a, 2019b). Powdered TCNQ (20.0 mg) was dissolved in cold acetone (5 mL, at 5 ºC), until the solution was approximately saturated. Into such a solution excess solid iodide salt of the appropriate organic cation was added (in the case of $\mathbf{1_2{\cdot}Br{\cdot}(TCNQ)_2}$ it was bromide). The beaker was sealed with paraffin and left overnight at room temperature; next day the acetone solution was decanted and black crystals of the $TCNQ^{\delta-}$ salt were washed with acetone and dried.

The infrared spectra were recorded with KBr pellets using a Bruker Alpha-T spectrometer in the 4000–350 cm^{-1} range.

IR data for compound $\mathbf{1_2{\cdot}Br{\cdot}(TCNQ)_2}$ (KBr, cm^{-1}): 3437m, 3053m, 3022m, 2668m, 2379w, 2290w, 2226w, 2202m, 2157vs, 1667w, 1608m, 1555vs, 1520m, 1492m, 1449m, 1435m, 1405w, 1328sh, 1297vs, 1266s, 1203w, 1113vs, 1075s, 1065s, 1039s, 952s, 883m, 852m, 837m, 795m, 775s, 752m, 692vs, 594m, 482m, 424m.

IR data for compound $\mathbf{2{\cdot}(TCNQ)_2}$ (KBr, cm^{-1}): 3488w, 3057w, 2671w, 2290w, 2225w, 2202s, 2177vs, 1631m, 1563s, 1516m, 1472m, 1451m, 1381m, 1336s, 1319sh, 1202m, 1160m, 1145m, 1136m, 1114m, 1078m, 1033m, 953m, 883m, 859sh, 839m, 838m, 826m, 802m, 770m, 750m, 699m, 593m, 579m, 480m, 433m.

IR data for compound $\mathbf{3{\cdot}(TCNQ)_2}$ (KBr, cm^{-1}): 3454w, 3102w, 3047w, 2665w, 2381w, 2287w, 2230w, 2202s, 2158vs, 1617w, 1555vs, 1513s, 1489s, 1450m, 1341sh, 1297vs, 1202m, 1115vs, 1101vs, 1079s, 1027s, 951s, 884m, 837m, 797m, 747m, 694s, 667s, 594s, 533m, 504m, 480m, 454m, 407w.

IR data for compound $\mathbf{4{\cdot}(TCNQ)_2}$ (KBr, cm^{-1}): 3451m, 3113m, 3047m, 2652w, 2387w, 2269w, 2230w, 2196s, 2160vs, 1665s, 1640m, 1593m, 1557s, 1522m, 1507m, 1450m, 1402m, 1374m, 1323s, 1305s, 1287vs, 1278vs, 1217m, 1182m, 1152sh, 1125sh, 1115s, 1078sh, 1052sh, 998m, 983m, 943m, 859m, 840m, 807m, 793m, 738m, 695s, 659m, 598m, 480w, 460w, 436w.

IR data for compound $\mathbf{5{\cdot}(TCNQ)_2}$ (KBr, cm^{-1}): 3441w, 3053w, 2923w, 2660w, 2386w, 2265w, 2233w, 2200s, 2171sh, 2158vs, 1652s, 1563vs, 1515s, 1396w, 1330vs, 1305vs, 1113vs, 1067vs, 947m, 887sh, 822m, 739w, 691s, 592m, 502w, 470w.

IR data for compound $\mathbf{6{\cdot}(TCNQ)_2}$ (KBr, cm^{-1}): 3480w, 3105w, 3057w, 2668w, 2384w, 2287w, 2227w, 2202s, 2158vs, 1947m, 1645m, 1618m, 1553s, 1515m, 1486m, 1445m, 1422m, 1332sh,

1311vs, 1275sh, 1205m, 1158sh, 1113vs, 1080sh, 1047s, 954s, 892m, 881m, 860m, 834s, 802m, 748m, 695s, 594m, 540m, 483m.

IR data for compound **7·(TCNQ)₂** (KBr, cm^{-1}): 3446m, 3055w, 3031w, 2924w, 2669w, 2380w, 2298w, 2228w, 2202m, 2167vs, 1944m, 1640m, 1557s, 1517s, 1511s, 1478m, 1332s, 1314s, 1293s, 1199m, 1130sh, 1114s, 1083m, 1044m, 951m, 882w, 842w, 856sh, 826m, 809m, 741w, 696s, 670m, 594m, 487m.

IR data for compound **8·(TCNQ)$_2$** (KBr, cm^{-1}): 3446m, 3081w, 3059w, 3035m, 2979w, 2258w, 2223w, 2183w, 1722vs, 1607m, 1549s, 1510m, 1469s, 1458s, 1442s, 1418s, 1403m, 1383m, 1365m, 1338m, 1309w, 1292w, 1231m, 1183s, 1172m, 1141m, 1121w, 1089m, 1019m, 925w, 874m, 851m, 800m, 774m, 738w, 722w, 693w, 667vs, 572m, 529w, 474w.

IR data for compound **9·(TCNQ)$_4$** (KBr, cm^{-1}): 3456w, 3126w, 2382w, 3062w, 2263w, 2198vs, 2176vs, 1638w, 1590s, 1561s, 1526m, 1513m, 1445m, 1413s, 1377m, 1344s, 1335s, 1305s, 1201w, 1184m, 1157m, 1133m, 1115m, 1078sh, 951w, 841s, 700s, 669m, 599m, 478w

IR data for compound **10·(TCNQ)$_3$** (KBr, cm^{-1}): 3444m, 3120w, 3051w, 2924w, 2667w, 2294w, 2226w, 2190s, 2169vs, 1637m, 1564vs, 1516s, 1460m, 1382sh, 1353vs, 1275s, 1224m, 1204, 1167vs, 1113sh, 957m, 895m, 862m, 831m, 814m, 798m, 749m, 705s, 609m, 601m, 586m, 597m, 474m.

IR data for compound **11$_2$·I·(TCNQ)$_2$** (KBr, cm^{-1}): 3442m, 3053w, 3005m, 2931w, 2380w, 2306w, 2202m, 2171vs, 2141s, 1624w, 1555m, 1503m, 1492m, 1455m, 1421w, 1370m, 1347sh, 1338m, 1320s, 1295m, 1280m, 1204m, 1179m, 1104s, 1028m, 984m, 952m, 902w, 851w, 831w, 810w, 797m, 690m, 665s, 595m, 536w, 480w, 415w.

IR data for compound **12·(TCNQ)$_2$** (KBr, cm^{-1}): 3427m, 3051m, 2659w, 2261w, 2218w, 2200m, 2178m, 2150vs, 1553s, 1520s, 1504s, 1473m, 1363m, 1295vs, 1278vs, 1204m, 1113vs, 1077s, 1031s, 952m, 880m, 859sh, 836m, 782m, 748w, 690s, 655m, 594m, 575m, 535m, 480w.

IR data for compound **13·(TCNQ)$_2$** (KBr, cm^{-1}): 3451w, 3057w, 2925w, 2668w, 2379w, 2287w, 2230w, 2202s, 2169vs, 1945m, 1633m, 1557s, 1518m, 1493m, 1380m, 1334vs, 1317vs, 1302vs, 1204m, 1191m, 1156sh, 1132vs, 1111vs, 1079s, 1052s, 951s, 883m, 852w, 839m, 799m, 768m, 750m, 698s, 677s, 595m, 481m, 443m.

IR data for compound **14·(TCNQ)$_2$** (KBr, cm^{-1}): 3459w, 3057w, 2665w, 2384w, 2266w, 2229w, 2204m, 2169vs, 1628w, 1580m, 1561m, 1525m, 1505m, 1475m, 1447m, 1433m, 1418m, 1359m, 1337s, 1315s, 1236m, 1206w, 1181m, 1157m, 1144m, 1129m, 1117sh, 953m, 843m, 715m, 698m, 594m, 482w.

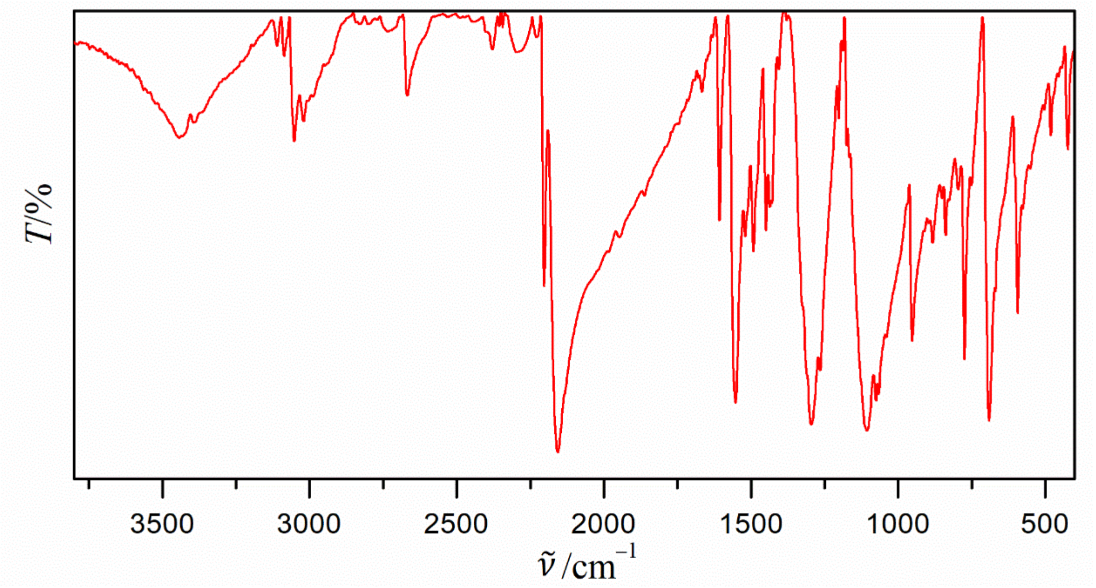

Figure S36 IR spectrum of $\mathbf{1_2{\cdot}Br{\cdot}(TCNQ)_2}$.

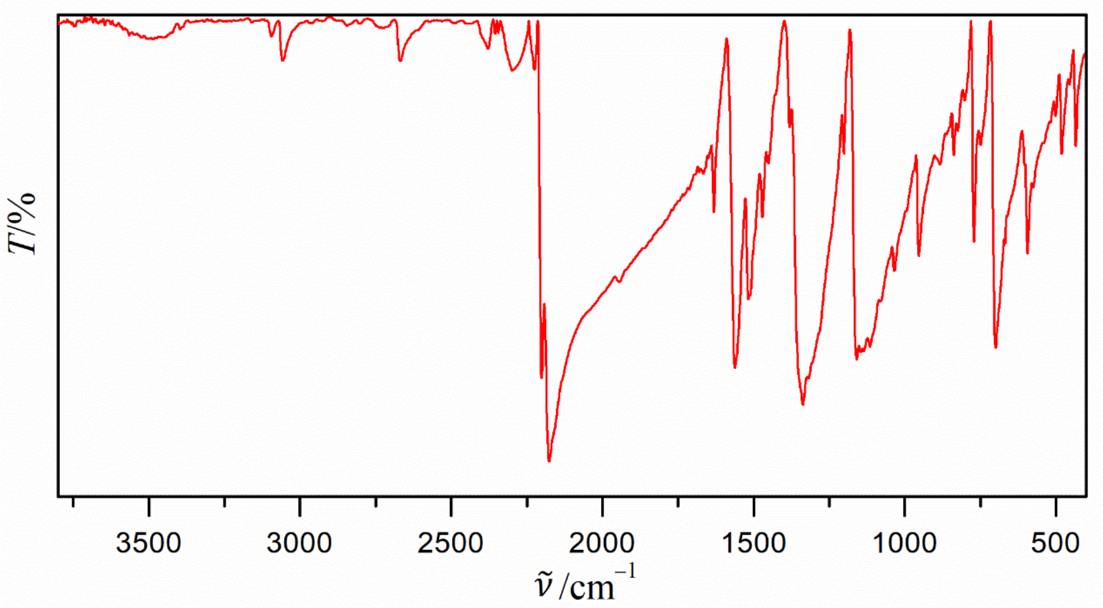

Figure S37 IR spectrum of $\mathbf{2{\cdot}(TCNQ)_2}$.

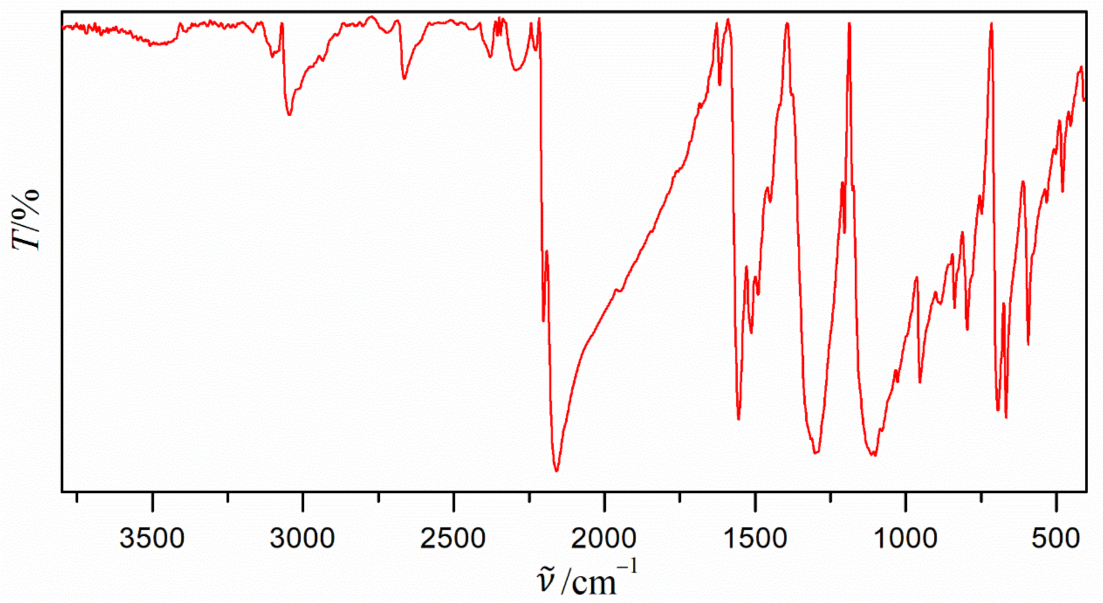

Figure S38 IR spectrum of **3·(TCNQ)$_2$**.

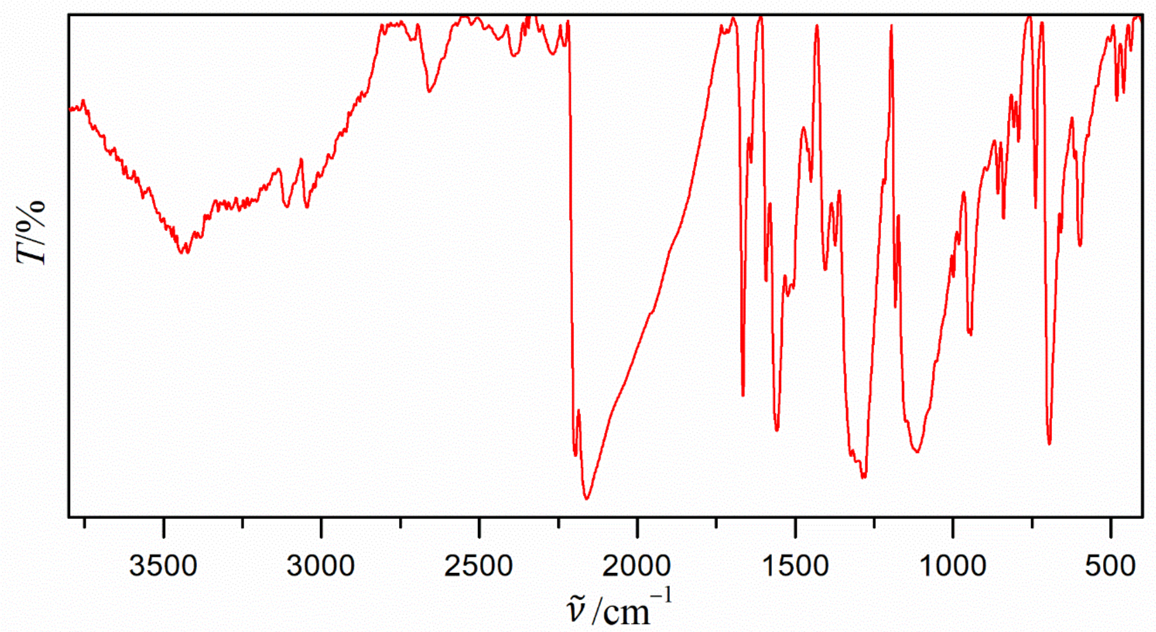

Figure S39 IR spectrum of **4·(TCNQ)$_2$**.

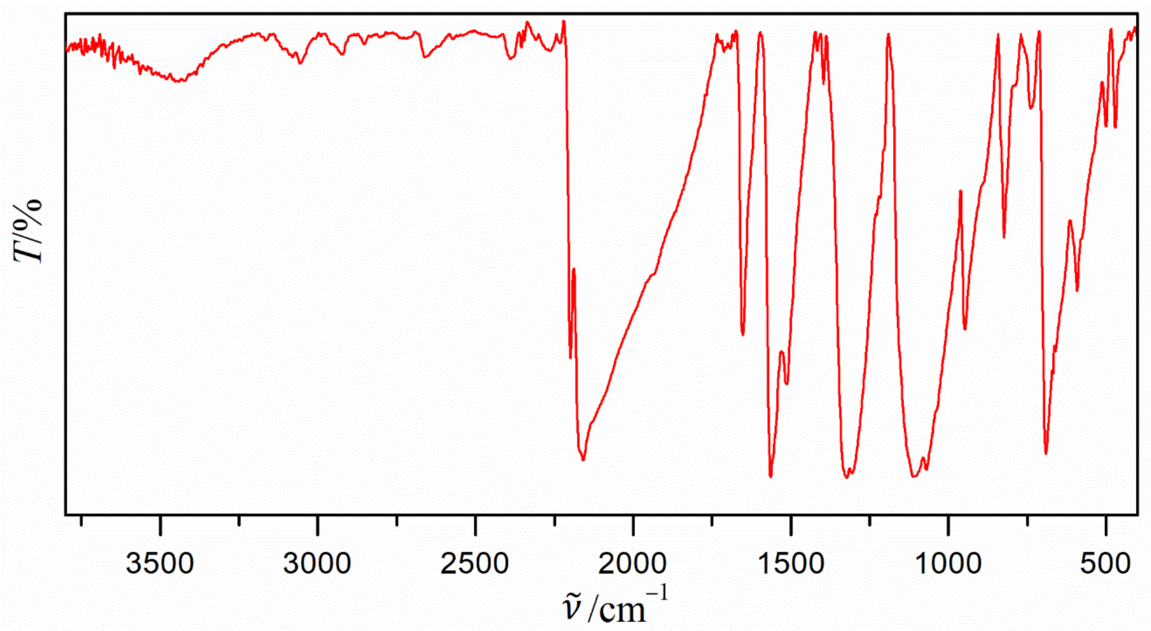

Figure S40 IR spectrum of **5·(TCNQ)$_2$**.

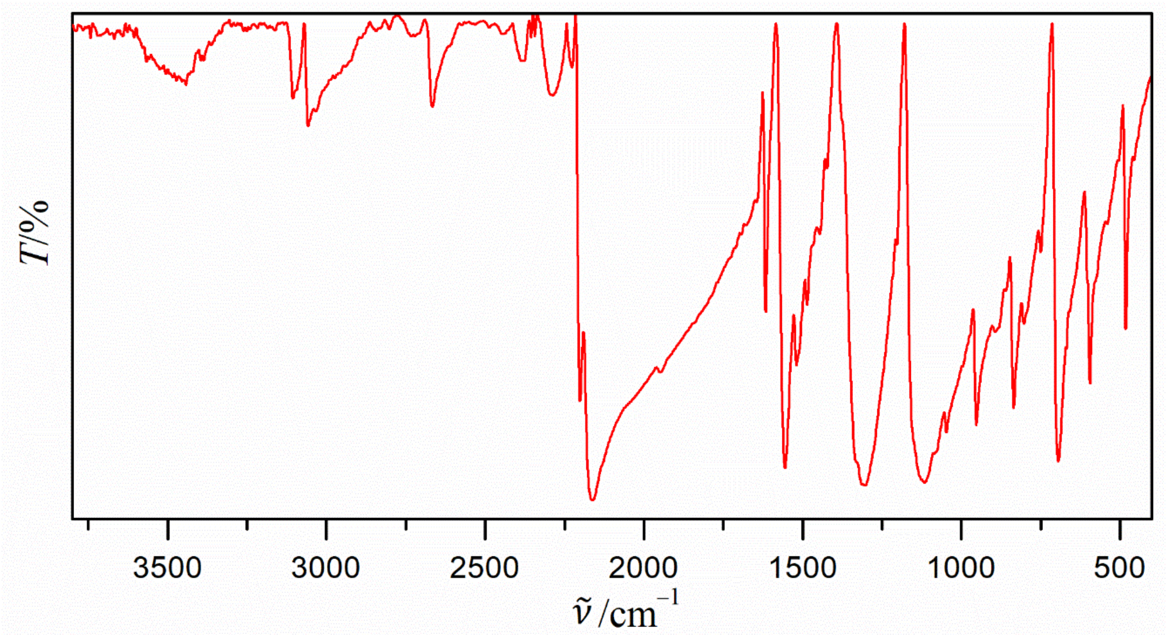

Figure S41 IR spectrum of **6·(TCNQ)$_2$**.

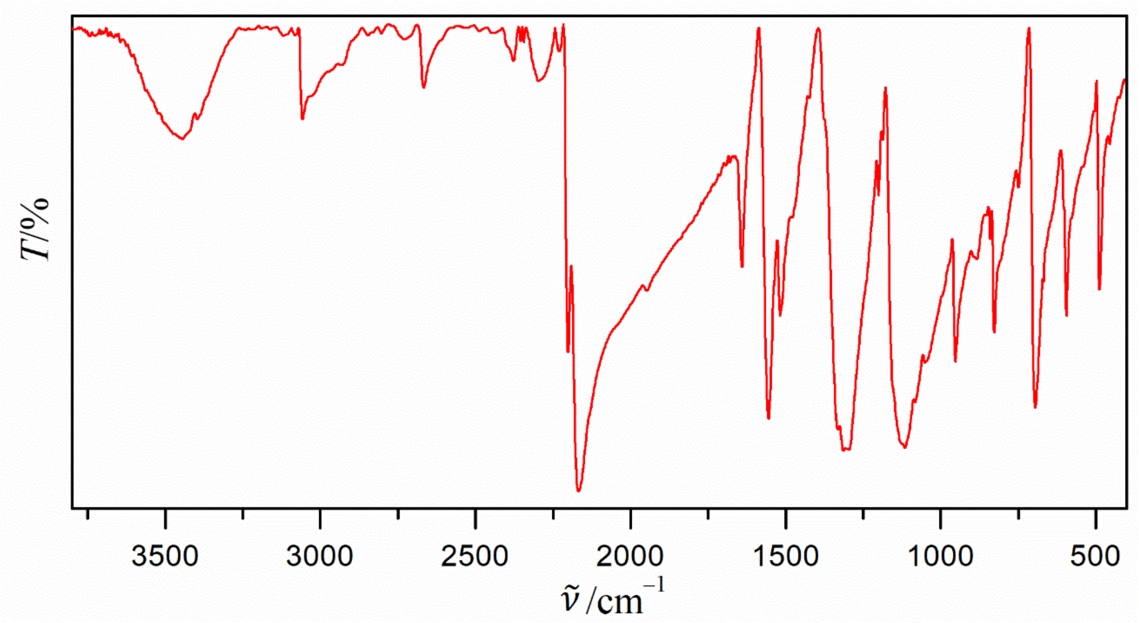

Figure S42 IR spectrum of **7·(TCNQ)$_2$**.

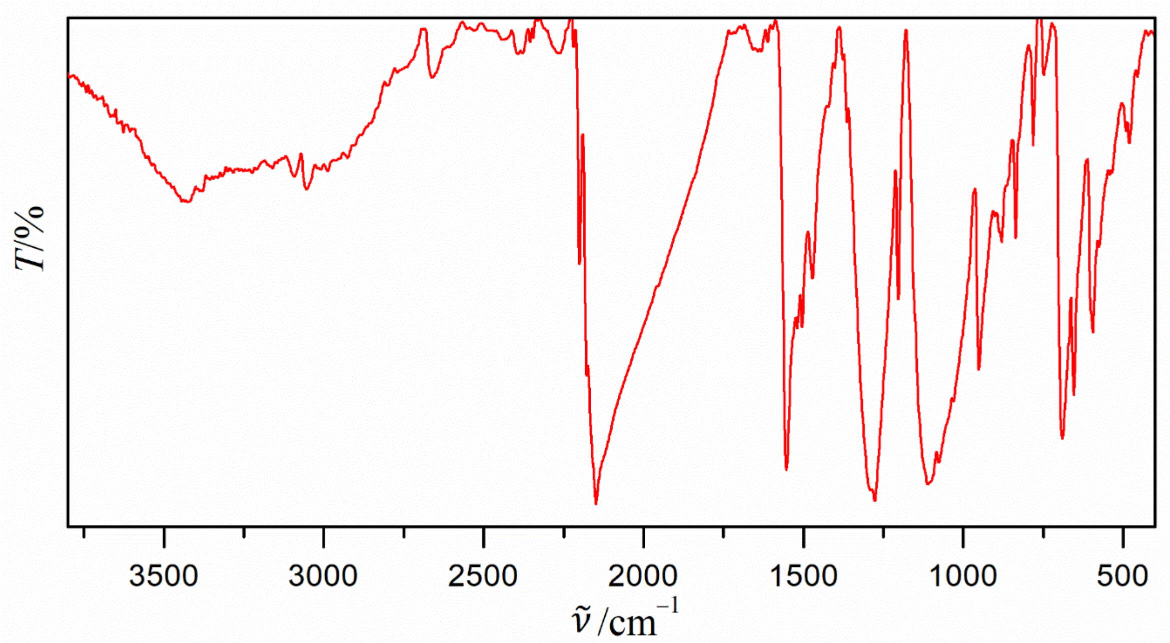

Figure S43 IR spectrum of **8·(TCNQ)$_2$**.

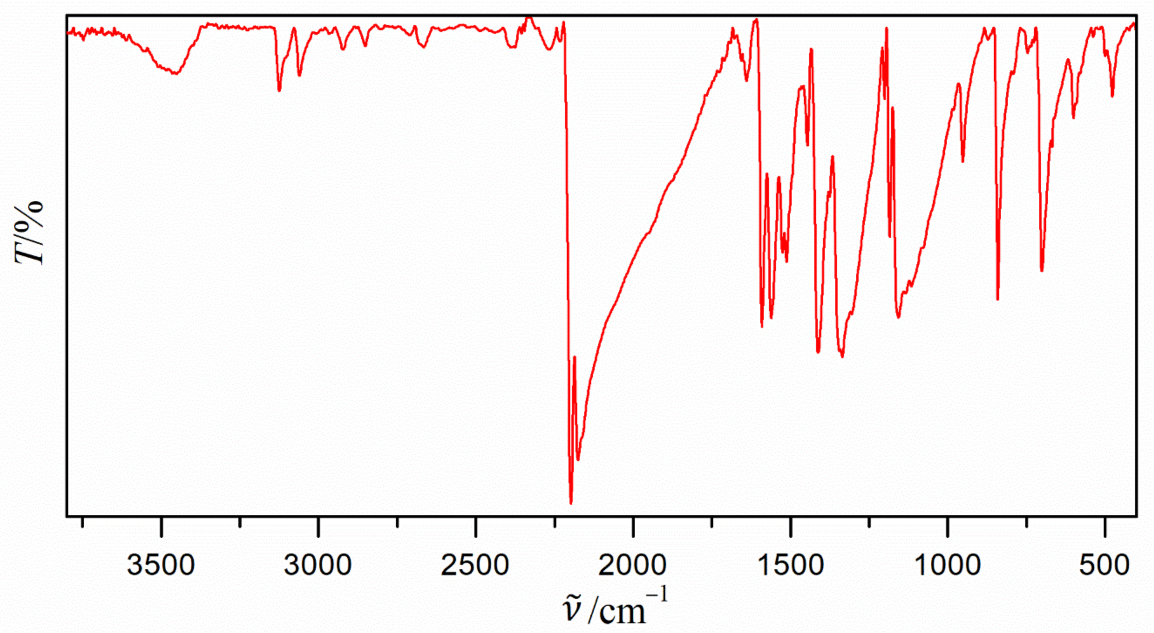

Figure S44 IR spectrum of **9·(TCNQ)$_4$**.

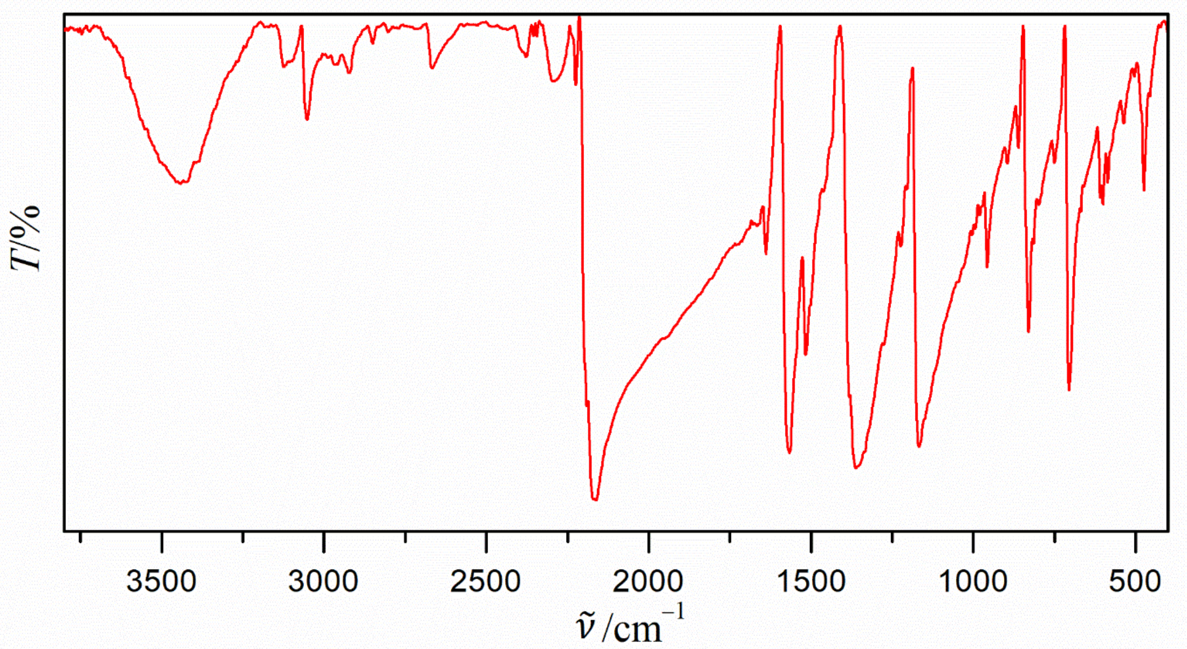

Figure S45 IR spectrum of **10·(TCNQ)$_3$**.

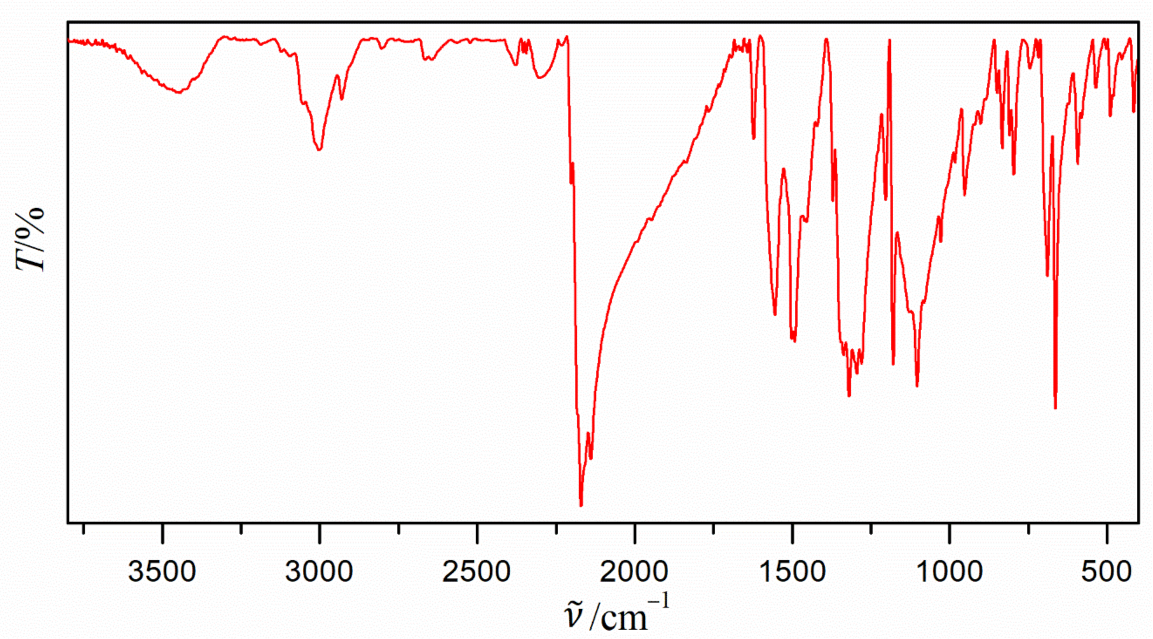

Figure S46 IR spectrum of **11_2·I·(TCNQ)$_2$**.

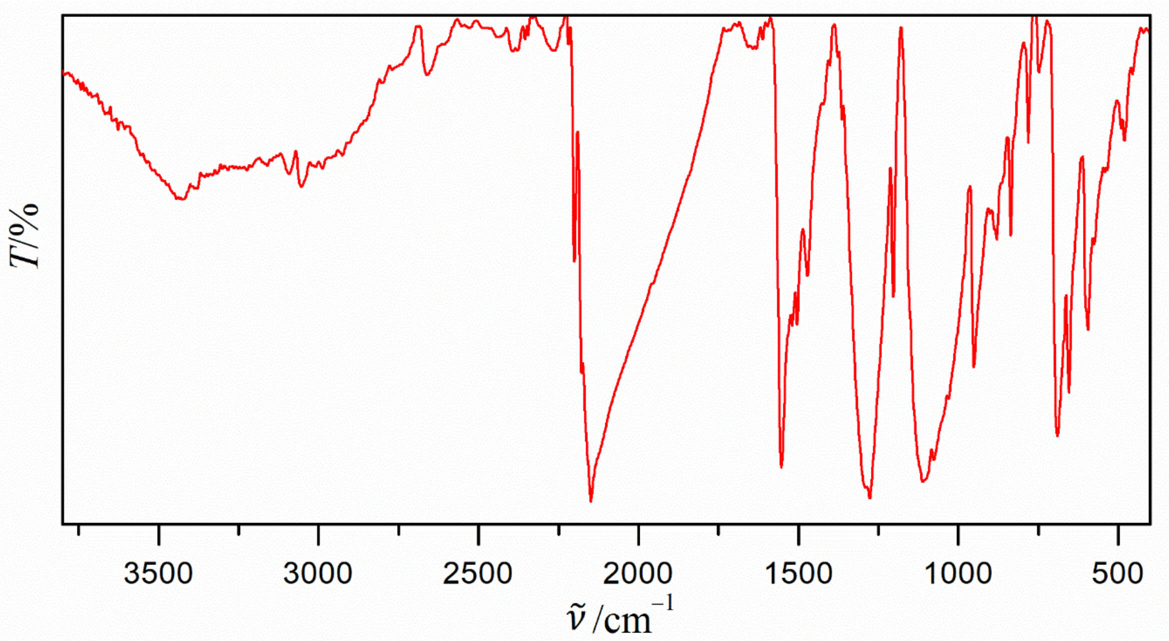

Figure S47 IR spectrum of **12·(TCNQ)$_2$**.

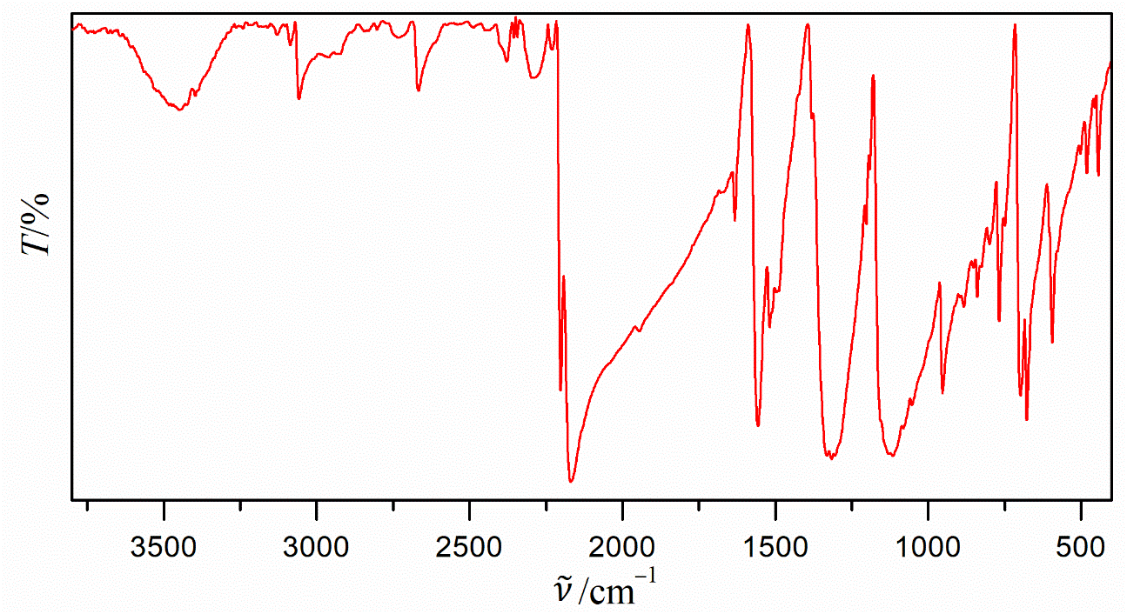

Figure S48 IR spectrum of **13·(TCNQ)2**.

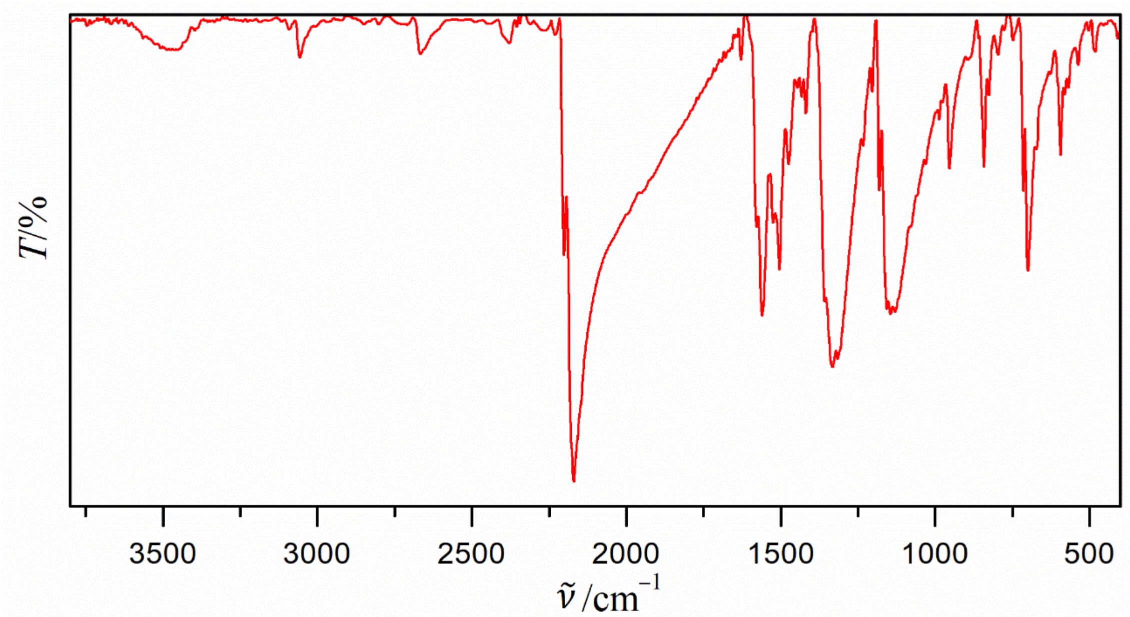

Figure S49 IR spectrum of **14·(TCNQ)2**.

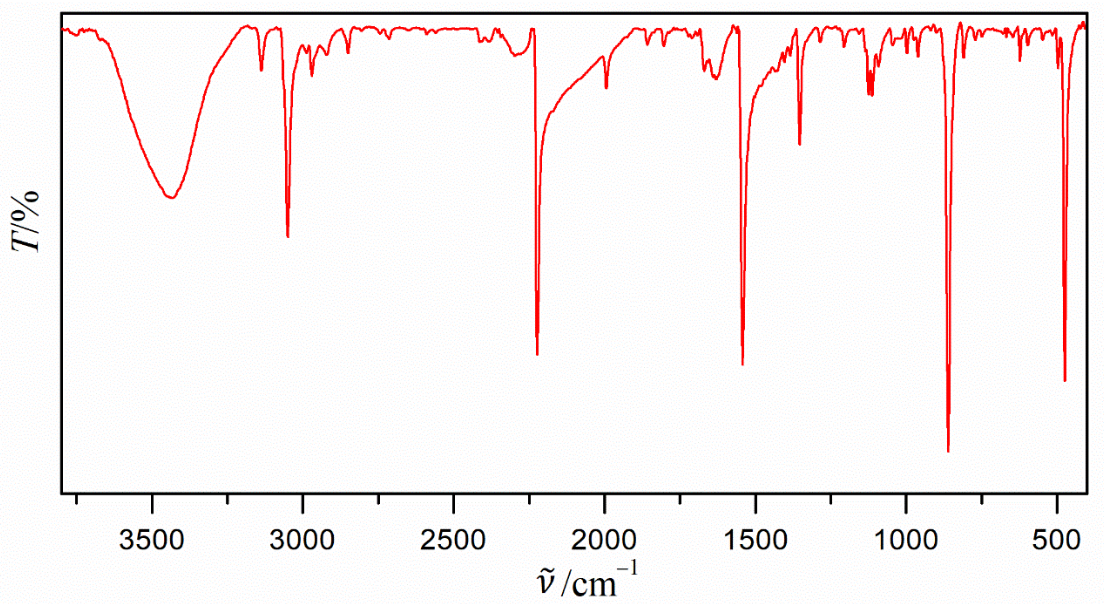

Figure S50 IR spectrum of neutral TCNQ.

S8. Powder diffractograms

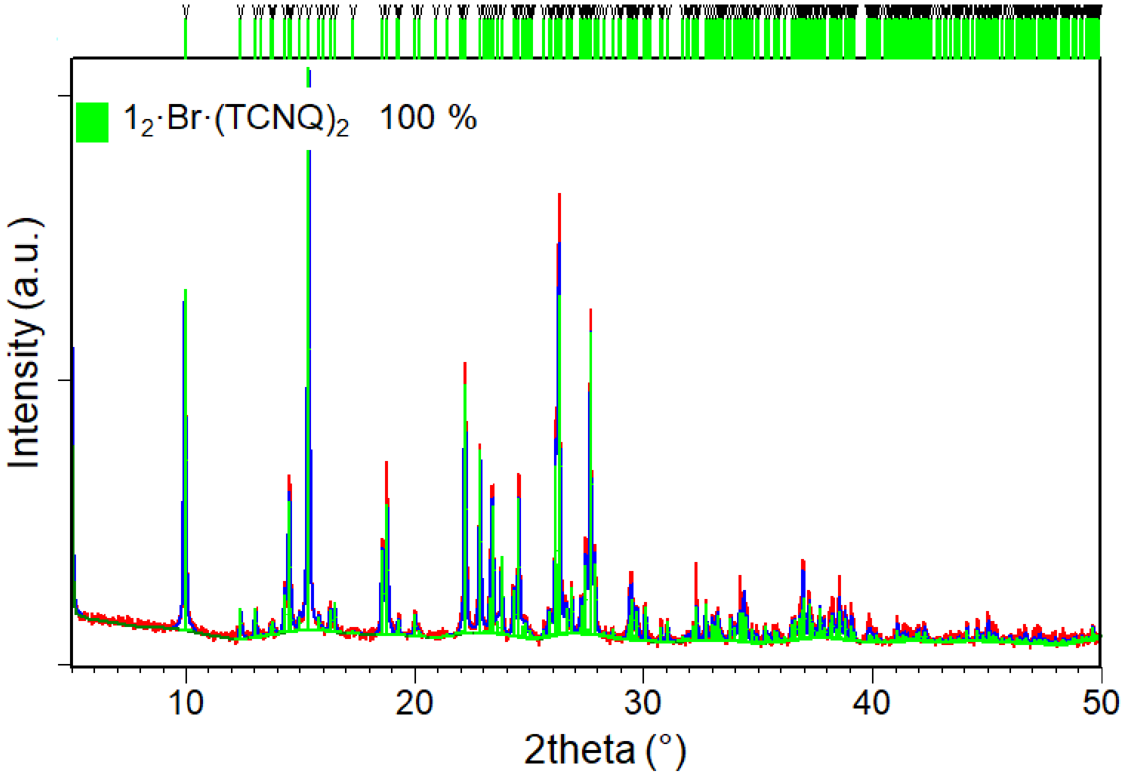

Figure S51 Rietveld refinement on powder X-ray diffraction data for sample 12·Br·(TCNQ)2. Experimental data are given by a red line, the calculated pattern is shown in blue. Green vertical marks show the positions of Bragg reflections.

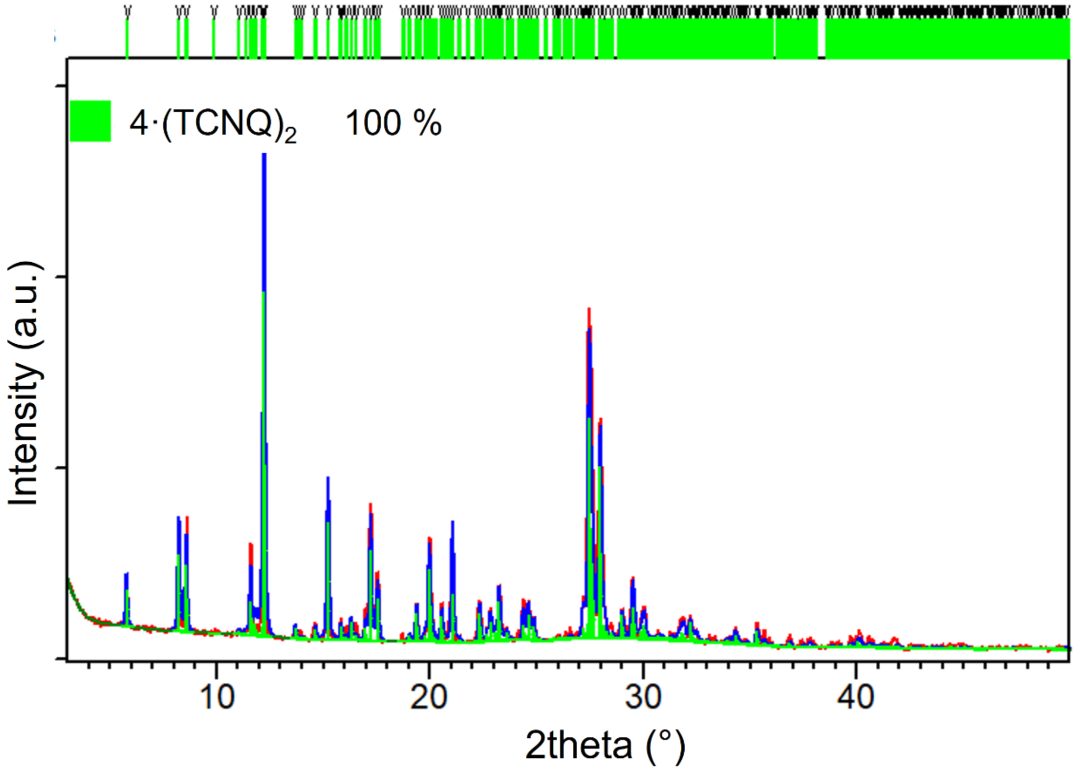

Figure S52 Rietveld refinement on powder X-ray diffraction data for sample **4·(TCNQ)₂**. Experimental data are given by a red line, the calculated pattern is shown in blue. Green vertical marks show the positions of Bragg reflections.

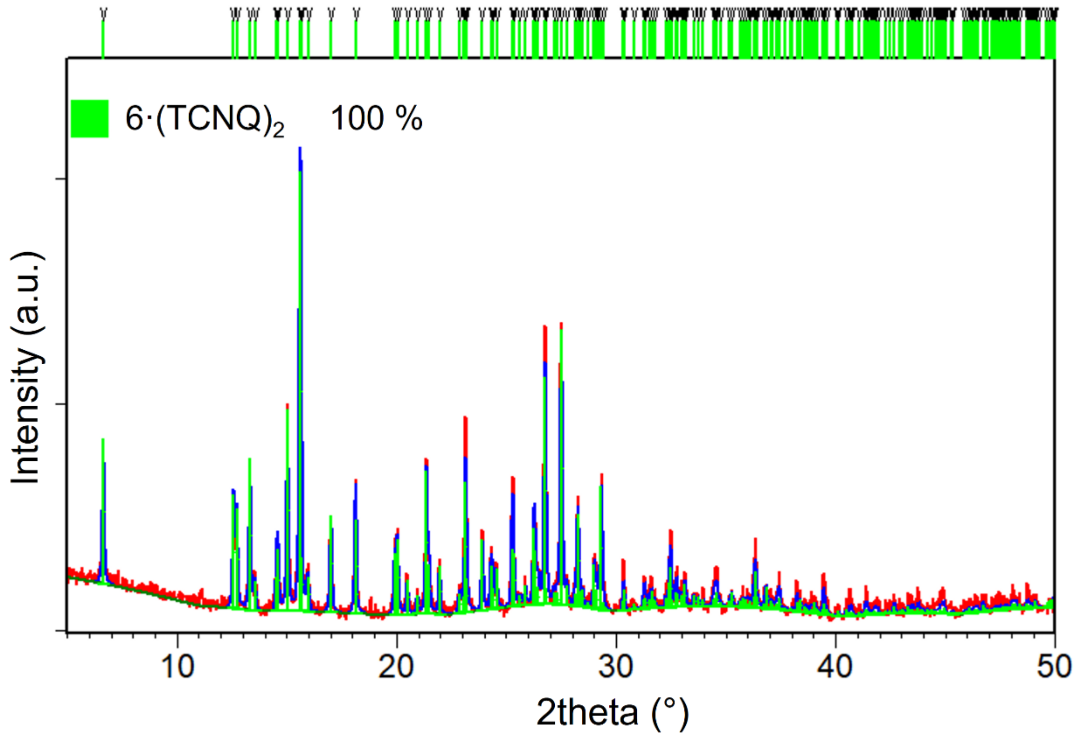

Figure S53 Rietveld refinement on powder X-ray diffraction data for sample **6·(TCNQ)₂**. Experimental data are given by a red line, the calculated pattern is shown in blue. Green vertical marks show the positions of Bragg reflections.

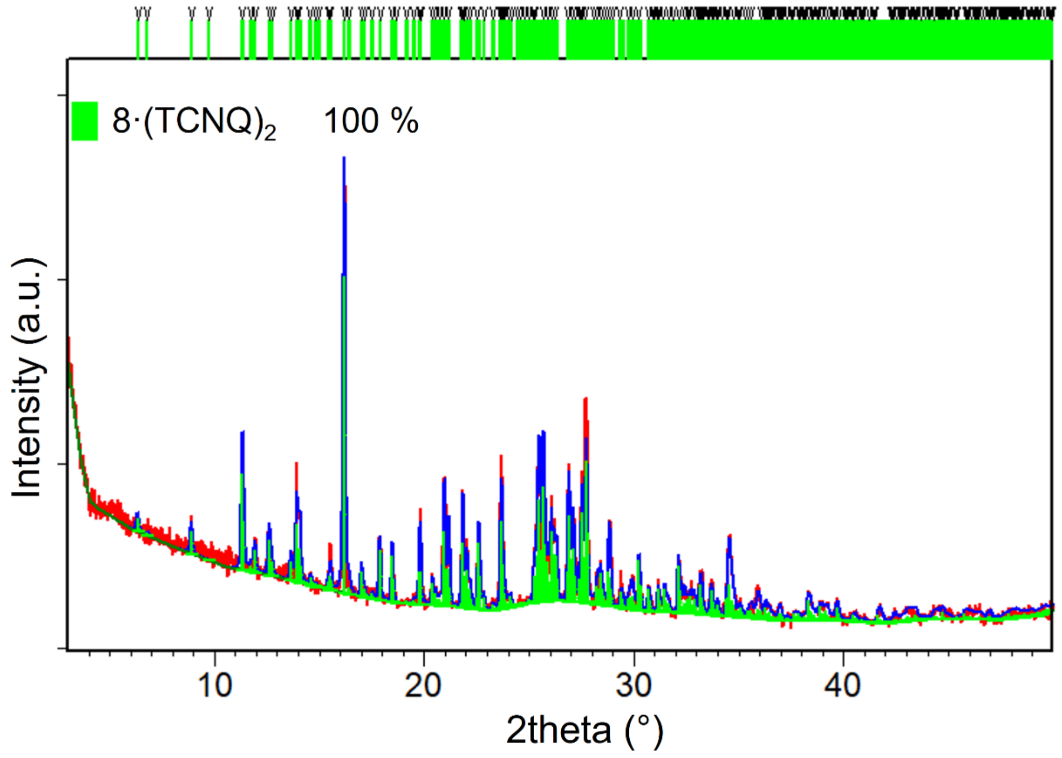

Rietveld refinement on powder X-ray diffraction data for sample **8·(TCNQ)$_2$**. Experimental data are given by a red line, the calculated pattern is shown in blue. Green vertical marks show the positions of Bragg reflections.

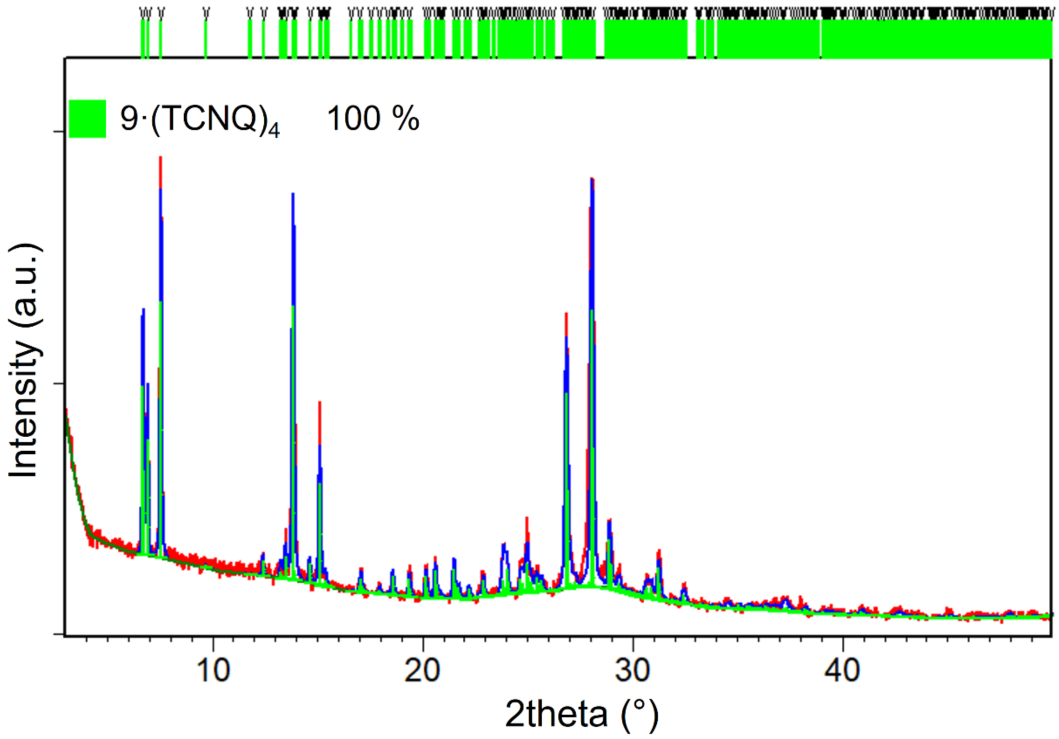

Figure S54 Rietveld refinement on powder X-ray diffraction data for sample **9·(TCNQ)**$_4$. Experimental data are given by a red line, the calculated pattern is shown in blue. Green vertical marks show the positions of Bragg reflections.

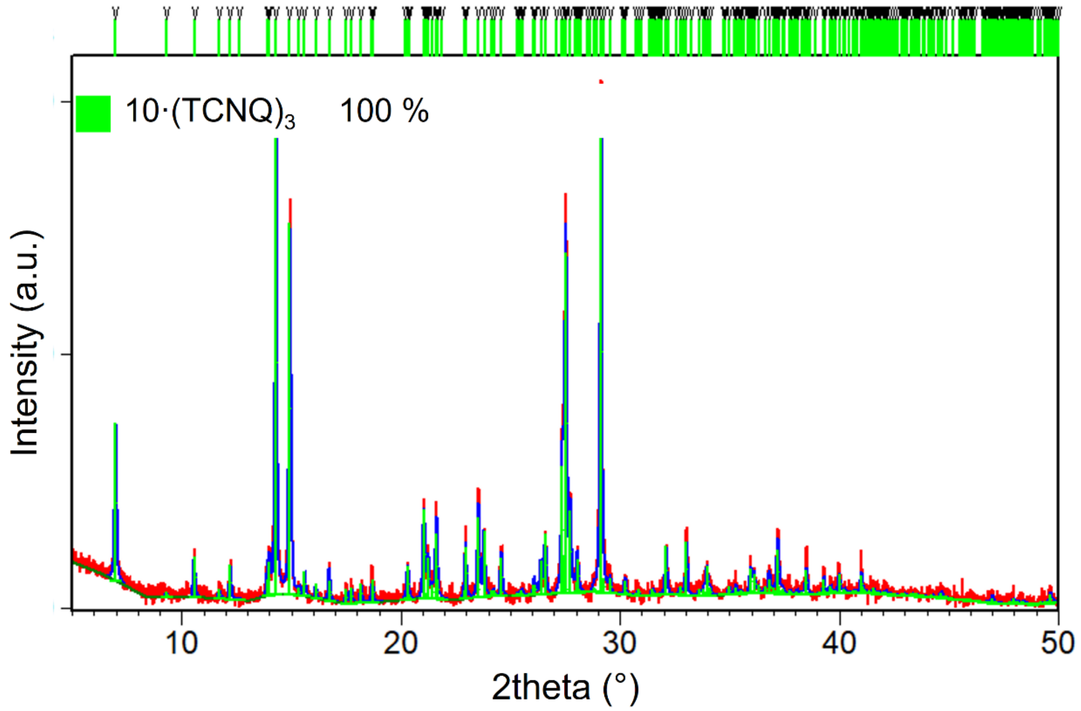

Figure S55 Rietveld refinement on powder X-ray diffraction data for sample **10·(TCNQ)₃**. Experimental data are given by a red line, the calculated pattern is shown in blue. Green vertical marks show the positions of Bragg reflections.

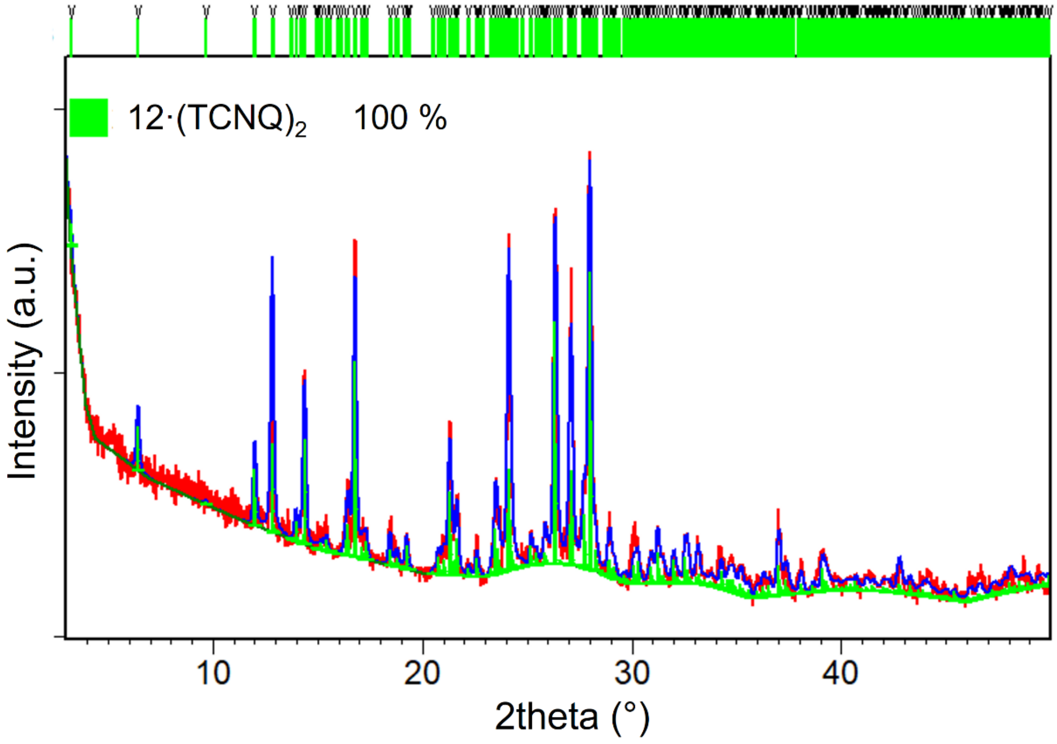

Figure S56 Rietveld refinement on powder X-ray diffraction data for sample **12·(TCNQ)$_2$**. Experimental data are given by a red line, the calculated pattern is shown in blue. Green vertical marks show the positions of Bragg reflections.
